# Redox-dependent synaptic clustering of gephyrin

**DOI:** 10.1038/s41467-026-77147-2

**Published:** 2026-09-03

**Authors:** Maria-Theresa Gehling, Emanuel H. W. Bruckisch, Fynn R. Eggersmann, Konrad Benting, Lianne J. H. C. Jacobs, Elmar Behrmann, Jan Riemer, Peter Kloppenburg, Filip Liebsch, Günter Schwarz

**Affiliations:** 1 Institute of Biochemistry, Department of Chemistry and Biochemistry, University of Cologne, Cologne, Germany; 2 Institute for Zoology, Department of Biology, University of Cologne, Cologne, Germany; 3 Cologne Excellence Cluster on Cellular Stress Responses in Aging-Associated Diseases (CECAD), University of Cologne, Cologne, Germany; 4 Center for Molecular Medicine Cologne (CMMC), University of Cologne, Cologne, Germany

**Keywords:** Neurochemistry, Synaptic plasticity

## Abstract

Reactive oxygen species (ROS) have been demonstrated to play central functions as signaling molecules thus extending their role beyond oxidative stress in disease and aging. At inhibitory synapses the scaffolding protein gephyrin clusters glycine and GABA type A receptors. Postsynaptic gephyrin clustering is regulated by post-translational modifications such as phosphorylation, S-palmitoylation, and S-nitrosylation, which all critically impact its scaffolding function. Here, we show that oxidation of surface-exposed cysteine residues in gephyrin triggered reversible, synaptic multimerization providing more receptor binding sites and leading to proteolytic protection. Cys419, located at the previously published E-domain SDII interface important for liquid-liquid phase separation, was identified as a critical residue further regulating liquid-liquid phase separation of gephyrin in a redox-dependent manner. We also observed that surface-exposed cysteines are required to maintain fast miniature inhibitory postsynaptic current rise times, supporting the functional relevance of gephyrin redox regulation. Collectively, our findings suggest that cysteines in gephyrin regulate synaptic localization and clustering as redox-switches, thereby establishing a so far undefined link between neuronal and metabolic activity at inhibitory synapses.

## Abstract (web summary)

Gehling et al show that cysteines in gephyrin regulate synaptic localization and clustering as redox-switches, thereby establishing a so far undefined link between neuronal and metabolic activity at inhibitory synapses.

## Introduction

Neuronal activity is connected to oxygen consumption and the production of reactive oxygen species (ROS)^1^, that were initially considered as neurotoxic^2^. However, recently, redox signaling has emerged as an important pathway for modulating synaptic plasticity. For instance, in molecular layer interneurons, ROS can enhance neurotransmission at inhibitory synapses^3,4^, which was attributed to the recruitment of α3-containing GABA type A receptors (GABA_A_R) to the synapse. This mechanism involves a complex cascade following Ca^2+^ entry through NMDA-type glutamate receptors, which increases neuronal nitric oxide synthase activity. Elevated NO levels trigger cGMP production and protein kinase G activation, which in turn stimulates NAPDH oxidase. Topical NMDA application in the neocortex results in ROS production via the same pathway^5^. Finally, elevated ROS levels activate protein kinase C, which results in the recruitment of α3-containing GABA_A_Rs^4^. Furthermore, in hippocampal pyramidal neurons H_2_O_2_ can increase GABA_A_R-mediated tonic currents via an extracellular oxidative reaction, mediated by NAPDH oxidase, while synaptic currents remained unaffected^6^.

Importantly, maintaining the proper relation of ROS production and clearance is crucial for healthy brain function, and disruptions in ROS homeostasis leading to oxidative stress have been linked to various neuronal diseases^7,8^. Consequently, unraveling the underlying molecular link between ROS production and neuronal activity is of great interest for understanding synaptic plasticity and the onset of pathological processes.

One key component at inhibitory synapses is the bifunctional protein gephyrin. Gephyrin is crucial for the activity of molybdenum cofactor (Moco)-dependent enzymes involved in basic metabolism. The conserved N-terminal G-domain and C-terminal E-domain of gephyrin catalyze the last two steps of Moco biosynthesis^9^. In addition, in the CNS gephyrin serves as the main scaffolding protein at inhibitory synapses, where it clusters glycine receptors (GlyRs) and a subset of GABA_A_Rs to facilitate efficient neurotransmission^10,11^. The synaptic clustering requires the self-assembly of gephyrin through E-domain dimerization and G-domain trimerization^12^. The interaction between receptors and gephyrin takes place between the intracellular domain (ICD) of one receptor subunit and the gephyrin E-domain^12^. The gephyrin C-domain links the G- and E-domain, providing flexibility as well as sites for post-translational modifications (PTMs) that regulate receptor binding and clustering^13,14^. The proteolytic cleavage within the gephyrin C-domain by the calcium-dependent protease calpain^15,16^ is an irreversible modification, which is important for gephyrin homeostasis and links excitatory calcium signaling to the shaping of inhibitory gephyrin scaffolds^15,17^. Additionally, gephyrin phosphorylation^18^, acetylation, and SUMOylation^19^ regulate gephyrin clustering and can shape the structure and function of inhibitory synapses. Moreover, gephyrin participates in a complex network of protein interactions crucial for signal transmission and neuronal plasticity^20,21^. Thus, dysfunction of gephyrin has detrimental consequences for both metabolic and neuronal functions, ranging from delayed development to seizures and early childhood death^22–24^.

Recent studies revealed that gephyrin regulation by PTMs, including those targeting cysteines, is essential for synaptic plasticity^25,26^. Palmitoylation at Cys212 and Cys284 was identified to increase synaptic gephyrin clusters^26^, while S-nitrosylation resulted in smaller synaptic clusters^25^.

Recently, liquid-liquid phase separation (LLPS) has emerged as a novel mechanism for creating subcellular microdomains and has been proposed as a possible mechanism for the formation of protein assemblies at presynaptic terminals and postsynaptic densities^27^. Several proteins at excitatory postsynaptic densities can undergo LLPS^28,29^ and gephyrin, together with model proteins of GlyR/GABA_A_Rs, can be organized into concentrated, droplet-like compartments, without the need for membranes^30^. Additionally, proteins at excitatory and inhibitory postsynaptic densities spontaneously segregate into distinct condensed molecular assemblies^31^. Importantly, gephyrin condensation is critically regulated by its central intrinsically disordered region (IDR) linker through conformational collapse and electrostatic interdomain interactions, rather than IDR–IDR interactions, with other iPSD components modulating its stability and localization via a thermodynamically driven network of intermolecular interactions^32^. Recent structural work has begun to shed light on the molecular basis of gephyrin condensation. Using single-particle cryo-EM, our laboratory demonstrated that gephyrin E-domain dimerization drives the formation of filamentous assemblies in which adjacent dimers are linked through Z-shaped interfaces between their subdomain II (SDII) regions, identifying these SDII–SDII’ interactions as the fundamental structural unit underlying LLPS^33^. Disruption of this interface, whether through SDII deletion, epilepsy-associated pathogenic variants, or charge neutralization, abolishes filament formation, in vitro phase separation, and synaptic receptor clustering, establishing gephyrin E-domain filaments as the structural foundation of inhibitory postsynaptic density organization. The importance of this finding is underlined by a second paper published recently, reproducing these structures^34^. Moreover, while gephyrin’s capacity for LLPS may be subject to redox regulation, the functional roles of its surface cysteines remain incompletely characterized. Here, we investigated the relationship between redox signaling and gephyrin surface cysteines, extending beyond previously described S-nitrosylation and S-palmitoylation events.

Our results demonstrate a dynamic redox-dependent regulatory mechanism for synaptic gephyrin clustering: Oxidation strengthened clustering and resulted in fast miniature inhibitory postsynaptic current (mIPSC) rise times. As a result of ROS-induced gephyrin multimerization, the local number of receptor binding sites increased, which is distinct from a previously reported redox-dependent recruitment of α3-containing GABA_A_Rs to strengthen inhibitory neurotransmission^3,4^. In conclusion, our study establishes a link between inhibitory synapses and ROS, and offers a feedforward pathway through redox-dependent synaptic clustering of gephyrin.

## Results

### Surface cysteines of gephyrin are required for synaptic function

**Fig. 1:**
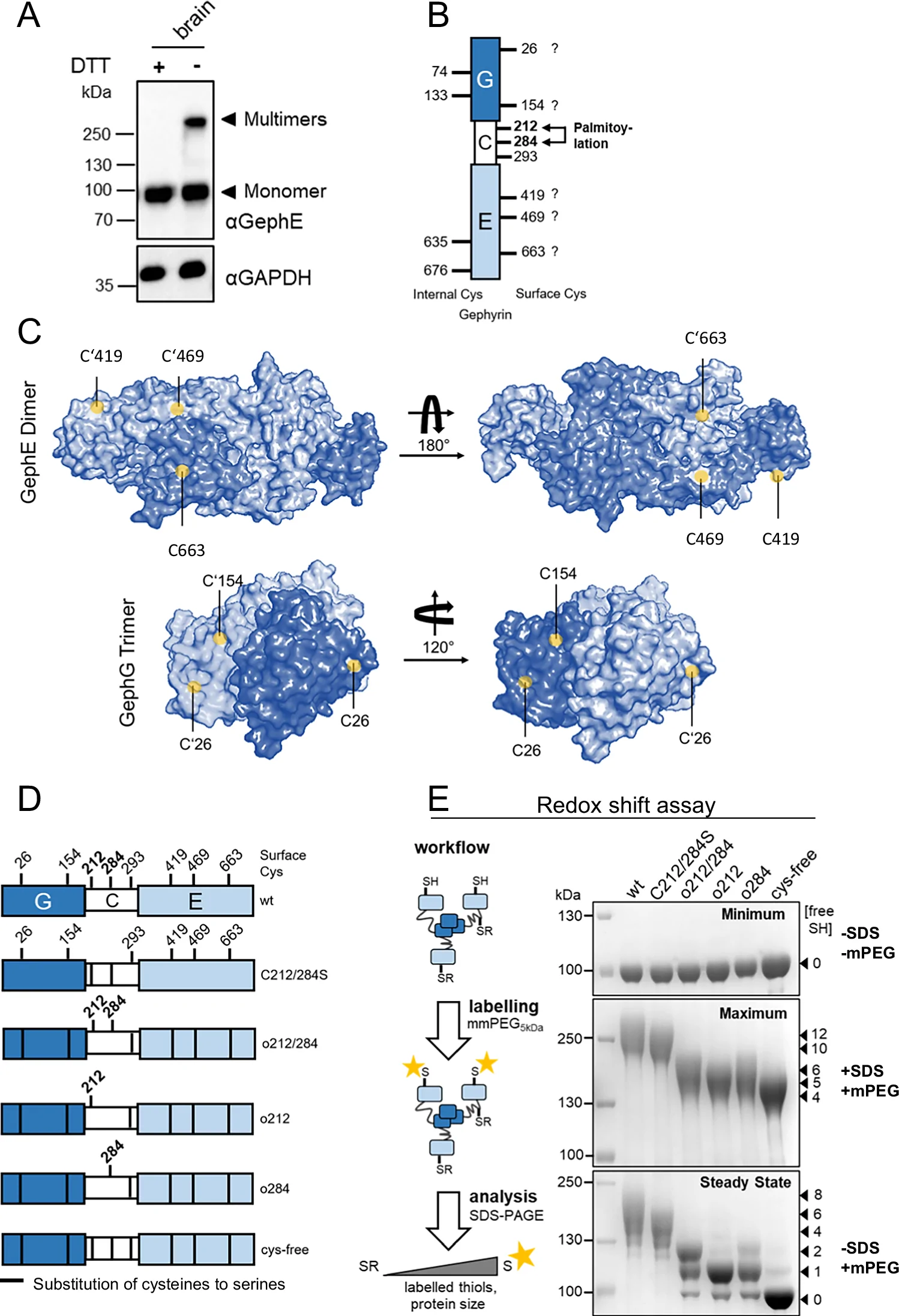
Role of surface-exposed cysteines of gephyrin. **A** Representative Western blot analysis of tissue lysates generated from mice that have been perfused with NEM under reducing (DTT) or non-reducing conditions. Monomeric and multimeric gephyrin marked with arrows, *n* = 6 mice. **B** Domain structure of gephyrin. G G-domain, C C-domain, E E-domain. Positions of the eight potential surface-exposed and four internal cysteines are highlighted. **C** Crystal structures of trimeric G-domain (1IHC^37^) and dimeric E-domain (2FU3^36^). Surface cysteines highlighted in yellow. Numbering according to isoform P1. **D** Genetically engineered surface cysteine variants used in this study. Cysteines were substituted with serines at indicated positions. All internal cysteines remained unchanged. **E** Representative gels from redox-shift assay with cysteine variants. Workflow: In this assay free thiols (–SH) are labelled with a maleimide-PEG-5kDa-tag (mPEG) increasing the size in SDS-PAGE. Coomassie staining: Labelled, free-thiols cysteines indicated with arrows. As controls mPEG and SDS were left out (Minimum) and both were added to additionally label the 4 internal cys (Maximum). For the steady state redox-status mPEG but no SDS was used, *N* = 3 technical replicates. Source data are provided as a Source Data file.

A crucial aspect of inhibitory synaptic transmission is the ability of gephyrin to build a scaffold by multimerization. To test whether the oligomeric state of gephyrin is dependent on cysteines and if those are redox-sensitive, we tested whether gephyrin presents redox-sensitive behavior in native tissue. Therefore, we perfused mice with N-ethyl-maleimide (NEM)-containing PBS to stabilize free thiols and avoid unspecific oxidation. In subsequent Western blot analysis, we either used the reductant dithiothreitol (DTT) or omitted it, allowing to compare the presence of redox-dependent gephyrin species (Fig. 1A). In brain lysates, the absence of a reductant resulted in bands corresponding to a molecular weight of approximately 300 kDa representing multimeric gephyrin species, whereas reduction of gephyrin led exclusively to monomeric gephyrin (100 kDa, Fig. 1A). The fractional abundance of multimeric gephyrin was approximately 20% of total gephyrin. Based on this finding, we hypothesized that gephyrin cysteines are redox-sensitive and that thiol oxidation modulates intermolecular interactions, thereby influencing gephyrin multimerization, which is a crucial parameter for its synaptic function^35^. Thus, we wondered which surface cysteines of gephyrin could be important for multimerization.

Based on crystal structures of G- and E-domain^36,37^, the identification of palmitoylation^26^, as well as the disordered nature of the C-domain^14,32^, we assigned cysteines at position 26, 154, 212, 284, 293, 419, 469, and 663 as being surface-exposed (Fig. 1B, C). Interestingly, those cysteine residues are conserved in orthologues harboring a central nervous system (Fig. S1), while other proteins, such as Cnx1 in plants or bacterial orthologues, lack those cysteine residues at their respective positions. To investigate the potential role of surface-exposed cysteines in redox regulation of gephyrin function, we generated cysteine variants by the substitution to serine (Fig. 1D). Previously, Cys212 and 284 were identified as targets of palmitoylation^26^. There, each of the other eight surface cysteines was individually substituted with serine^26^. Overall, except for the palmitoylated Cys212 and 284, none of the other substitutions resulted in any noticeable effect on gephyrin cluster size in neurons with endogenous gephyrin, which suggested that they were individually dispensable^26^. To test whether more than one cysteine outside the palmitoylation motif may have a functional role, we generated a gephyrin variant containing only Cys212 and 284 (o212/284) by replacing all other surface cysteines (Cys26, 154, 293, 419, 469, 663) with a serine. Additionally, we generated variants with remaining Cys212 (o212) and Cys284 (o284), respectively, and one variant without any surface-exposed cysteine, which we called cys-free. Importantly, in all variants, the four internal cysteines (Cys74, 133, 635, 676) remained unchanged.

The respective gephyrin variants were expressed and purified to homogeneity (Fig. S2A). All variants showed similar secondary structure distribution as judged by CD-spectroscopy (Fig. S2B). Besides that, we observed similar expression in COS7 cells (Fig. S3A, B) and functional enzymatic activity, with the exception of cys-free, which displayed ~60% enzymatic activity (Fig. S3C).

With the purified variants, we performed a redox-shift assay (Fig. 1E) to determine free thiols by the attachment of a maleimide conjugate with a PEG-tag (mPEG_5kDa_), which requires an accessible, free thiol. Each labelled cysteine increased the size of gephyrin by approximately 10 kDa. Note that internal Cys74, 133, 635, and 676 are accessible in the unfolded protein only (maximum shift). Thus, we confirmed that each individual cysteine among the eight predicted ones were indeed surface exposed, regardless of the presence of other cysteines (Fig. 1E).

**Fig. 2:**
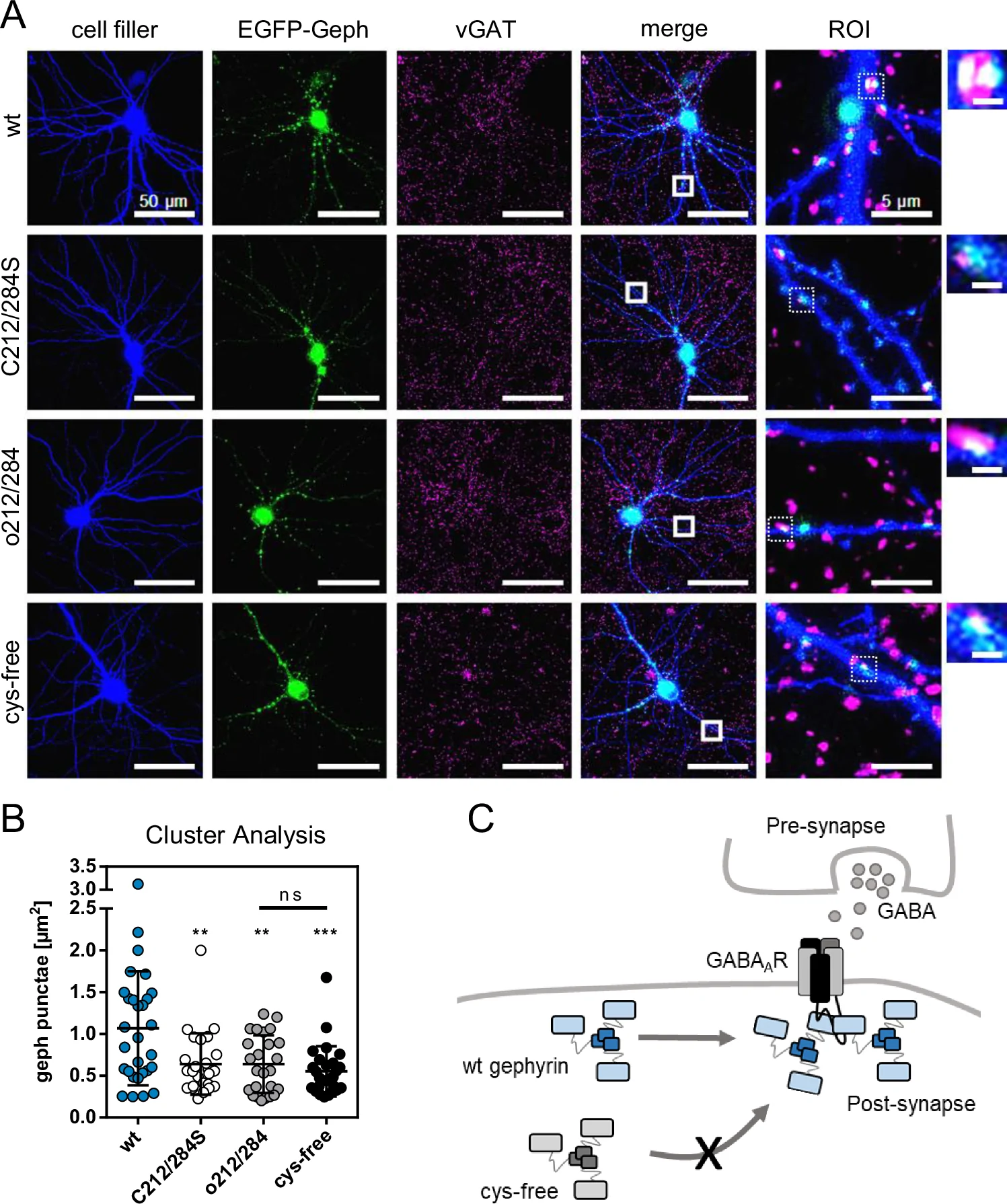
Gephyrin surface-exposed cysteines are important for synaptic clustering. **A** Primary, hippocampal neurons (DIV13) transfected with EGFP-gephyrin (green) and tdTomato (cell filler, blue) (scale bar 30 µm). Synaptic termini identified by vGAT staining. Dendrites enlarged (scalebar 5 µm) with increments of respective synaptic gephyrin clusters (scale bar 1 µm). **B** Cluster analysis of synaptic gephyrin. Mean (±SD). wt, *n* = 29 neurons, C212/284S, *n* = 26 neurons, o212/284, *n* = 25 neurons, cys-free, *n* = 27 neurons from 3 individual embryos of one neuron preparation. Significance tested by 1way ANOVA *F*(3,103) = 7.240, *p* = 0.0002. Bonferroni post-hoc test in comparison to wt: C212/284S: *p* = 0.0033 (**); o212/284: *p* = 0.0035 (**), cys-free: *p* = 0.0002 (***). Bonferroni post-hoc test of o212/284 and cys-free *p* = 0.9368 (ns).   **C** Scheme of gephyrin clustering at GABAergic synapses. Cys-free gephyrin shows disturbed synaptic clustering. Source data are provided as a Source Data file.

Based on the observed redox-dependent multimerization and redox-shift data, we anticipated a critical role for the eight surface-exposed cysteines in gephyrin. We focused on the scaffolding function of gephyrin in neurons. First, we performed Western blot and RT-PCR analysis demonstrating equal levels of expression for all variants (Fig. S4). Afterwards, we expressed EGFP-tagged wt gephyrin, the variant lacking the palmitoylation sites (C212/284S), and our newly generated variants (o212/284 and cys-free) in hippocampal neurons (Fig. 2A). Subsequently, we assessed synaptic gephyrin cluster size (Fig. 2B). Overall, the expression of EGFP-tagged wt gephyrin resulted in synaptic gephyrin puncta sizes with a high degree of variability, possibly due to a number of different PTMs that can regulate its clustering^18^. The o212/284 variant did not exhibit a wt-like phenotype. The disturbance of synaptic clustering was similar to that observed in the C212/284S variant, as the synaptic cluster size was significantly reduced. This finding led us to conclude that the other surface cysteines serve an unidentified function, distinct from that of palmitoylation^26^. Furthermore, this unknown function appears to be dependent on multiple cysteines, as single substitutions that were studied before did not show any significant impact on synaptic function^26^. Moreover, the cysteine-free variant was in comparison to wt gephyrin, significantly impaired, which was comparable to the o212/284 variant, underlying the importance of all surface-exposed cysteines.

In summary, our data demonstrate that at least eight cysteines in gephyrin are surface exposed and play an important role in synaptic clustering (Fig. 2C). Furthermore, the underlying mechanism responsible for the multimerization of gephyrin at synapses relies on the cooperative action of multiple cysteines, which is in line with the identification of redox-dependent multimers found in native tissue.

### Gephyrin undergoes reversible redox-dependent multimerization

**Fig. 3:**
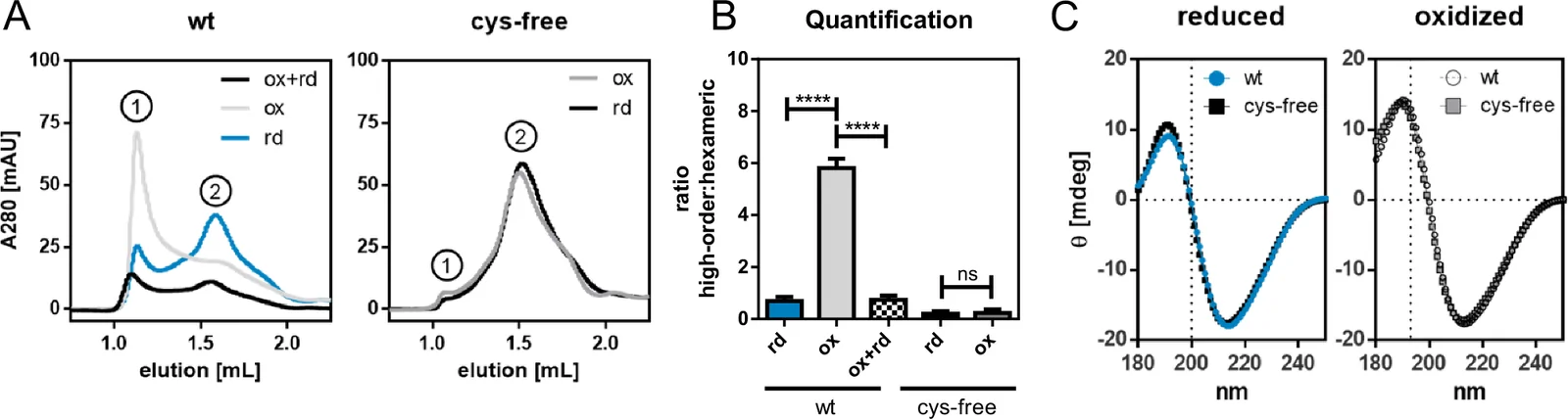
Reversible redox-dependent oligomerization of gephyrin in vitro. **A**, **B** Size exclusion chromatography (SEC) with oxidized or reduced gephyrin wildtype (wt) or cysteine-free (cys-free) variant. **A** Chromatograms with peaks of high-order multimers (1) or hexameric gephyrin (2). Reduced runs in blue (wt) or black (cys-free), oxidized in grey (wt and cys-free) and oxidized peak collected and subsequently re-reduced in black (wt). **B** Quantification of the ratio of the peak heights of three different runs. Mean (±SD). Significance tested by 1way ANOVA *F*(4,10) = 137.2, *p* = 1.08 × 10^−^^8^, Bonferroni post-hoc test: wt rd vs wt ox *p* = 7.39 × 10^−^^8^ (****); wt rd vs wt ox + rd *p* = 7.88 × 10^−^^8^ (****); cys-free rd vs cys-free ox *p* = 0.9108 (ns). **C** CD Spectroscopy. Spectra of oxidized or reduced gephyrin wt or cys-free variant. Vertical dotted line indicates HT threshold of 800 mV. Source data are provided as a Source Data file.

Given that cysteine residues contribute to the regulation of gephyrin density at inhibitory synapses, and based on our observation of higher oligomer gephyrin species in brain extracts, we initially hypothesized that oxidation-induced disulfide bond formation may regulate gephyrin multimerization. However, we were unable to detect covalent gephyrin multimers in neuronal or non-neuronal cell culture systems, including HepG2, and neuroblastoma cells (Fig. S5A–C). This prompted us to directly assess whether oxidative conditions per se can modulate gephyrin multimerization in a controlled in vitro setting. To this end, we treated recombinantly expressed gephyrin derived from *E. coli* with either the oxidant diamide or the reductant Tris-2-carboxyethyl-phosphine (TCEP) and analyzed the multimeric state of gephyrin using size exclusion chromatography (SEC). As a negative control, we used cysteine-free gephyrin. Upon oxidation, wt gephyrin displayed a pronounced shift toward higher-order species, consistent with an increased hydrodynamic radius and multimerization, whereas no such shift was observed for the cysteine-free variant (Fig. 3A, B). Importantly, treatment with the reducing agent TCEP reversed this effect, restoring the elution profile to its pre-oxidation state. Similar results were obtained when diamide was replaced with H₂O₂ and TCEP with DTT (Fig. S5D, E). Together, these findings demonstrate that oxidative conditions can reversibly promote gephyrin multimerization in a cysteine-dependent manner in vitro. While our data from brain tissue hinted towards the formation of disulfide-linked species under physiological conditions, the absence of detectable covalent multimers in cultured cells suggests that additional or alternative thiol-based modifications, such as sulfenylation or sulfinylation, may contribute to redox-dependent gephyrin organization in cells.

### Gephyrin oxidation increases liquid-liquid phase separation

We identified that gephyrin multimerization was reversible and inducible by oxidation in vitro and we observed redox-dependent multimers in vivo. Next, we asked whether these oxidized, multimerized gephyrin species retain their functionality. Given that multimerization is a crucial feature of gephyrin, we sought to investigate the molecular consequences with respect to its biochemical properties, such as structure and receptor binding.

Firstly, we examined the secondary structure and binding capability of both wt gephyrin and the cysteine-free variant under oxidative and reductive conditions. Overall, we observed no alterations in the secondary structure, regardless of whether all cysteines were replaced with serines and regardless of whether gephyrin underwent reduction or oxidation (Fig. 3C). Similarly, we found no changes in the binding properties of gephyrin towards the glycine receptor β-ICD using isothermal titration calorimetry (ITC) experiments (Fig. S6). For both wt and cys-free variant, the interaction with the βICD peptide occurred in a two-site binding mechanism^38^ (Fig. S6). The stoichiometry observed indicated approximately one gephyrin trimer interacting with one βICD peptide. The dissociation constant *K*_*D*_ was in the low micromolar range (wt = 0.041 ± 0.010 μM; cys-free = 0.063 ± 0.018 μM). Consequently, we concluded that cysteine-dependent oxidation does not alter the secondary structure or binding properties of gephyrin, while it changed its oligomeric state.

It would be plausible that oxidation-induced multimerization could lead to tighter packing of gephyrin clusters. Importantly, the quantity of gephyrin present at synapses and the protein density of clusters are crucial factors determining the number of receptor binding sites. Consequently, clustering can impact synaptic plasticity and neurotransmission. In line with this notion, we investigated in which manner oxidative processes would occur and be maintained, since gephyrin is a cytosolic protein and the cytosol represents a reductive environment. One possibility is the ability to form phase-separated droplets in the presence of a multimeric binding partner.

**Fig. 4:**
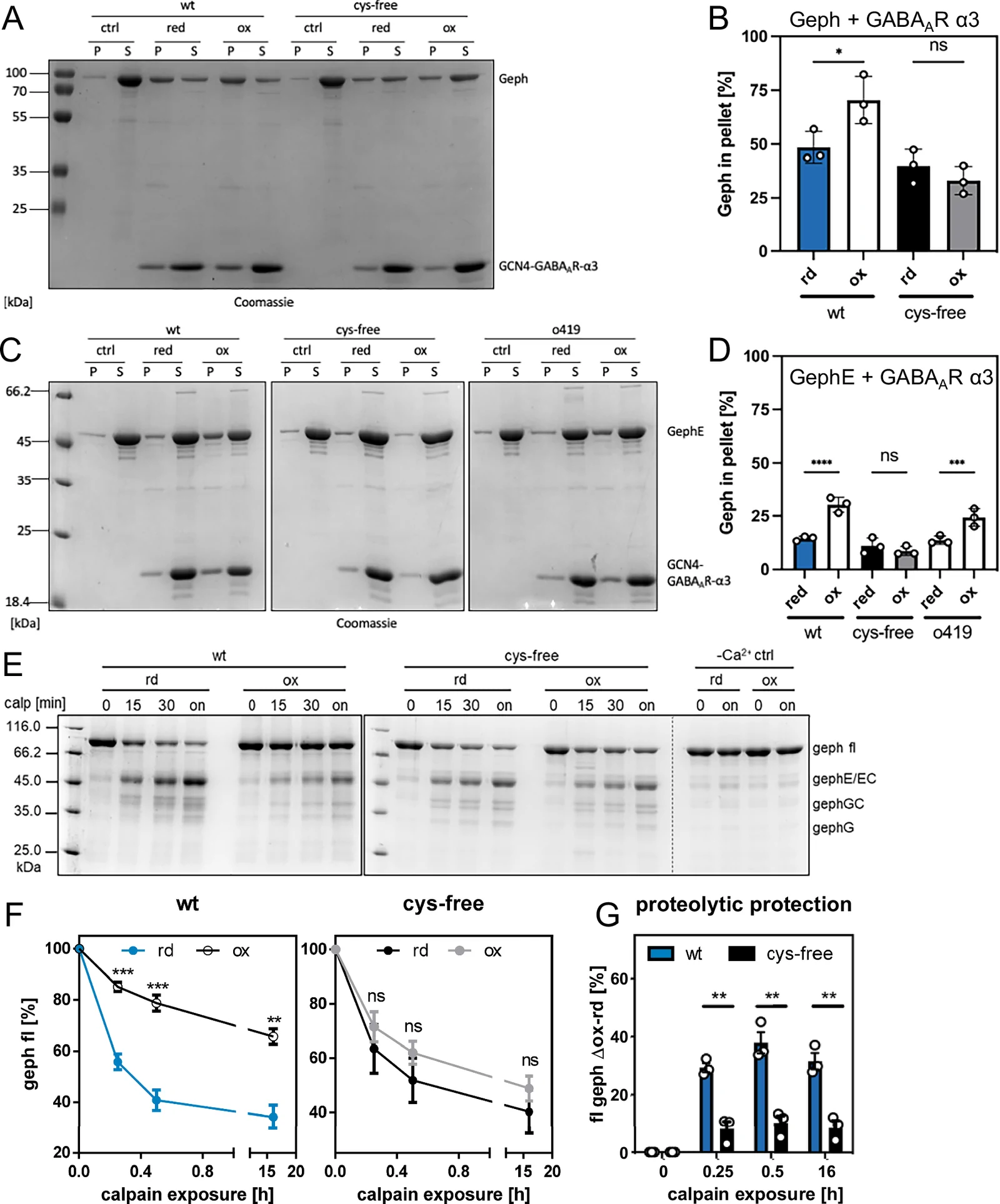
Sedimentation and calpain cleavage assays of oxidized and reduced gephyrin. Oxidized (ox) or reduced (red) full-length gephyrin and isolated E-domain (GephE) wt and cysteine-free were used for sedimentation (**A**–**D**) and calpain cleavage (**E**–**G**) assays. *n* = 3 independent experiments, mean and standard deviation are displayed. **A**, **B** Sedimentation assay using GCN4-GABA_A_R α3, condensation causes droplet formation (liquid-liquid phase separation), these droplets are sedimented by centrifugation. Sedimented proteins are recovered from the pellet (P), non-phase separated protein stays in the supernatant (S) fractions. As control oxidized gephyrin wt and cysteine-free alone were used. **A** Representative Coomassie Staining of fractions. **B** Quantification of gephyrin band intensities. Relative abundance (%) normalized to sum of protein in pellet and supernatant of the corresponding fractions. Significance tested by 2way ANOVA *F*(5,10) = 37.37, *p* = 3.72 × 10^−^^6^. Tukey’s multiple comparisons test: wt: *p* = 0.0357 (*); cys-free: *p* = 0.8538 > 0.05 (ns). **C**, **D** Similar experiment with GephE and GCN4-GABA_A_R α3. Significance tested by 2way ANOVA *F*(8,16) = 48.29, *p* = 9.27 × 10^−^^10^. Tukey’s multiple comparisons test: wt: *p* = 2.91 × 10^−^^5^ (****); cys-free: *p* = 0.8817 > 0.05 (ns); oC419: *p* = 0.0003 (***). **E**–**G** Exposure of gephyrin to the protease calpain for different times. **E** Representative Coomassie staining. Degradation products of gephyrin indicated at the different heights. fl full-length, gephE E-domain, gephEC E-domain with C-domain fragments, gephG G-domain, gephGC G-domain with C-domain fragments. In the control, calcium was absent. **F** Quantification of full-length (fl) gephyrin in each sample. Relative abundance (%) normalized to the start point 0. Significance between rd and ox determined by two-sided *t*-test and significance level alpha corrected by Bonferroni (/3): wt t1: *p* = 0.0001 (***); t2: *p* = 0.0002 (***); t3: *p* = 0.006 (**); cys-free: t1: *p* = 0.2535 (ns); t2: *p* = 0.1292 (ns); t3: *p* = 0.1843 (ns). **G** Comparison of proteolytic resistance. Relative abundance of fl geph in the reduced samples was subtracted from that of the oxidized ones (Δox-rd). Significance tested by two-sided *t*-test and significance level alpha Bonferroni corrected (/3): t1: *p* = 0.0022 (**); t2: *p* = 0.0031 (**); t3: *p* = 0.0033 (**). Source data are provided as a Source Data file.

To investigate LLPS of gephyrin in relation to its redox-state, we used the GCN4 receptor model for GlyR, GABA_A_R α1, and GABA_A_R α3^30^. While a substantial amount (~ 60%) of gephyrin was detected in the pellet fraction after co-incubation with GCN4-GlyR, no significant differences were observed when comparing oxidizing to reducing conditions (Fig. S7A). However, when gephyrin was incubated with either GCN4-GABA_A_R α3 (Fig. 4A, B) or GCN4-GABA_A_R α1 (Fig. S7B), significantly more gephyrin was detected within the respective pellet fractions under oxidizing compared to reducing conditions. Importantly, for all three model systems, gephyrin lacking surface-exposed cysteine residues did not show redox-sensitive behavior in LLPS (Figs. 4A, B and S7A, B).

Because our recent work has highlighted the importance of SDII–SDII′ interactions in driving gephyrin LLPS^33^, we next examined whether any surface-exposed cysteine residues might directly contribute to this interface. Notably, Cys419 is positioned on the surface of SDII (Fig. 1C). To test its functional relevance, we purified three different gephyrin E-domain variants (GephE),  wt, a cysteine-free variant (C419/469/663S; cys-free), and a variant retaining only Cys419 (o419), and subjected them to LLPS assays using the three GCN4 receptor model systems described above. Consistent with our results for full-length wt gephyrin, oxidation selectively enhanced phase separation of GephE in the presence of GCN4-GABA_A_R α3 (Fig. 4C, D) and GCN4-GABA_A_R α1 (Fig. S7D), but not with GCN4-GlyR (Fig. S7C). Importantly, cys-free GephE failed to undergo phase separation under all conditions, whereas reintroduction of Cys419 alone was sufficient to restore LLPS capability. This finding was further potentiated under oxidizing conditions, particularly in combination with GCN4-GABA_A_R α3 (Fig. 4C, D), suggesting that Cys419 plays a central role in mediating redox-sensitive modulation of SDII-driven gephyrin assembly.

Calpain-mediated cleavage is an important mechanism for the regulation of gephyrin clustering at synapses^15^. Besides calcium levels, the access to calpain cleavage sites determines the rate of proteolysis, which could be altered through redox-dependent multimerization. Therefore, the ability of calpain to cleave reduced or oxidized gephyrin was tested in vitro (Fig. 4E–G). The sensitivity of oxidized gephyrin to calpain cleavage was significantly reduced by approximately twofold, while there was no significant difference between reduced wt gephyrin and oxidized or reduced cysteine-free gephyrin (Fig. 4E, F). Analyzing the difference in remaining full-length, undigested gephyrin between oxidized and reduced protein (Δox-rd), showed that wt gephyrin exhibited significant proteolytic resistance after oxidation even after short exposure towards calpain (Fig. 4G). In contrast, the cysteine-free variant was equally digested as reduced wt gephyrin, regardless of whether it was in the reduced or oxidized state. In summary, our results show that structural properties of gephyrin that are influenced by its oligomeric state, such phase separation as well as proteolytic sensitivity, were redox-dependent.

### H_2_O_2_ mediates synaptic clustering of gephyrin in neurons

**Fig. 5:**
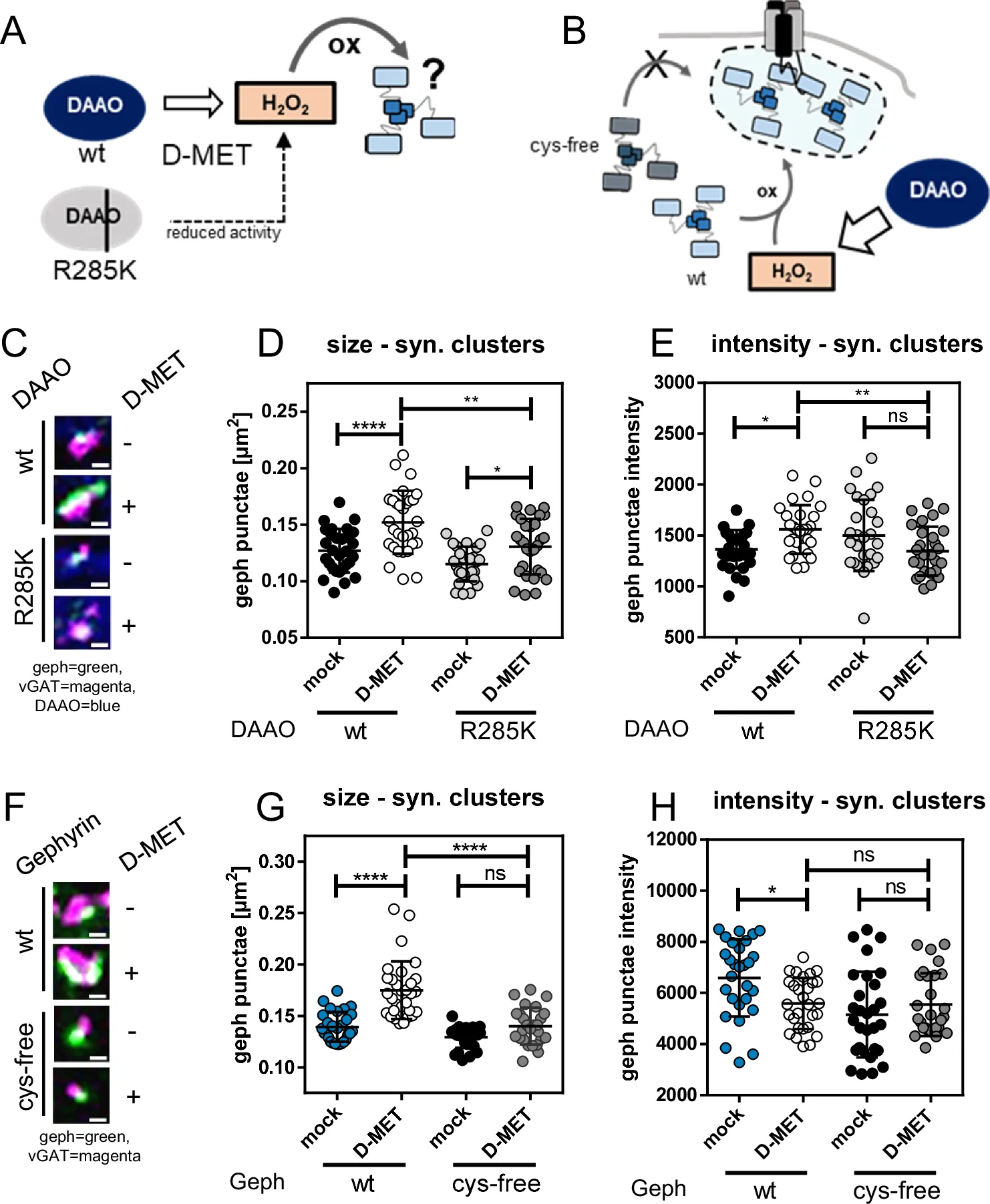
H_2_O_2_ drives gephyrin oligomerization at synapses. Expression of cytosolic mSclt-DAAO and mEGFP-Geph in primary hippocampal neurons. Treatment at DIV14 with 5 mM D-MET for 1 h. Analysis of cluster sizes [µm^2^], intensity [au] and co-localization with vGAT ( = synaptic clusters) of gephyrin. **A** Schematic description of H_2_O_2_ production of DAAO wt upon D-methionine acid treatment. DAAO mutant R285K is less active. Can H_2_O_2_ oxidize gephyrin and influence its oligomerization? **B** Schematic description of influence of DAAO-generated H_2_O_2_ on gephyrin clustering. Is cys-free gephyrin not influenced? **C**, **D** mSclt-DAAO wt and R285K. Analysis of endogenous gephyrin clusters. **C** Representative gephyrin clusters (Gephyrin: green, vGAT: magenta, DAAO: blue) (scale bar 0.5 µm). Synaptic termini identified by vGAT co-localization. **D** Sizes of synaptic gephyrin. DAAO wt mock, *n* = 29 neurons, DAAO wt D-MET, *n* = 30 neurons, DAAO R285K mock, *n* = 29 neurons, DAAO R285K D-MET, *n* = 29 neurons from 3 individual embryos of one neuron preparation. Mean (±SD). Significance tested by 1way ANOVA *F*(3,113) = 14.18, *p* = 6.58 × 10^−^^8^. Bonferroni post-hoc test: wt vs wt + D-MET: *p* = 9.47 × 10^−^^5^ (****); R285K vs R285K + D-MET: *p* = 0.0277 (*), wt + D-MET vs R285K + D-MET: *p* = 0.0010 (***). **E** Intensity of synaptic gephyrin. DAAO wt mock, *n* = 27 neurons, DAAO wt D-MET, *n* = 28 neurons, DAAO R285K mock, *n* = 29 neurons, DAAO R285K D-MET, *n* = 29 neurons from 3 individual embryos of one neuron preparation. Mean (±SD). Significance tested by 1way ANOVA *F*(3,109) = 4.431, *p* = 0.0056. Bonferroni post-hoc test: wt vs wt + D-MET: *p* = 0.0204 (*); R285K vs R285K + D-MET: *p* = 0.0808 (ns), wt + D-MET vs R285K + D-MET: *p* = 0.0083 (**). **F**–**H** mSclt-DAAO wt and mEGFP-Geph wt and cys-free variants. Analysis of recombinant mEGFP-gephyrin clusters. **F** Representative gephyrin clusters (Gephyrin: green, vGAT: magenta) (scale bar 0.5 µm). Synaptic termini identified by vGAT co-localization. **G** Sizes of synaptic gephyrin. Geph wt mock, *n* = 28 neurons, Geph wt D-MET, *n* = 30 neurons, Geph cys-free mock, *n* = 27 neurons, Geph cys-free D-MET, *n* = 26 neurons from 3 individual embryos of one neuron preparation. Mean (±SD). Significance tested by 1way ANOVA *F*(3,107) = 31.66, *p* = 9.95 × 10^−^^15^. Bonferroni post-hoc test: wt vs wt + D-MET: *p* = 4.59 × 10^−^^10^ (****); cys-free vs cys-free + D-MET: *p* = 0.1252 (ns), wt + D-MET vs cys-free + D-MET: *p* = 1.93 × 10^−^^9^ (****). **H** Intensity of synaptic gephyrin. Geph wt mock, *n* = 28 neurons, Geph wt D-MET, *n* = 29 neurons, Geph cys-free mock, *n* = 29 neurons, Geph cys-free D-MET, *n* = 28 neurons from 3 individual embryos of one neuron preparation. Mean (±SD). Significance tested by 1way ANOVA *F*(3110) = 5.608, *p* = 0.0013. Bonferroni post-hoc test: wt vs. wt + D-MET: *p* = 0.0212 (*); cys-free vs cys-free+D-MET: *p* = 0.8567 (ns), wt + D-MET vs cys-free + D-MET: *p* > 0.9999 (ns). Source data are provided as a Source Data file.

To address the question of how gephyrin wt in comparison to the cys-free variant could be oxidized in neurons, we investigated H_2_O_2_, which is a commonly known oxidant emerging from different sources in the cell. Therefore, we expressed a cytosolic version of a D-amino acid oxidase (DAAO) with an mScarlet-tag in mouse hippocampal neurons and tested the effect on endogenous gephyrin clusters or on recombinant cys-free and wt gephyrin (Fig. 5A, B). DAAO enzyme produces H_2_O_2_ by the conversion of D-amino acids to the corresponding α-keto acids (Fig. 5A), thus we treated our cells with D-Met. As a negative control, we generated a less active variant of DAAO by substituting Arg285 to Lys^39^. Following cell treatment, we stained for the presynaptic marker vGAT and analyzed its co-localization with endogenous gephyrin to identify synaptic gephyrin species.

Firstly, the ubiquitous and cytosolic localization of DAAO could be demonstrated (Fig. 5C, full images in Fig. S8A). D-methionine (D-Met) treatment caused a significant increase of synaptic cluster sizes by approximately 20% (from 0.1269 to 0.1521 µm^2^) (Fig. 5C, D). Additionally, the intensity of synaptic gephyrin was significantly increased by about 14% (from 1364 to 1559 a.u.) (Fig. 5E). The expression of DAAO R285K variant in combination with D-Met treatment resulted in a significantly increased gephyrin cluster size as well, but the effect size was smaller (Confidence Interval [−0.011; −0.039]^wt^, [−0.001; −0.030]^R285K^). The effect could be explained by the residual activity of the R285K mutant^39^. However, no effect of the treatment in combination with the R285K mutant on the intensity of gephyrin was observed. Based on this observation, we conclude that the effects on gephyrin resulted mainly from the enzymatic activity of DAAO, the production of H_2_O_2_. Since increase of cluster size and intensity are signs of gephyrin recruitment to the synapse, we concluded that H_2_O_2_ is able to trigger synaptic multimerization of gephyrin in neurons.

Next, we verified that H_2_O_2_ had a direct effect on the cysteines of gephyrin. Thus, we combined the expression of DAAO and subsequent D-Met treatment with the overexpression of mEGFP-tagged gephyrin (Fig. 5F, full images in Fig. S8B). As expected, overexpressed wt gephyrin showed a significant increase of about 25% (from 0.1393 to 0.1750 µm^2^) in cluster size following D-Met treatment, while cys-free gephyrin showed no effect (Fig. 5F, G). Strikingly, the intensity of synaptic mEGFP-gephyrin wt upon DAAO-mediated H_2_O_2_ production was decreased by −15% (from 6591 to 5588 a.u.), but that of cys-free gephyrin was unchanged (Fig. 5H). This effect may originate from cysteine-dependent oxidation and/or impacts on mEGFP folding in oxidized mEGFP-gephyrin wt clusters that may be shielded from the reductive cytosol^40^. In this set up, oxidation increased existing gephyrin cluster sizes but did not increase the density within clusters.

In conclusion, we could show that DAAO-mediated H_2_O_2_ promotes synaptic gephyrin cluster formation. The mechanism involves surface cysteines of gephyrin since cysteine-free gephyrin did not react to cellular H_2_O_2_ formation.

### Gephyrin oxidation promotes inhibitory synaptic transmission

To assess whether redox-dependent modulation of gephyrin clustering affects inhibitory synaptic transmission, we performed whole-cell patch clamp recordings to measure miniature inhibitory postsynaptic currents (mIPSCs). This approach was based on our biochemical findings indicating that gephyrin cysteine residues mediate redox-sensitive clustering, potentially influencing the availability of synaptic GABA_A_Rs.

We conducted two sets of experiments to analyze functional consequences at the synapse. First, we compared mIPSCs in wt neurons and neurons expressing the cys-free variant. Second, we manipulated intracellular ROS levels to modulate the redox state. To this end, neurons were transfected with either catalytically active DAAO and treated with D-Met to elevate ROS levels (wt DAAO + D-MET), or with a catalytically inactive DAAO mutant (R285K).

**Fig. 6:**
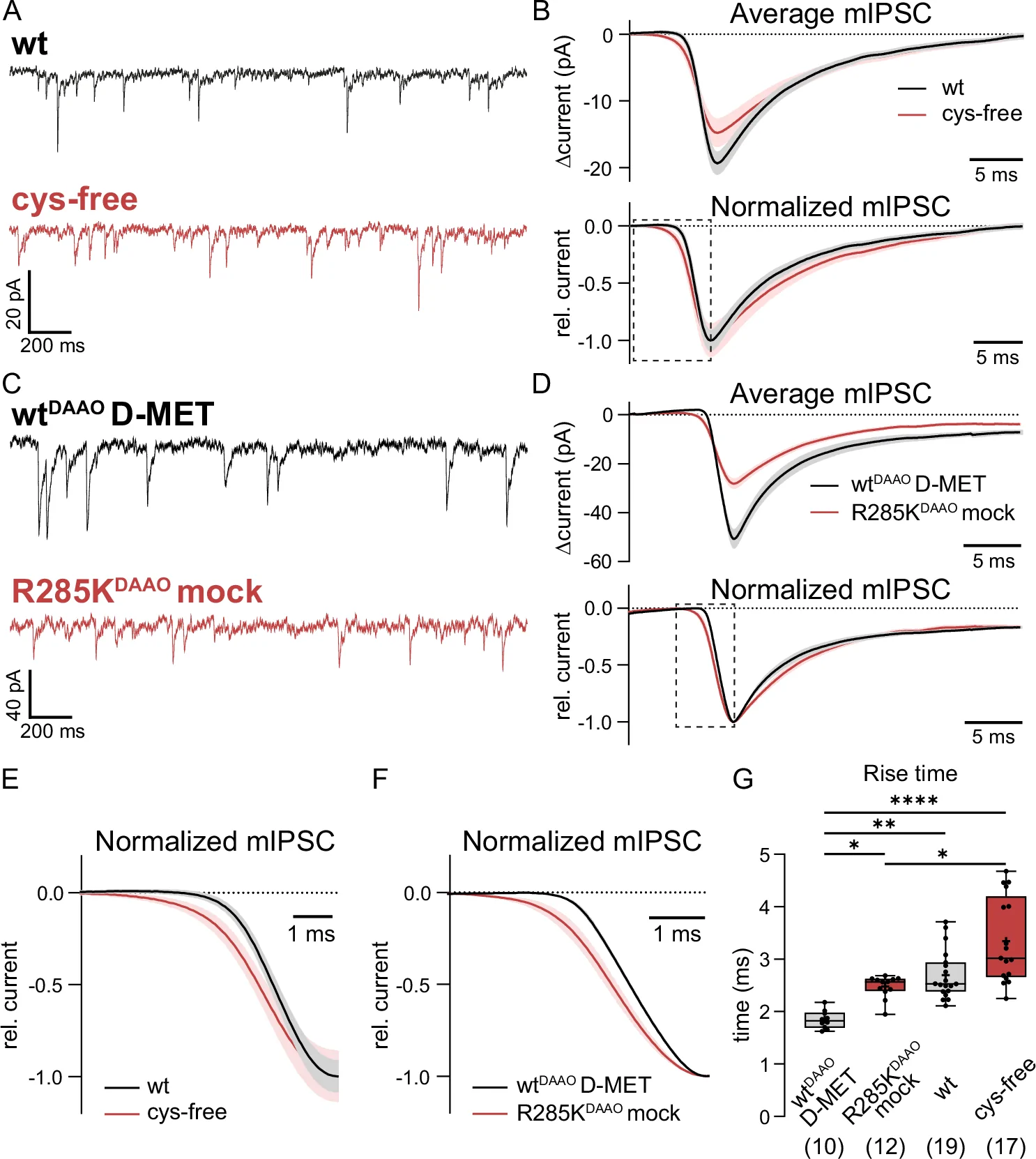
Gephyrin surface cysteines and intracellular H_2_O_2_ result in faster mIPSC rise times. Miniature inhibitory postsynaptic currents (mIPSCs) recorded in primary cultures of murine hippocampal neurons. **A**, **B** Neurons expressing mEGFP gephyrin wt or cys-free. **C**, **D** Neurons expressing wt DAAO and treated with D-MET (wt^DAAO^ D-MET), or expressing R285K DAAO and receiving mock treatment (R285K^DAAO^ mock). For the wtDAAO D-MET group, D-methionine (5 mM) was bath applied for at least 1 h before the start of the recordings. **A**, **C** Example mIPSC traces. **B**, **D** Mean ( ± SEM) mIPSCs (top) and normalized mIPSCs (bottom). Sample sizes: **B** wt, *n* = 20 neurons from embryos of 4 pregnant mice; cys-free, *n* = 17 neurons from embryos of 5 pregnant mice. **D** wt^DAAO^ D-MET, *n* = 10 neurons from embryos of 2 pregnant mice; R285K^DAAO^ mock, *n* = 12 neurons from embryos of 3 pregnant mice. **E**, **F** Expanded view of the rising phase (dashed boxes in (**B** and **D**)) of the normalized mIPSCs. **G** Box plots of mIPSC rise times for the indicated genotypes and treatment groups. Each data point represents the mean rise time of all mIPSCs recorded during a 50s interval from a single neuron (260 ± 17 individual mIPSCs per neuron). All recordings were performed at 13–15 DIV. Glutamatergic transmission was blocked with CNQX and DL-AP5, and action potentials were blocked with TTX. Cells were voltage-clamped at −70 mV. In box plots, the center line indicates the median, the “+“ symbol the mean, the boxes the interquartile range (25th–75th percentile), and the whiskers the minimum and maximum values. Exact *p*-values are reported; significance is indicated as * *p* < 0.05, ** *p* < 0.01, *** *p* < 0.001, and **** *p* < 0.0001. Data were analyzed using a Kruskal–Wallis test (*H*(3) = 33.08, *p* = 3.10 × 10^−7^), followed by Dunn’s multiple-comparisons test: wt vs cys-free, *p* = 0.0808; wt vs wt^DAAO^ D-MET, *p* = 0.0012; wt vs R285K^DAAO^ mock, *p* > 0.9999; cys-free vs wt^DAAO^ D-MET, *p* = 6.63 × 10^−8^; cys-free vs R285K^DAAO^ mock, *p* = 0.0328; wt^DAAO^ D-MET vs R285K^DAAO^ mock, *p* = 0.0245. Sample sizes in (**G**): wt, *n* = 19 neurons from embryos of 4 pregnant mice; cys-free, *n* = 17 neurons from embryos of 5 pregnant mice; wt^DAAO^ D-MET, *n* = 10 neurons from embryos of 2 pregnant mice; R285K^DAAO^ mock, *n* = 12 neurons from embryos of 3 pregnant mice. Source data are provided as a Source Data file.

Both experimental manipulations, gephyrin cysteine deletion and ROS level alteration, resulted in significant changes in mIPSC properties (Fig. 6 and Supplementary Fig. S9). Particularly striking was the consistent and robust effect on mIPSC rise times observed across all experimental conditions. Specifically, mIPSC rise times were slower in neurons lacking gephyrin cysteine residues and faster in neurons with elevated ROS levels. The fastest rise times occurred in DAAO-overexpressing neurons, followed by R285K-expressing neurons (Fig. 6G). WT neurons exhibited intermediate rise times, whereas neurons expressing the cysteine-deficient gephyrin displayed the slowest rise times.

These findings align with our biochemical data demonstrating that gephyrin multimerization is modulated by redox state, at least in part through redox reactions involving its cysteine residues. The graded, statistically significant distribution of mIPSC rise times supports a model in which both intact gephyrin cysteine residues and increased intracellular oxidation promote faster inhibitory synaptic responses. Overall, this relationship between redox state, gephyrin cysteine content, and mIPSC kinetics supports our hypothesis that gephyrin cysteines act as redox-sensitive switches that regulate synaptic localization and clustering, thereby modulating the functional efficacy of inhibitory synaptic transmission.

## Discussion

**Fig. 7:**
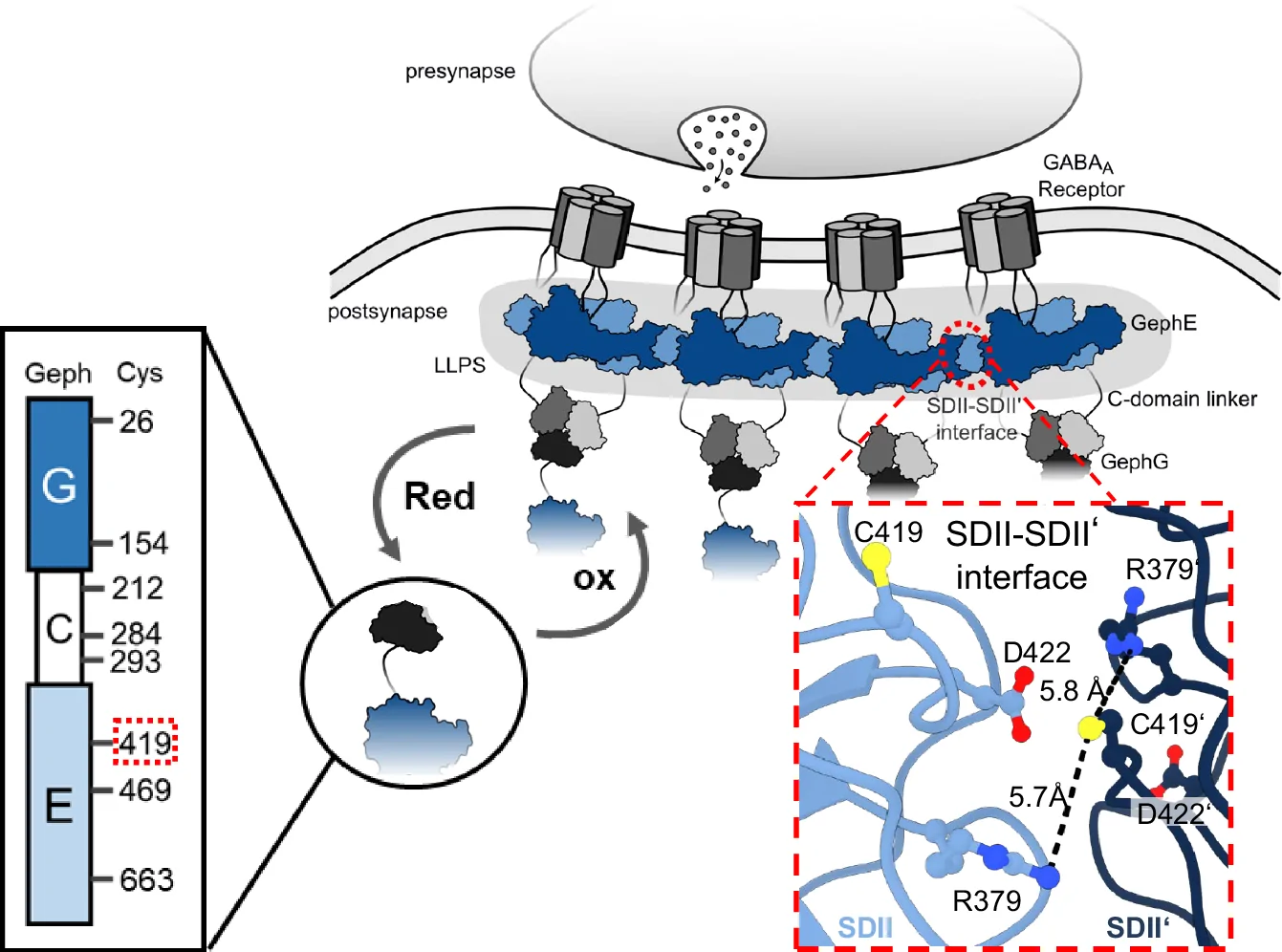
Redox-regulation of synaptic gephyrin clustering. Hypothesized model of redox-dependent gephyrin oligomerization at inhibitory GABAergic post-synapses. Gephyrin harbors eight surface-exposed cysteines that are redox-sensitive. ROS-mediated oxidation increases gephyrin oligomerization. This oxidation is reversible by reduction and allows for dynamic shuttling of gephyrin localization. LLPS (highlighted in grey) might offer an oxidative microdomain facilitating oxidation. Gephyrin oligomerization at synapses offers binding sites for GABA_A_Rs, ultimately strengthening inhibitory signal transmission.

Our study provides so far undefined insight into the mechanism of gephyrin-dependent inhibitory synapse formation. First, we identified surface-exposed cysteines in vitro and demonstrated their sensitivity to redox changes. Subsequently, we found that the multimerization of gephyrin was regulated by oxidation and reduction. We identified redox-dependent multimers in vivo and induced synaptic multimerization in cellulo through DAAO-derived H_2_O_2_. Oligomerization is a crucial aspect of gephyrin scaffolds, significantly impacting the plasticity of inhibitory synapses^35,41^. Consequently, the oligomeric state influences the number of available receptor binding sites and the sensitivity of gephyrin to proteolytic cleavage^17^. The structural basis of this oligomerization has only recently been resolved at high resolution by our  recent study, revealing gephyrin E-domain filaments stabilized by SDII–SDII’ contacts as the fundamental architecture driving both scaffold assembly and phase separation at inhibitory synapses^33^. Changing the oligomeric state by cysteine oxidation enhanced clustering, while reduction reversed this process (Fig. 7). In aggregate, our study provides a so far undefined role of surface-exposed cysteines in gephyrin, which were found to be conserved in orthologues with a central nervous system, while other orthologs in plants, yeast, fungi and prokaryotes lack those residues (Fig. S1).

Comparing our data for redox-dependent multimerization of gephyrin with PSD95, the functional pendant at the excitatory PSD, multimerization by the formation of disulfide bridges was detected in cellulo and in vivo^42^. Recently, the disruption of a cysteine linkage in PSD95 has been linked to an epileptic phenotype in rats^43^, highlighting the importance of redox-regulation of neuronal scaffold proteins.

Cysteine oxidation in gephyrin could be reversed by reduction, which is essential for enabling dynamic changes and maintaining plasticity. Importantly, cysteine oxidation did not influence the secondary structure of gephyrin and its binding to the receptor, but it altered the density of gephyrin at the synapse, manipulating the availability of receptor binding sites. One possible explanation for the strong cysteine-dependent effects in the absence of detectable disulfide-linked multimers in cells is a local electrostatic mechanism involving Cys419. In the SDII–SDII′ interface, the proximity of Arg379 may lower the pK_a_ of Cys419 (Fig. 7), promoting formation of a negatively charged thiolate. This thiolate could stabilize the interface by strengthening ionic interactions with Arg379, analogous to the established Asp422–Arg379 interaction critical for LLPS^33^. Furthermore, higher oxidation states of Cys419 would introduce additional negative charge, potentially further enhancing interface stability and thereby promoting gephyrin multimerization and phase separation without requiring covalent crosslinking via disulfide-bridge formation. However, to which extend such a modification may be formed in brain tissue over time requires future studies.

Although redox-dependent regulation of synaptic proteins has previously been investigated, important questions remain open, such as the source of oxidation. Given that neurons are rich in mitochondria and mitochondria are located in close proximity to the synapse^44^, we propose that mitochondria-derived ROS (mROS) could serve as a source for oxidation. In this sense, gephyrin could provide the functional link between mitochondrial and neuronal activity. Highly active neurons require ATP production mainly derived from mitochondrial respiration being accompanied by the formation of mROS, which is rapidly converted to H_2_O_2_^1,45,46^. H_2_O_2_ can reach the synapse and trigger gephyrin recruitment and network expansion, thereby recruiting additional receptors to further increase neurotransmission. Mitochondria are located in both pre-synaptic and post-synaptic regions, so it is conceivable that mROS could regulate the post-synapse from various angles and in reverse, it can impact both sides of the synapse.

Besides mitochondria, there are additional origins of H_2_O_2_, such as the NADPH-oxidase 2 (NOX2) pathway^47^. NOX2 is highly abundant in cells throughout the CNS^48^ and produces superoxide radicals in the extracellular environment, which are subsequently converted to H_2_O_2_. This H_2_O_2_ can re-enter the cell through aquaporins. In a recent study, the effects of this pathway on inhibitory neurotransmission were investigated^4^. It was shown that excitatory and inhibitory neurotransmission is mediated by ROS and nitric oxide, leading to the recruitment of GABA_A_R-containing α3 subunits at inhibitory synapses. In contrast to our study, they identified the GABA_A_R-associated protein (GABARAP) as the primary mediator of receptor recruitment, a protein that has been found to interact with gephyrin^49^. Although it is unclear whether different ROS-mediated signaling mechanisms can co-exist, it highlights the importance of these mechanisms in regulating neuronal plasticity. It is important to note that the effects of ROS-mediated signaling on glycinergic synapses has not been studied. Furthermore, we did not answer the question whether gephyrin was directly oxidized by H_2_O_2_ or other oxidant species, which could be derived from H_2_O_2_.

Another open question concerns the maintenance of oxidation processes in the reductive cytosol. In recent studies, LLPS has gained attention as a new principle of subcellular organization^50^. For gephyrin, it was recently demonstrated that it forms liquid droplets in the presence of a multimeric receptor model^30^. Importantly, our data showed that oxidation of gephyrin strengthened LLPS with GABA_A_R α1 and α3 but not with GlyR β model proteins. Neuroligin 2 (NLGN2) is a post-synaptic cell-adhesion molecule that can interact with pre-synaptic Neurexins and is exclusively found at GABAergic synapses^51^. Local NLGN2 accumulation can promote the nucleation of gephyrin condensates^32^. Our data demonstrate that gephyrin oxidation increased condensation with GABA_A_R model proteins, and future work could test whether oxidation could influence gephyrin nucleation (for example locally at NLGN2 contact sites). While our current study suggests that existing GABAergic synapses are strengthened by this process, work on gephyrin nucleation could be instructive as to whether the formation of new synapses could be promoted.

Prior studies investigated the relationship between the redox state of cysteines and the ability to undergo LLPS. One example is synapsin 1, which harbors two cysteines directly affecting LLPS in a redox-dependent manner, which is also relevant in aging^52^. A study, that is mechanistically comparable to our work, was reported for oleosin, where the disulfide-bridge formation of artificially added cysteine residues on the protein surface triggered LLPS, while reduction was able to reverse this effect^53^. In general, the concept of a reversible formation of phase-separated microdomains holds great potential and may be a key to understand redox-dependent processes in different cellular environments.

Building on the emerging view that SDII–SDII′ interactions constitute a filamentous structural element underlying gephyrin assembly and LLPS^33^, our observations further refine how this interface may be regulated at the molecular level. In this context, the positioning of Cys419 directly within the SDII surface suggests a mechanism by which redox-dependent modifications can influence intermolecular connectivity within gephyrin filaments and thus the entire network. Our LLPS data support this notion, as oxidation only in presence of Cys419 in combination with GABA_A_R α1 and α3 model systems selectively enhanced phase separation, whereas no additional effect was observed with GlyR β, which binds with much higher affinity to gephyrin. Mechanistically, these findings suggest that oxidative modifications at Cys419 may directly tune the strength of the SDII–SDII′ interface, thereby modulating the overall connectivity of the gephyrin network. In particular, reversible oxidation of Cys419 could introduce additional negative charge, potentially reinforcing the proposed salt bridge between D422 and the opposing R379 across the SDII–SDII′ interface^33^.

A limitation of our study is the use of dissociated hippocampal cultures, which do not maintain their correct anatomic input-specificity. While prior studies have demonstrated that inhibitory synapses in molecular layer interneurons are strengthened upon ROS-induction through the recruitment of α3-containing GABA_A_Rs in a gephyrin-independent manner^3,4^, our findings in hippocampal neurons suggest the existence of an additional gephyrin-dependent mechanism. Interestingly, in this mechanism, oxidation resulted in fast mIPSC rise times, which may reflect increased temporal precision of synaptic inhibition. Additionally, gephyrin oxidation in LLPS experiments suggests that both α1- and α3-containing GABA_A_Rs may be recruited by this mechanism.

Oligomerization as a function of the redox state of cysteines (including nitrosylation, persulfidation, sulfenylation and disulfide-formation) has been investigated previously^1,52,54^. Such modifications may lead to functional multimers or harmful aggregates depending on the site and degree of oxidation, thus reflecting the dose of ROS^8^. Imbalance in redox signaling is linked to aging and neurodegenerative diseases^7^. Our study identifies gephyrin as mediator between metabolic activity and inhibitory synapses using ROS as signaling mechanism.

## Methods

### Ethical approval

We complied with all relevant ethical regulations for animal testing and research, and experiments were approved by local ethics committees (Germany, Landesamt für Natur, Umwelt und Verbraucherschutz Nordrhein-Westfalen, reference 2019.A077, reference 2021.A450, reference 2024-561). The study is reported in accordance with the ARRIVE guidelines.

### DNA/expression constructs

Gephyrin P1 and cysteine variants in pQE80L (Qiagen) were cloned by Gibson assembly using EcoRI into pAAV_hSyn_mScarlet (a gift from Karl Deisseroth, Addgene plasmid #131001) and using EcoRI into pAAV_hSyn_mEGFP (generated from moxBFP, a gift from Erik Snapp, Addgene plasmid #68064; http://n2t.net/addgene:68064; RRID: Addgene_68064). Gephyrin was cloned using PCR and XhoI and KpnI into pEGFP-C2 (Clonetech). The template for the generation of rat Gephyrin P1 constructs was in pQE80L^55^. The cysteine to serine substitutions were generated by site-directed mutagenesis. DAAO was cloned into pAAV_hSyn_mScarlet through PCR and Gibson Assembly using EcoRV and the R285K mutation was introduced by site-directed mutagenesis. The plasmids pAdDeltaF6 (a gift from James M. Wilson, Addgene plasmid #112867; http://n2t.net/addgene:112867; RRID: Addgene_112867) and pAAV2/1 (a gift from James M. Wilson, Addgene plasmid #112862; http://n2t.net/addgene:112862; RRID: Addgene_112862) were used for virus production. The expression constructs of GlyRβICD in ptYB was described previously^56^. GCN4 constructs with GlyR, GABA_A_Rα1, and GABA_A_Rα3 were a kind gift from the laboratory of Prof. Mingjie Zhang, Shenzhen Southern University of Science and Technology^30^.

### Recombinant expression and purification of gephyrin

*E. coli* BL21 Rosetta star was used for the expression of His-gephyrin and His-gephE. Cultures were grown at 37 °C at 110 rpm and the expression induced at an OD_600_ of 0.3 with 100 µM IPTG. After expression for 22 h at 18 °C and 110 rpm, cells were harvested. To achieve reduced cysteines (free thiols), all solutions were supplemented with reductant until final storage of isolated proteins. The cell pellet was resuspended in lysis buffer (300 mM NaCl, 50 mM Tris/HCl, 5 mM beta-mercaptoethanol, 0.05% Tween20, 1 µg/mL lysozyme, 1× PI (*Roche*), pH 7.4) and stored at −80 °C. Pellets were thawed in a water bath and all steps performed at 4 °C. Cells were lysed through alternating ultrasound (3 min at 4 °C, 30 s pulse, 30 s pulse, 40% amplitude) and pressure lysis (*EmulsiFlex*, 1000–1500 bar, 4 °C) twice. The homogenate was centrifuged (45 min, 38,000 × *g*, 4 °C, JLA 16.250 rotor, Beckman-Coulter Centrifuge Avanti J25-01) and the supernatant subjected to Ni-NTA affinity purification (HIS-Select Nickel Affinity Gel (Sigma Aldrich)). All buffers for washing and elution were similar to the lysis buffer, contained no lysozyme, but contained 20, 40, and 300 mM Imidazole, respectively. The eluate was further subjected to preparative size-exclusion chromatography (Superdex 200, 120 mL, *GE Healthcare*) using SEC buffer (300 mM NaCl, 50 mM Tris/HCl, 5 mM beta-mercapto ethanol, 0.05% Tween20, 5% glycerol, pH 7.4). The fractions of the peaks eluting around 60–70 mL (Trimers) and 45–60 mL (higher multimers) were collected and concentrated by an *Amicon* filter with a Cut-off of 50 kDa. To remove reductants, the concentrated protein was buffer exchanged by gel filtration (PD10 column) to storage buffer (300 mM NaCl, 50 mM Tris/HCl, pH 7.4) and stored at −80 °C.

### Purification of βICD peptide from E. coli

For the βICD peptide, we used *E. coli* ER2566. Cultures grew at 37 °C and 110 rpm until the OD600 reached 0.3. Induction was induced by 250 µM IPTG and expression performed for 20 h at 25 °C and 110 rpm. Cells were harvested and stored at −80 °C in ICD lysis buffer (300 mM NaCl, 50 mM Tris/HCl, 1 mM EDTA, 1× Protease Inhibitor (*Roche*), pH 7.4). Lysis of the thawed cells was performed by alternation of ultrasound (3 min at 4 °C, 30 s pulse, 30 s pulse, 40% amplitude) and pressure lysis (*EmulsiFlex*, 1000–1500 bar, 4 °C) twice. After centrifugation for 1 h at 16,000 × *g* (JLA 16.25 rotor) at 4 °C, the supernatant was applied on a pre-equilibrated chitin matrix (*New England Biolabs*) and incubated 1.5 h at 4 °C under shaking. The column was washed with 15 CV ICD buffer before 2.5 CV cleavage buffer (300 mM NaCl, 50 mM Tris/HCl, 1 mM EDTA, 50 mM DTT, pH 7.5) was applied an incubated for minimum 24 h at rt. The eluate was collected and another 2.5 CV cleavage buffer used for a second elution. The combined eluate was concentrated and filtered by *Amicon* filters with a cut-off of 10 and 3 kDa. The buffer was exchanged to ITC buffer (300 mM NaCl, 50 mM Tris/HCl, pH 7.4) by dialysis over night at 4 °C. The purified protein was stored at −80 °C.

### GlyR-βLD, GABA_A_R-αLD and GABA_A_R-α3LD purification

GlyR-βLD, GABA_A_R-αLD and GABA_A_R-α3LD were purified according to the protocol provided by ref. ^30^. Recombinant proteins were expressed in *Escherichia coli* BL21-CodonPlus (DE3)-RL cells (Agilent Technologies) in LB medium at 16 °C overnight and protein expression was induced by 0.25 mM IPTG at OD600 between 0.6 and 0.8. Cultured cells were resuspended with the lysis buffer containing 50 mM Tris, pH 8.2, 1000 mM NaCl, 20 mM Imidazole, 1 mM PMSF and lysed with high pressure (*EmulsiFlex*, 1000–1500 bar, 4 °C). Cell lysis was clarified by 38,000 × *g* centrifugation for 20 min. Lysis supernatant was incubated with Ni^2+^-NTA affinity beads for 10 min at room temperature. The beads were washed with the 10× volume of lysis buffer three times. The proteins were eluted with the elution buffer containing 50 mM Tris, pH 8.2, 500 mM NaCl, 250 mM Imidazole. Eluted proteins were purified using size-exclusion column (Superdex 200) in buffer containing 20 mM HEPES, pH 7.4, 200 mM NaCl (for GlyR-βLD) or 500 mM NaCl (for GABAAR-αLD), 1 mM EDTA, 2 mM DTT. After the cleavage of the affinity tag by HRV 3 C protease (overnight at 4 °C), protein samples were diluted to around 100 mM NaCl and loaded to the ResourceS column to separate the remaining contaminating proteins and the cleaved affinity tag. Proteins eluted from ResourceS were exchanged to the final buffer containing 50 mM Tris, 100 mM NaCl, 5 mM DTT or 1 mM TCEP, pH 7.5 using desalting column. Final proteins were concentrated to around 200 μM and stored as aliquots at −80 °C.

### Redox shift assay

Typically, 10 μg protein were prepared in a total volume of 20 μL RSA buffer (50 mM Tris/HCl, 5 mM EDTA, pH 7.5) or denaturing RSA buffer (50 mM Tris/HCl, 5 mM EDTA, 2% SDS pH 7.5) and supplemented with 1 mM mPEG5kDa or buffer as minimum controls. After incubation for 1 h in the dark at rt, samples were used for SDS PAGE. The expected shift size per labeled thiol is about 10 kDa. In denaturing conditions, internal cysteines are exposed to PEGylation additionally.

### In vitro redox-modification

Purified proteins were oxidized by incubation overnight at 4 °C with 1 mM Diamide or H_2_O_2_. Reduction was performed with 1 mM TCEP or DTT. In case the reagent would disturb in further assays, the chemical was removed by gel filtration using PD10 columns.

### Sedimentation assay of in vitro phase separation

Prior to the assays, proteins were pre-cleared at 17,000 × *g* for 10 min at 25 °C. For sedimentation assays, proteins were mixed at designated combinations and conditions in 50 μL final volume in assay buffer (25 mM Tris pH 7.4, 150 mM NaCl). After 10 min incubation at 25 °C, the mixtures were centrifuged at 17,000 × *g* for 10 min at 4 °C. Right after centrifugation, the supernatant and pellet were separated. The pellet was brought to the same volume as the supernatant. Proteins recovered in supernatant and pellet were analyzed by SDS-PAGE with Coomassie Blue staining. The band intensities in SDS-PAGE gels were quantified by Image Lab software. Relative and absolute amounts of proteins in supernatant and pellet were calculated based on the relative band intensities. Data of three repeats were presented as means ± SD.

### ITC

Isothermal titration Calorimetry (ITC) experiments were performed by using MicroCal Auto-ITC200 (*Malvern*) at 25 °C in ITC buffer (300 mM NaCl, 50 mM Tris/HCl, pH 7.4). We used an injection volume of 1–2 μL, a spacing of 150 s between injections and an initial delay of 60 s. The reference power was 5 μCal/s and the stirring speed 1000 rpm. The concentration of GlyRβICD in the syringe was about 300–310 μM, and the concentration of gephyrin in the sample cell was 31–35 μM.

Parameters and curves were fitted and calculated by the software MicroCal Analysis and Origin7.

### Size exclusion chromatography

Molecular masses of purified gephyrin and variants were estimated by analytical size exclusion chromatography (SEC) using a Superose 6 increase 5/10 (*GE Healthcare*). Typically, 4 nmol pre-cleared protein in 50 μL total sample volume were applied. Proteins were separated at 4 °C and a flow rate of 0.1 mL/min in SEC buffer (50 mM Tris/HCl, 300 mM NaCl pH 7.4). Selected fractions of 100 µL were collected and those of corresponding peaks combined and concentrated using *Amicon* filters with a 50 kDa cut-off. The combined protein was then further subjected to redox-modifications, cleared by centrifugation and re-applied on the column.

### Calpain cleavage assay

A mastermix of 50 μL contained 850 pmol redox-modified protein, 10 mM Ca^2+^Cl_2_, 1/2 U/mL calpain. The volume was adjusted using calpain buffer (300 mM NaCl, 50 mM Tris/HCl, pH 7.4). As a control, a calcium-free control was included because calpain is a calcium-dependent protease. At timepoints of 0, 5, 15, 30 min and overnight, 15 µL samples were taken out and the digestion stopped by addition of SDS sample buffer and incubation for 5 min at 96 °C. Cleavage products were afterwards analyzed by SDS-PAGE.

### Circular dichroism spectroscopy

Determination of the secondary structure was performed by circular dichroism spectroscopy (CD spec). Typically, 0.3 mg/mL pre-cleared protein in CD buffer (10 mM K_2_HPO_4_/KH_2_PO_4_, pH 7.5) was used and Far-UV spectra recorded with J-715 CD spectropolarimeter (*Jasco*) at 20 °C in the range of 180–250 nm using quartz cuvette with 0.1 cm path length. The spectra were processed by the software Spectra Analysis version 1.53.07 of Spectra Manage version 1.54.0. We subtracted the buffer baseline and smoothed three-times by the Savitzky-Golay algorithm with a convolution width of 21.

### Virus preparation

Recombinant virus particles (AAV2/1) were produced in HEK293 cells and isolated by PEG/NaCl precipitation followed by chloroform extraction according to a previously established protocol^57^. The titers were determined by a Gel green® (*Biotium*) protocol^58^ and the fluorescence was detected (507 ± 5 nm excitation and 528 ± 5 nm emission) using a plate reader equipped with monochromators (Tecan Spark).

### Culture of hippocampal neurons and expression of recombinant gephyrin

Mice were housed on a 12 h light-dark cycle with free access to food and water at 22 ± 2 °C and a humidity of 55 ± 10%. At E17.5, dissociated primary hippocampal cultures were prepared from C57BL/6NRj embryos of either sex. 90,000 cells were seeded on Poly-L-lysine coated 13-mm cover slips in 24-well plates. Neurons grew in neurobasal medium supplemented with B-27, N-2, and L-glutamine (*Thermo Fisher Scientific*). At 8 days in vitro (DIV), cells were transfected with 0.4 µg plasmid DNA per construct using lipofectamine 2000 (Life Technologies) according to the manufacturer’s manual. Expression was performed for 5 days. Alternatively, at DIV10, cells were transduced with 2.5 × 10^8^ viral genome copies (GC). The virus particles were directly added to each individual well. At DIV14, the cells were either fixed or subjected to treatment. D-Methionine (D-Met) treatment was performed 1 h at 37 °C. The medium of neurons was removed and replaced by minimal NB medium, supplemented with 5 mM D-Met. Mock samples were treated with the same volume of solvent solution (PBS). After treatments neurons were fixed and used for ICC.

### ICC

Fixation was performed by replacing culture medium by 4% PFA in PBS. After incubation for 20 min at room temperature (rt), PFA was removed and cells washed twice with 50 mM NH_4_Cl in PBS, each time 10 min at rt. Subsequently, cells were blocked for 1 h at rt with blocking solution (10% goat serum, 1% BSA, 0.2% TritonX100, in PBS). After washing cells with PBS for 5 min at rt, antibody incubation was performed for 1 h at rt. Antibodies were diluted in PBS. Afterwards, three washing steps with PBS were performed, each 10 min at rt. Lastly, coverslips were mounted with Mowiol/Dabco and dried over night at rt. We used the following antibodies: anti-vesicular GABA transporter (vGAT) (1:1000, #131003, *SYSY*); anti-GABA_A_Rγ2 (1:500, #224004, *SYSY*); anti-GephyrinE (3B11; 1:10, self-made); goat anti-mouse AlexaFluor 488 (1:500, #A-11029, *Invitrogen*), goat anti-rabbit AlexaFluor 647 (1:500, #A-21245, *Invitrogen*), goat anti-rabbit AlexaFluor 488 (1:500, #A-11075, *Invitrogen*).

### Confocal microscopy and image analysis

Images were taken using a *Leica* TCS SP8 LIGHNING upright confocal microscope with an HC PL APO CS2 63×/1.30 glycerol objective. The microscope was equipped with hybrid *Leica* HyD detectors and diode lasers with 405, 488, 522, and 638 nm. The LIGHTNING adaptive deconvolution of the mounting medium Mowiol was used, which is capable of theoretical resolutions down to 120 and 200 nm lateral and axial, respectively. Images were acquired with stacks of 3 µm z-step size, 2048 × 2048 (144.77 × 144.77 µm) and segmented and analyzed in an automated fashion using ImageJ/FIJI 1.53t. Therefore, the previously described macros^59^ were used as a blueprint and adapted.

### Perfusion of mice and sample preparation

Mice were housed on a 12 h light-dark cycle with free access to food and water at 22 ± 2 °C and a humidity of 55 ± 10%. Six male mice (C57BL/6NRj), 2–8 months of age, were anesthetized with Ketamine/Xylazine through intra-peritoneal injection. After surgical tolerance was achieved, the abdomen was opened and liver and heart uncovered. A canula was pricked into the bottom of the left heart chamber to inject PBS containing 50 mM NEM into the cardiovascular system. A drain was given by a cut above the liver. PBS was injected until the liver lost the red color and turned pale. After decapitation, liver, brain and spinal cord were removed and added to RIPA buffer (50 mM Tris/HCl pH 8.0, 150 mM NaCl, 0.1% SDS, 1% IGEPAL CA-630, 0.5% Na deoxycholate, 1 mM EDTA, freshly added protease inhibitor (*Roche*)) containing 50 mM NEM. The tissue was dounce homogenized with 1200 rpm 20 times, sonicated 10 s at 4 °C and 30% amplitude and subsequently centrifuged for 10 min at 4 °C and 4000 × *g*. The supernatant was transferred into a new tube and incubated for 1 h to let free thiols react with NEM. Afterwards, protein concentration was determined using BCA. Typically, 45 µg protein were used for SDS-PAGE for WB analysis.

### SDS-PAGE, Coomassie and Western blot

Samples were supplemented with 1× sample buffer (5×: 250 mM Tris/HCl pH 6.8, 30% glycerol, 0.1% Bromophenol blue, 10% SDS) and incubated for 5 min at 95–98 °C. We used either 5 mM β-ME for recombinant protein samples or 5 mM DTT for tissue samples or omitted it. Separation was performed with 5–12% SDS acrylamide gels using running buffer (3.029 g/L Tris, 14.4 g/L glycine, 0.1% SDS). Afterwards, gels were either stained with Coomassie (30% EtOH, 10% acetic acid, 0.25% Coomassie brilliant blue R250) or used for Western blot. Therefore, the transfer was performed semi-dry using PVDF membranes and transfer buffer (3 g/L Tris, 24.15 g/L glycine pH 8.8, 10% MeOH). Blocking was performed for 1 h at rt under shaking in blocking solution (10% BSA in TBST). Primary and secondary antibodies (diluted in 1% BSA in TBST) were applied for 1 h at rt under shaking, with a wash step of 3 × 5 min with TBST (6.5 g/L Tris, 8.75 g/L NaCl pH 7.4, 0.05% Tween20) between primary and secondary antibody. After additional washing 3 × 5 min with TBST, detection was performed using ECL and the ChemiDocTM Imaging System (*BioRad*). The following antibodies were used: anti-GephyrinE (3B11; 1:10, self-made); anti-GAPDH (1:1000, G9545, *Sigma*), anti-mouse HRP-coupled (1:10,000, AP181P, *Sigma*), anti-rabbit HRP-coupled (1:10,000, AP187P, *Sigma*).

### Electrophysiology

Miniature inhibitory postsynaptic currents (mIPSCs) were recorded using whole-cell patch clamp recordings in primary cultures of murine hippocampal neurons with various genotypes and subjected to different treatment conditions. Recordings were performed at 30 °C between days 13 and 15 in vitro (DIV). Experimental procedures essentially followed protocols previously described in Liebsch et al. (2022)^59^. Data were sampled at 20 kHz and low-pass filtered at 10 kHz with a four-pole Bessel filter. Offline, the recordings were smoothed by averaging the values in *a* ± 0.001 s interval (20 data points) around any given datapoint. The carbogenated (95% O_2_ and 5% CO_2_) extracellular saline contained (in mM): 125 NaCl, 21 NaHCO_3_, 2.5 KCl, 2 MgCl_2_, 1.2 NaH_2_PO_4_, 10 HEPES, 5 glucose, adjusted to pH 7.2 with NaOH. Glutamatergic input was blocked with 5 × 10^−5^ M DL-2-amino-5-phosphonopentanoic acid (DL-AP5; BN0086, Biotrend Chemikalien GmbH) and 10^−5^ M 6-cyano-7-nitroquinoxaline-2,3-dione (CNQX; C127, Sigma-Aldrich). Na^+^ mediated action potentials were blocked by 10^−7^ M tetrodotoxin (TTX; T-550, Alomone Labs). In the wt DAAO + D-MET group, 5 mM D- methionine (M9375, Sigma-Aldrich) was bath applied at least 1 h prior to the start of the recording. The electrode solution contained (in mM): 133 CsCl, 2 MgCl_2_, 1 CaCl_2_, 10 HEPES, 10 EGTA, adjusted with CsOH to pH 7.2. The liquid junction potential between intracellular and extracellular solution (6.3 mV) were calculated using the LJPcalc software (https://swharden.com/LJPcalc; arXiv:1403.3640v2; https://doi.org/10.48550/arXiv.1403.3640 and compensated. The holding potential was −70 mV. Because of the high intracellular chloride concentration, GABA_A_ receptor-mediated Cl^-^ currents were detected as inward currents. mIPSCs were analyzed offline from 50 s intervals taken after the recording had stabilized (∼10 min after establishing the whole-cell configuration), using the Easy Electrophysiology analysis software (v2.6.4rcz, Joseph J Ziminski, www.easyelectrophysiology.com). Events were detected by crossing a threshold that was manually set and adjusted depending on the noise level of the signal. After the events were classified as mIPSCs by template-matching (correlation cutoff: 0.80: decay search period: 50 ms; baseline search period: 10 ms), parameters including frequency, amplitude, rise and decay were determined. The recording-specific template was calculated by fitting the average of prior threshold-detected and manually reviewed events. This semi-automated detection procedure was verified by visual inspection.

### Statistics and computation

Visualization and statistical analysis were performed with GraphPad Prism6 and R vers. 4.2.1. Standard deviation, individual data points and used tests are shown in the individual graph and in the captions, respectively. Data points were identified as outliers when exceeding twofold the standard deviation. If data was normalized, *student’s* *t*-test was performed, and the significance level alpha corrected by the number of tests. Otherwise, we performed 1-way ANOVA and *Bonferroni* post-hoc tests. We analyzed: *N* = 6 individual mice for redox-dependent multimers; *N* = 3 technical replicates in the Moco activity assay; *N* = 3 technical replicates in SEC experiments; *N* = 3 technical replicates of gephyrin in ITC experiments; *N* = 3 technical replicates of gephyrin in co-sedimentation assays, *N* = 3 technical replicates of gephyrin in calpain cleavage assays; *N* = 10 cells per condition from 3 individual embryos of one neuron preparation in all experiments using hippocampal neurons. Perplexity and ChatGPT were used for language style improvement. Final Figures were created with inkscape and PowerPoint.

For Electrophysiology experiments, numerical data are presented as mean ± standard error of the mean (SEM). For pairwise comparisons of independent samples, Student’s *t*-test was applied for data with parametric distribution, while the Mann–Whitney *U* test was used for non-parametric data. Normality of data distribution was assessed using the Anderson–Darling test. For comparisons of more than two groups, Kruskal–Wallis tests were conducted followed by Dunn’s post hoc multiple comparisons tests. In boxplots, horizontal lines represent the median, + symbols denote the mean, boxes indicate the interquartile range (25th to 75th percentile), and whiskers extend to the minimum and maximum values. Exact *p*-values and sample sizes (*n*) are reported in the figures. Data analysis was performed using Spike2 and Prism 8 (GraphPad Software Inc.).

### Reporting summary

Further information on research design is available in the Nature Portfolio Reporting Summary linked to this article.

## Supplementary information

Supplementary Information

Reporting Summary

Transparent Peer Review file

## Source data

Source Data

## Data Availability

Source data are provided with this paper.

## Code availability

Code for image analysis was adapted from previous work^59^ and is available from: https://github.com/FilLieb/quantitative_synapse_analysis.
