# Supplementary material for: Redox-dependent synaptic clustering of gephyrin
Source: Nat Commun. 2026 Sep 3;17:9457. doi: 10.1038/s41467-026-77147-2 (PMC13542264; doi:10.1038/s41467-026-77147-2)
Supplement: Supplementary file 1 [file 41467_2026_77147_MOESM1_ESM.pdf]

Redox-dependent synaptic clustering of gephyrin

Maria-Theresa Gehling[1], Emanuel H. W. Bruckisch[1], Fynn R. Eggersmann[2,3], Konrad Benting[1], Lianne JHC Jacobs[1], Elmar Behrmann[1], Jan Riemer[1,3], Peter Kloppenburg[2,3], Filip Liebsch[1,5], Günter Schwarz[1,3,4,5*]

[1]Institute of Biochemistry, Department of Chemistry and Biochemistry, University of Cologne, 50674 Cologne, Germany

[2]Biocenter, Institute for Zoology, University of Cologne, Cologne 50674, Germany

[3]Cologne Excellence Cluster on Cellular Stress Responses in Aging-Associated Diseases (CECAD), University of Cologne, Cologne, Germany

[4]Center for Molecular Medicine Cologne (CMMC), University of Cologne, Cologne, Germany

[5]These authors jointly supervised this work

*Correspondence:

Guenter Schwarz, e-mail: gschwarz@uni-koeln.de, phone: +49 221 4709 6440

Filip Liebsch, e-mail: f.liebsch@uni-koeln.de, phone: +49 221 4709 6440

Supporting Figures

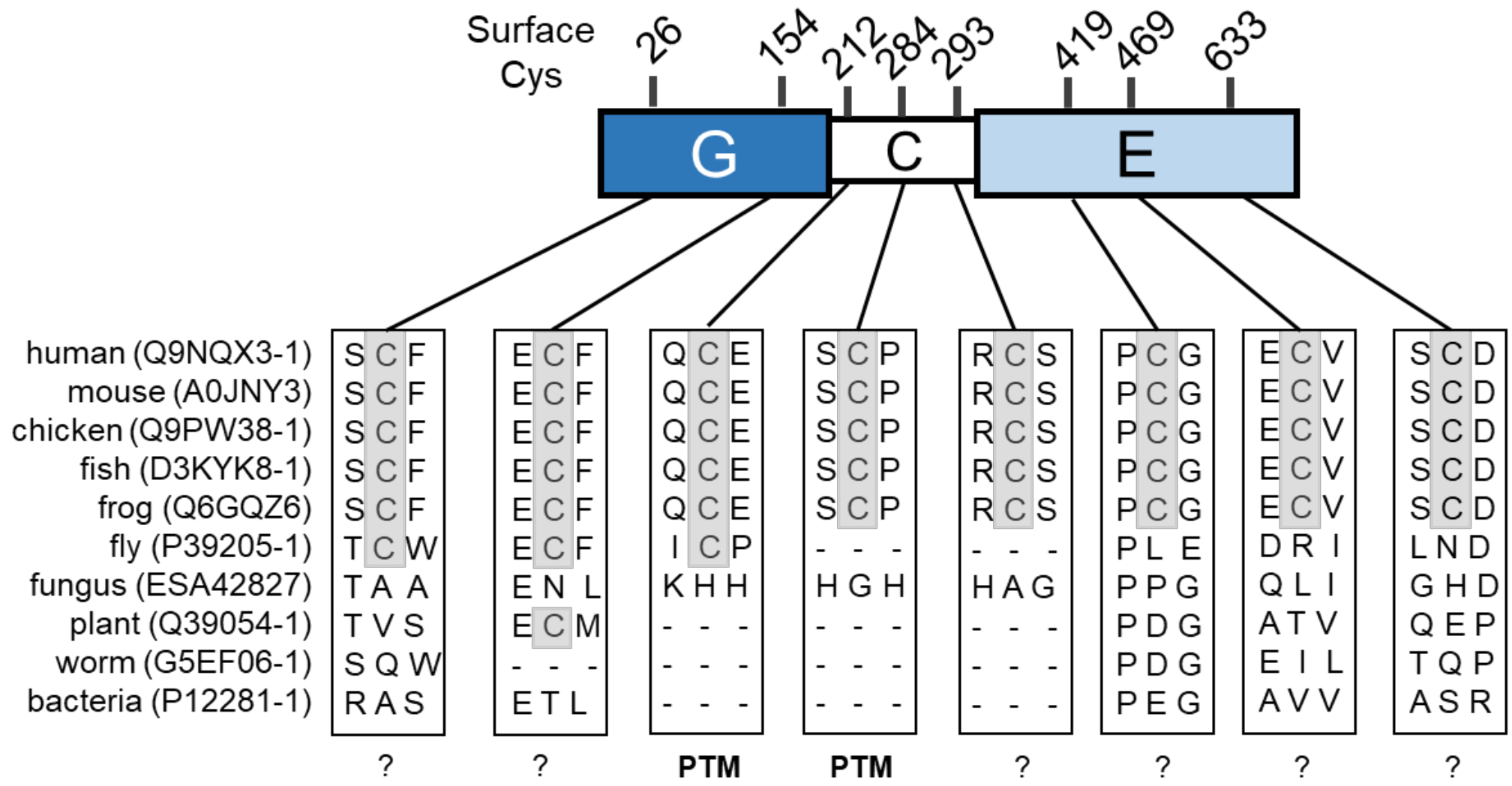

Figure S1: Alignment of gephyrin and homologues.
Alignment of protein sequence of different species. Areas around the chosen cysteines of gephyrin depicted with boxes. Cys 212 and 284 have assigned functions (targets of palmitoylation), while the function of the others is unknown (?).

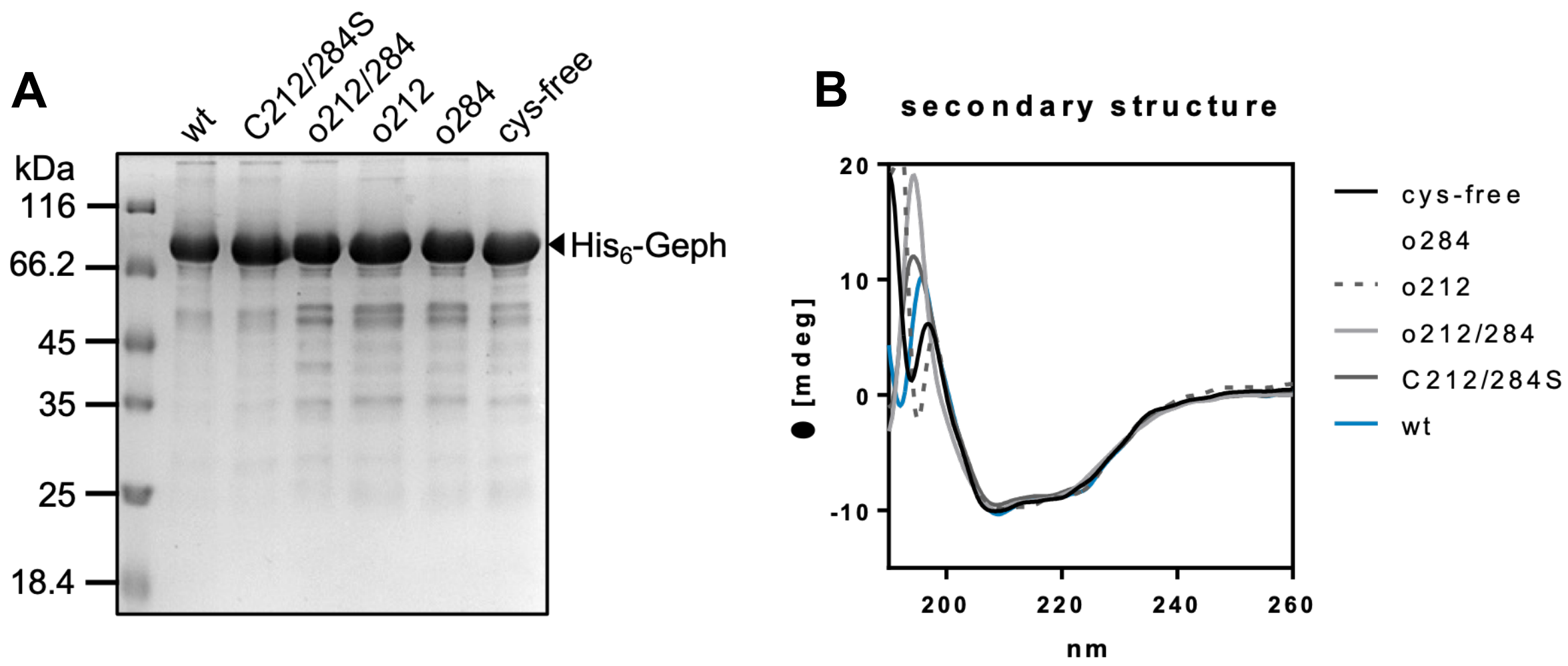

Figure S2: His$_6$-Gephyrin derived from *E. coli*.
His$_6$-Gephyrin variants recombinantly expressed and purified from *E. coli*. **(A)** Representative Coomassie gel of purified proteins, N = 3 technical replicates. **(B)** Analysis of secondary structure by CD Spec. Verical dotted line indicates data points with HT values above 800 mV (machine threshold).

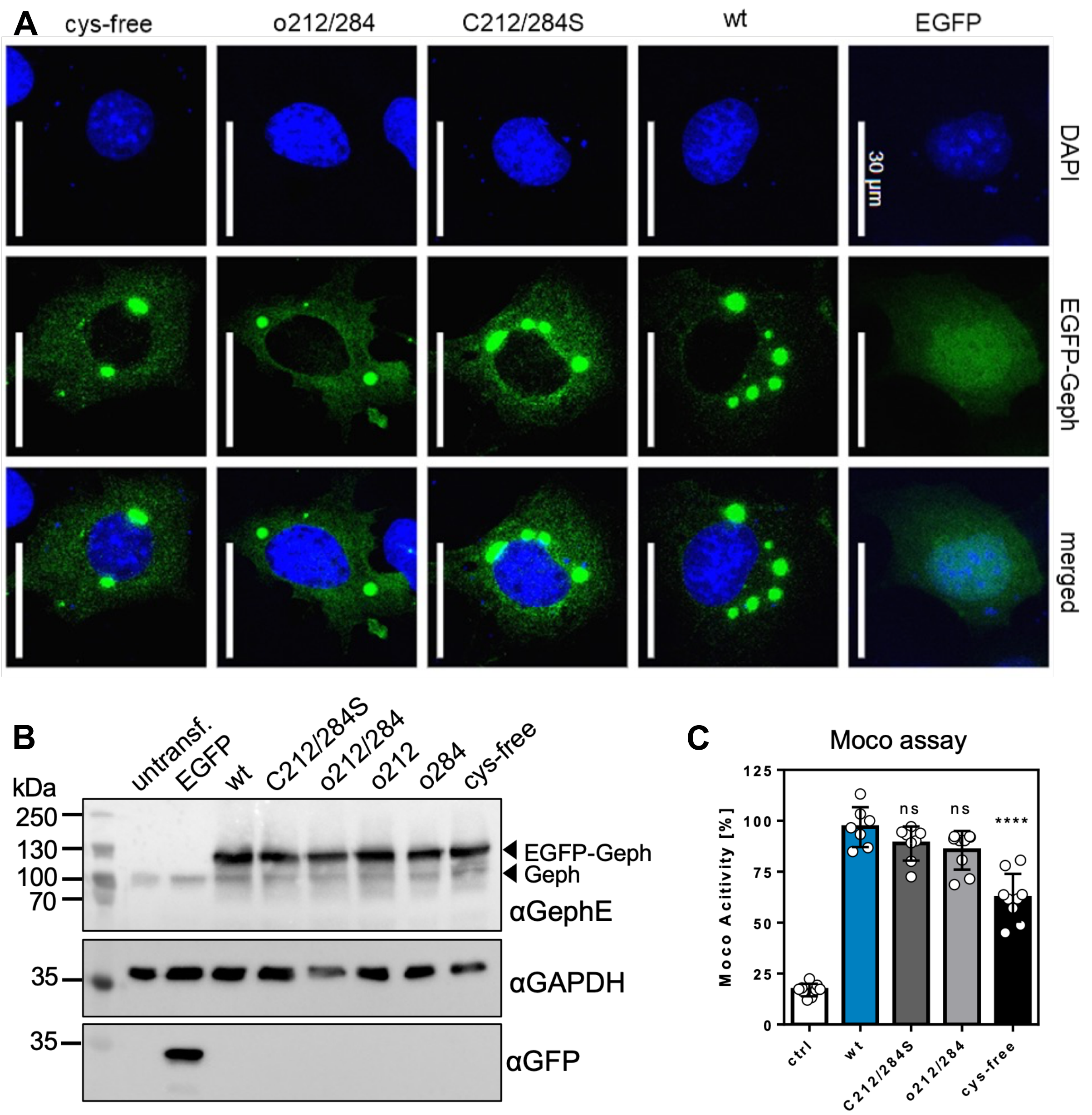

Figure S3: Expression of EGFP-Geph in COS7 and HEK293 cells.
EGFP-Gephyrin variants were recombinantly expressed in COS7 and HEK293 cells for 48 h. **(A)** Images of COS7 cells. Blue: DAPI, green: EGFP-Geph, scale bar of 30 µm. **(B)** Expression in HEK293 cells. Protein amounts analyzed by Western blot. EGFP-Geph and endogenous gephyrin marked with arrows, n = 3. **(C)** Moco assay with cysteine variants. Moco activity normalized to wt. Mean (± SD). Significance towards wt tested by two-sided t-test, alpha level corrected by three. C212/284S: p=0.3288 (ns); o212/284: p=0.1026 (ns); cys-free: p=6.1 x 10^{-5} (****).

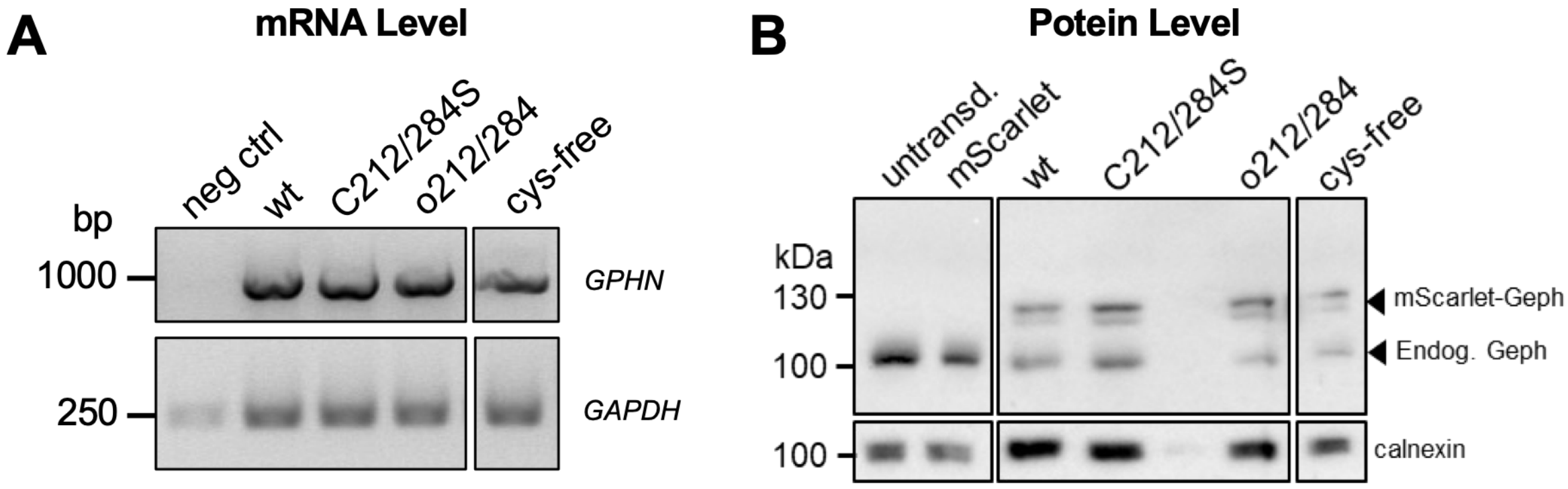

Figure S4: Expression of recombinant gephyrin in hippocampal neurons.
Expression of mScarlet-gephyrin via transduction in primary hippocampal neurons. **(A)** mRNA level determination. Isolated mRNA of mSclt-Geph was used for PCR. Primers were designed to exclusively bind to recombinant gephyrin. GAPDH used as a ctrl. **(B)** Western Blot of neuronal lysates. Endogenous and recombinant gephyrin species indicated by arrows.

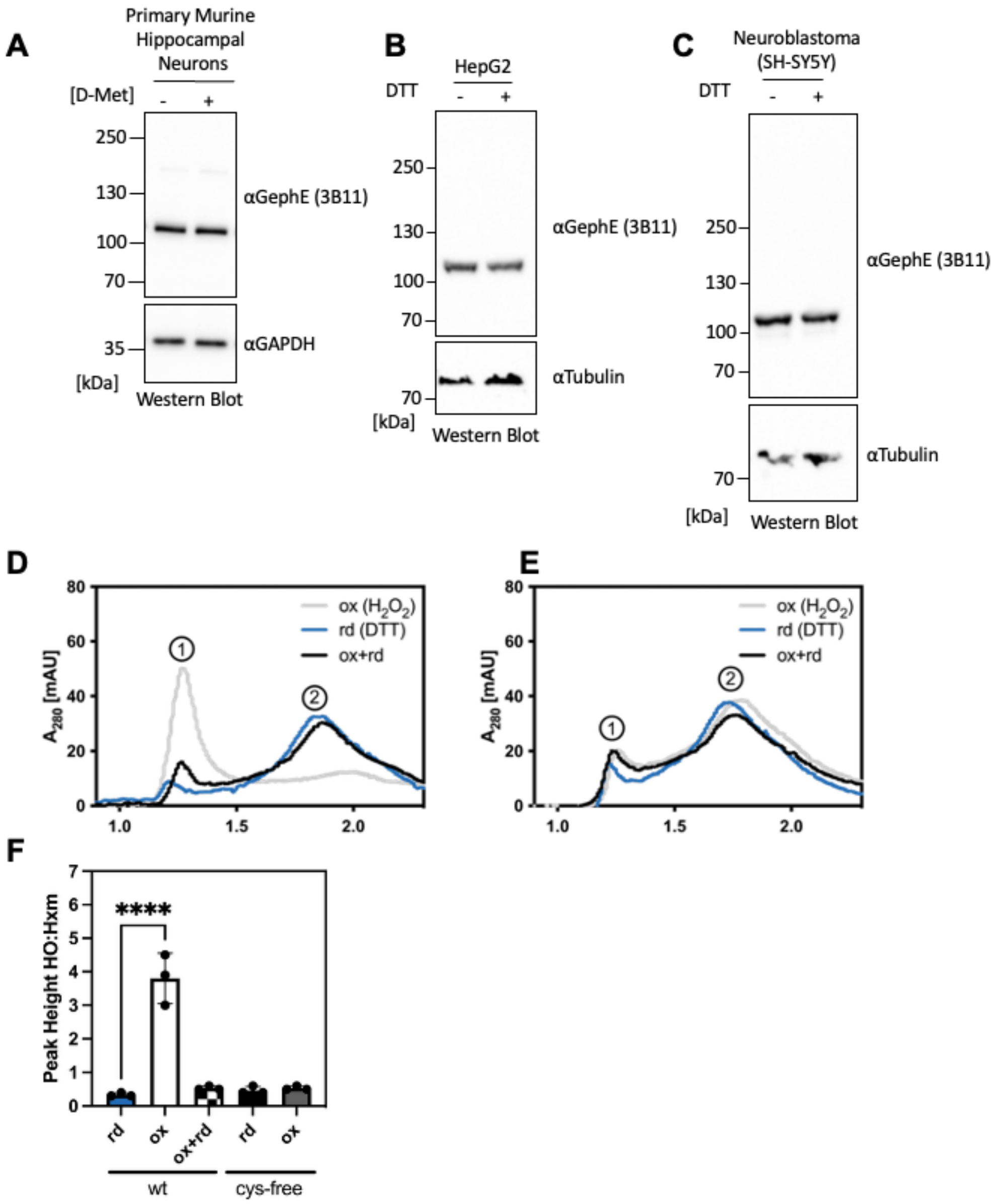

Figure S5: Western blot analysis of gephyrin in cultured cells and analysis of SEC fractions
(A–C): Western blot analysis of endogenous gephyrin detected with 3B11 antibody does not show formation of covalent higher oligomers in cultured cells. **(A)** Primary hippocampal neurons were infected with AAV2/1 DAAO and treated with 5 mM D-Met (+) or not (-) to produce intracellular H_2O_2 like in Fig. S8. GAPDH as loading control. **(B, C)** HepG2 or neuroblastoma (SH-SY5Y) cells were treated with 150 µM H_2O_2 and after lysis treated with DTT (+) or not (-). Tubulin as loading control. **(D, E)**: Size exclusion chromatography (SEC) with H_2O_2-oxidized (1 mM H_2O_2, 4°C, for 18 h) or reduced (1 mM DTT, 4°C, for 18 h) gephyrin wildtype (wt) **(D)** or cysteine-free (cys-free) variant **(E)**. Chromatograms with peaks of high-order multimers (1) or hexameric gephyrin (2). Re-reduced runs in black (1 mM H_2O_2, 4°C, for 18 h and subsequently 5 mM DTT, 22 °C, 1 h). **(F)** Quantification of the ratio of the peak heights of three different runs. Mean (± SD). Significance tested by 1way ANOVA $F(4,10)= 56.35$, $p=8.01 \times 10^{-7}$, Bonferroni post-hoc test: wt rd vs. wt ox $p=2.27 \times 10^{-6}$ (****).

A

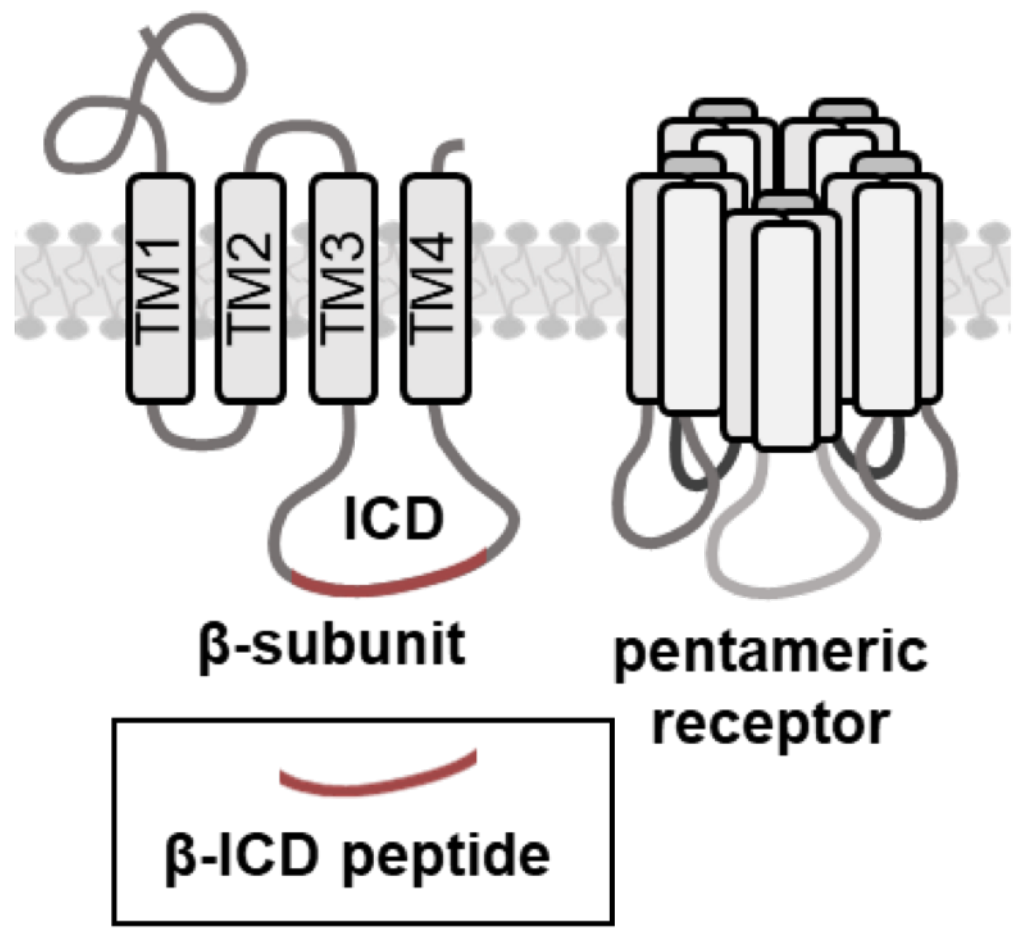

B

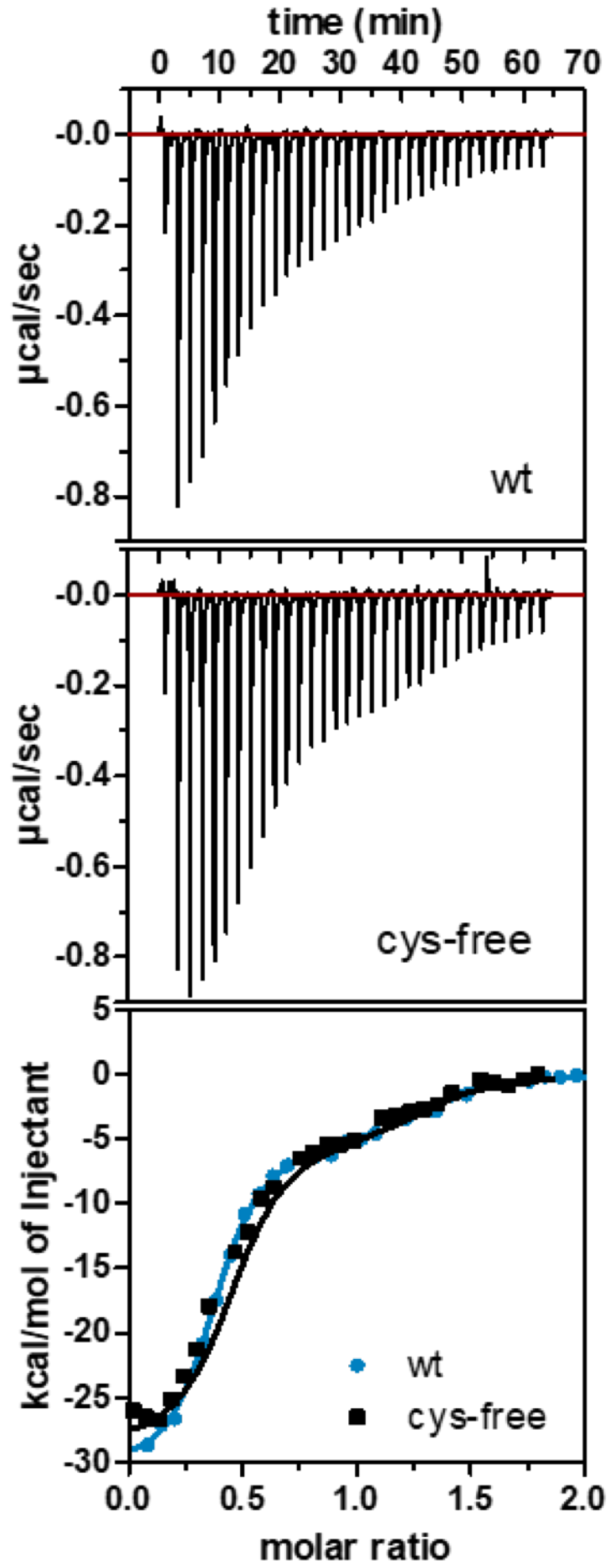

C

Variant	N^{high}	±	K_D^{high} [µM]	±	H^{high} [kcal/mol]	±	S^{high} [cal/mol/deg]
wt_1	0.302	0.006	0.0123	0.0029	-28.47	0.00	-55.60
cysfree_1	0.438	0.016	0.0763	0.0238	-31.53	0.72	-69.10
wt_2	0.360	0.005	0.0249	0.0074	-31.01	0.54	-65.20
cysfree_2	0.443	0.011	0.0391	0.0203	-29.34	0.74	-60.70
wt_3	0.349	0.006	0.0870	0.0191	-63.31	1.58	-172.00
cysfree_3	0.342	0.006	0.0735	0.0017	-65.20	1.64	-178.00
Variant	N^{low}	±	K_D^{low} [µM]	±	H^{low} [cal/mol]	±	S^{low} [cal/mol/deg]
wt_1	0.492	0.012	0.877	0.1808	-11.82	0.65	-10.40
cysfree_1	0.516	0.036	1.366	0.3919	-8.10	2.01	0.71
wt_2	0.920	0.027	0.787	0.2530	-5.40	0.43	10.50
cysfree_2	0.880	0.049	0.971	0.5316	-4.76	0.83	12.20
wt_3	0.952	0.031	1.412	0.3471	-10.93	1.13	-8.48
cysfree_3	0.964	0.030	1.259	0.3283	-10.69	-0.01	-7.49

Figure S6: Analysis of ITC Data.
(A) Cartoon depicting glycine receptor β-ICD (GlyRβICD peptide) peptide, which was used for ITC measurements. **(B, C)**: Interaction of gephyrin wt or cys-free variant towards GlyRβICD, parameters determined by mathematical fitting (two-site fit).

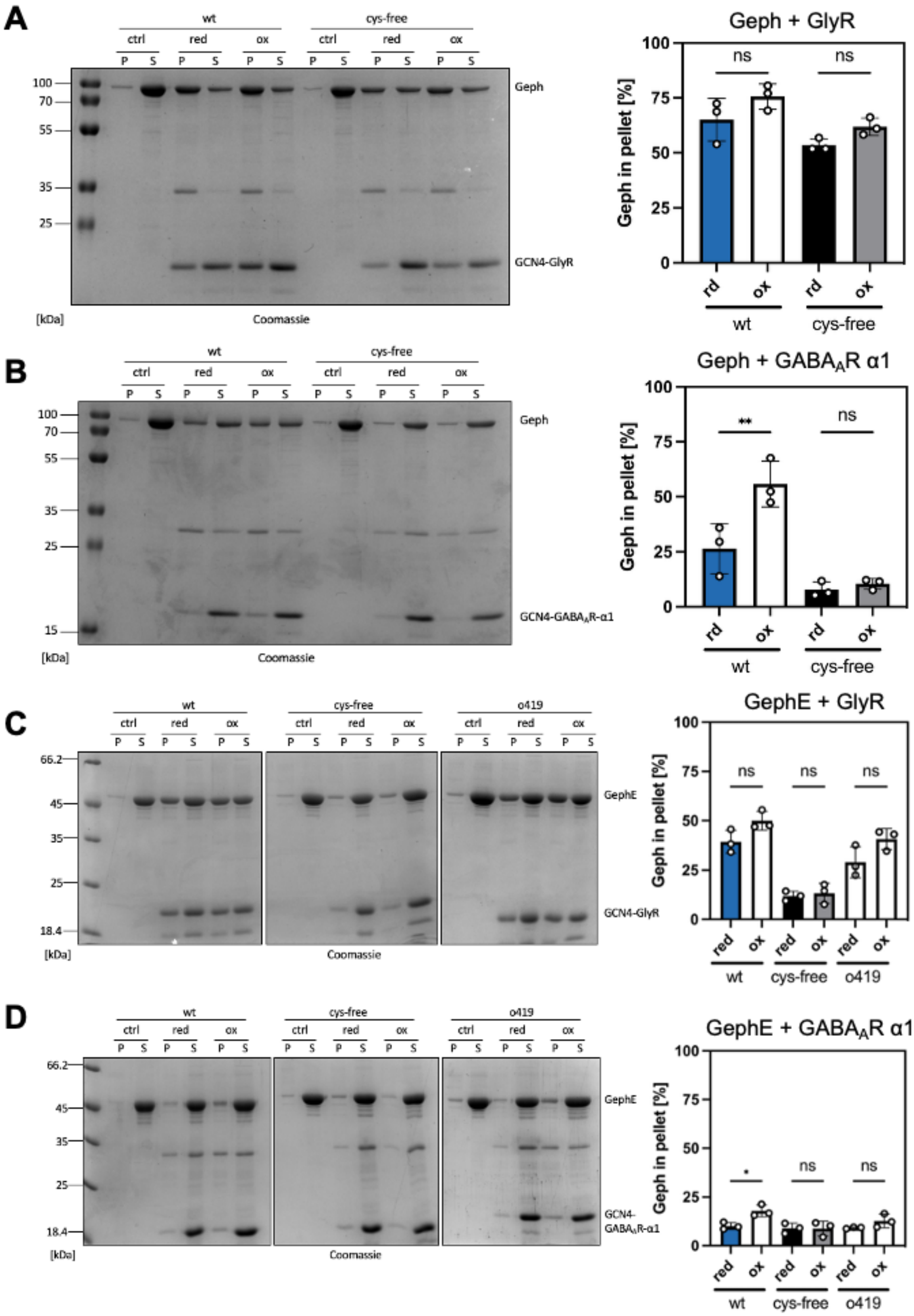

Figure S7: Liquid-liquid phase separation.

Figure S7: Liquid-liquid phase separation.

Oxidized (ox) or reduced (rd) gephyrin full length (Geph) or E-domain (GephE) wild type (wt), cysteine-free (cys-free) or only with Cys419 (o419) were used for sedimentation. n = 3 independent experiments, mean and standard deviation are displayed. **(A)** Sedimentation assay using Geph + GCN4-GlyR, **(B)** Geph + GCN4-$GABA_A$R α1, **(C)** GephE + GCN4-GlyR or **(D)** GephE + GCN4-$GABA_A$R α1. Condensed proteins are recovered from the pellet (P) and dilute proteins from the supernatant (S) fractions. As control oxidized gephyrin wt and cysteine-free alone were used. Quantification of gephyrin band intensities. Relative abundance (%) normalized to sum of protein in pellet and supernatant of the corresponding fractions. Mean (± SD).Significance tested for: **(A)** by 2way ANOVA $F(5,10)=87.63$, $p=6.31 \times 10^{-8}$. Tukey's multiple comparisons test: wt: p=0.3045>0.05 (ns); cys-free: p=0.5262>0.05 (ns); **(B)** by 2way ANOVA $F(5,10)=23.65$, $p=3.06 \times 10^{-5}$. Tukey's multiple comparisons test: wt: p=0.0053 (**); cys-free: p=0.9970>0.05 (ns); **(C)** by 2way ANOVA $F(8,16)=48.99$, $p=8.31 \times 10^{-10}$. Tukey's multiple comparisons test: wt: p=0.1085>0.05 (ns); cys-free: p=0.9999>0.05 (ns); oC419: p=0.0624>0.05 (ns); **(D)** by 2way ANOVA $F(8,16)=6.121$, $p=0.0011$. Tukey's multiple comparisons test: wt: p=0.0376 (*); cys-free: p=0.9999>0.05 (ns); oC419: p=0.7267>0.05 (ns)

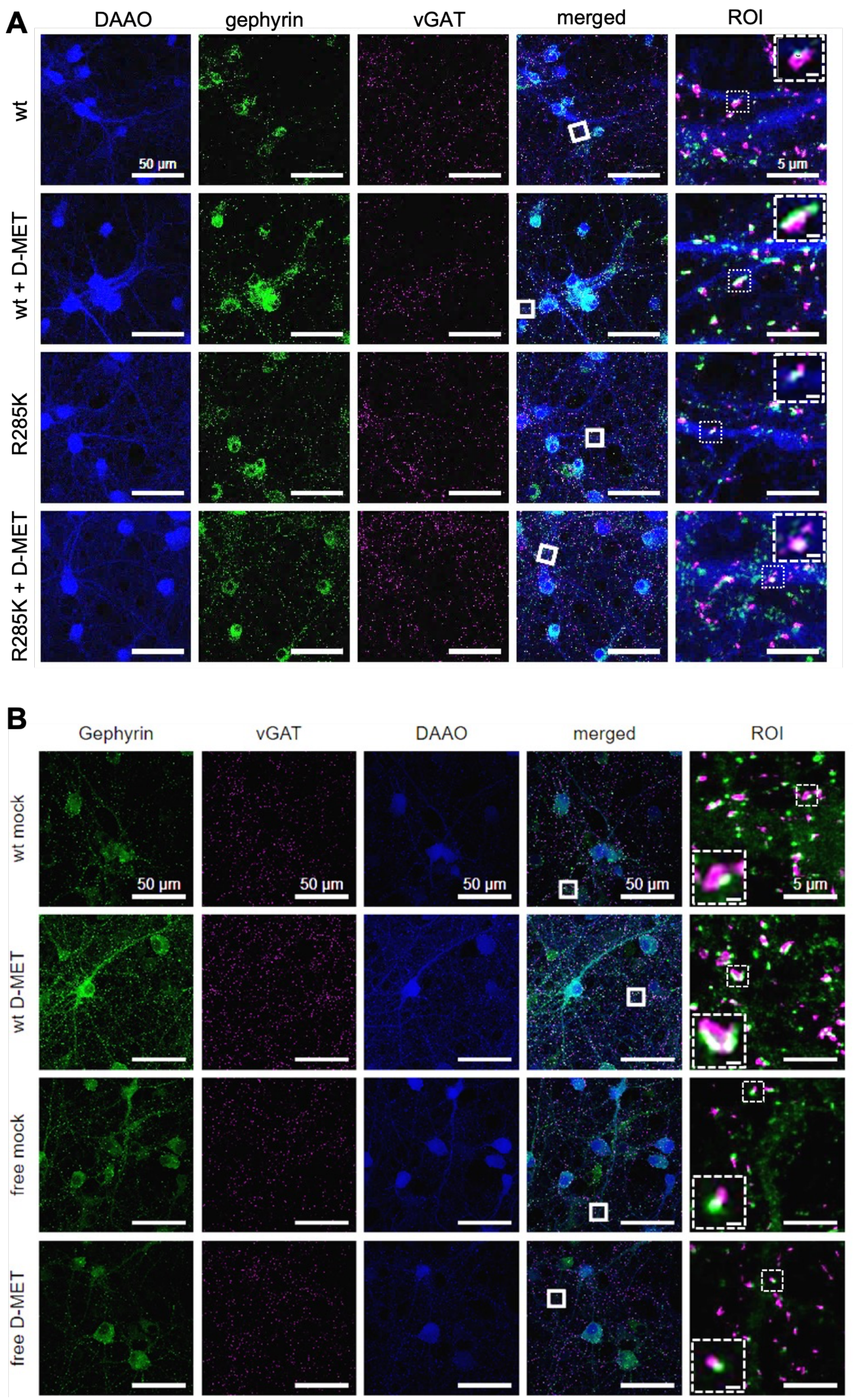

91 **Figure S8: H_2O_2 drives gephyrin oligomerization at synapses.**
92 Expression of cytosolic mSclt-DAAO and mEGFP-Geph in primary, hippocampal neurons. Treatment at
93 DIV14 with 5 mM D-MET. Analysis of cluster sizes [µm^2], intensity [au] and co-localization with vGAT
94 (=synaptic clusters) of gephyrin. **(A)** mSclt-DAAO wt and R285K. Analysis of endogenous gephyrin
95 clusters. Gephyrin: green, vGAT: magenta, DAAO: blue; scale bar 30 and 0.5 µm. **(B)** mSclt-DAAO wt
96 and mEGFP-Geph wt and cys-free variants. Analysis of recombinant mEGFP-gephyrin
97 clusters. Gephyrin: green, vGAT: magenta; scale bar 30 and 0.5 µm.

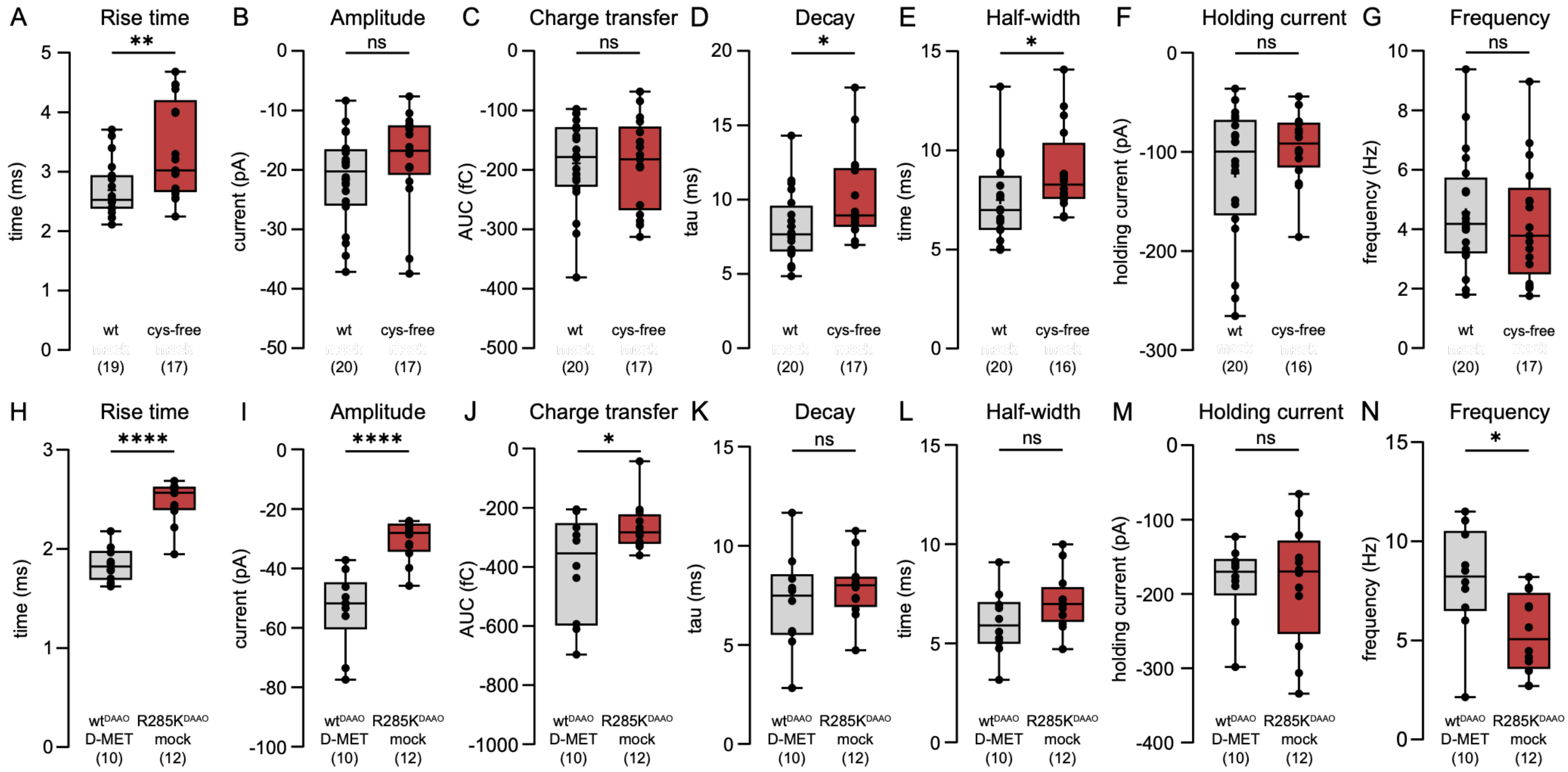

Figure S9: Supplementary data for Figure 6. Box plots showing basic parameters of miniature inhibitory postsynaptic currents (mIPSCs) recorded in primary cultures of murine hippocampal neurons. Each data point represents the mean of the mIPSCs recorded during a 50 s interval from a single neuron (260 ± 17 individual mIPSCs per neuron). All recordings were performed at 13–15 DIV. Glutamatergic input was blocked by CNQX and DL-AP5. Na^+-mediated action potentials were blocked by TTX. Holding potential was −70 mV. In box plots, horizontal lines represent the median, “+” symbols denote the mean, boxes indicate the interquartile range (25th–75th percentile), and whiskers extend to the minimum and maximum values. Exact p-values are reported. Student's *t*-test is indicated as *t*-test, Welch-corrected *t*-test as Welch *t*-test, and the Mann-Whitney *U* test as *U* test. Statistical significance is indicated as follows: * $p < 0.05$, ** $p < 0.01$, *** $p < 0.001$, and **** $p < 0.0001$. (**A**–**G**) wt and cys-free. (**H**–**N**) wt DAAO + D-MET treatment (wt^{DAAO} D-MET) and R285K DAAO + mock treatment ($R285K^{DAAO}$ mock). (**A**, **H**) Rise time. (**B**, **I**) Amplitude. (**C**, **J**) Charge transfer. (**D**, **K**) Decay time constant. (**E**, **L**) Half-width. (**F**, **M**) Holding current. (**G**, **N**) Frequency. The number of recorded neurons for each panel is given in parentheses. Sample sizes: (**A**–**G**) wt, 4 mice; cys-free, 5 mice; (**H**–**N**) wt^{DAAO} D-MET, 2 mice; $R285K^{DAAO}$ mock, 3 mice. Data are presented as mean ± SEM. Charge transfer is reported as the signed integral of the current trace.

wt vs. cys-free: (**A**) Rise time: wt (n = 19), 2.7 ± 0.1 ms; cys-free (n = 17), 3.3 ± 0.2 ms. *U* test, $U = 71$, $p = 0.0035$. (**B**) Amplitude: wt (n = 20), −22 ± 2 pA; cys-free (n = 17), −18 ± 2 pA. *U* test, $U = 120$, $p = 0.1326$. (**C**) Charge transfer: wt (n = 20), −189 ± 17 fC; cys-free (n = 17), −188 ± 18 fC. *t*-test, $t(35) = 0.030$, $p = 0.9764$. (**D**) Decay time constant: wt (n = 20), 8.0 ± 1.0 ms; cys-free (n = 17), 10 ± 1 ms. *U* test, $U = 93$, $p = 0.0184$. (**E**) Half-width: wt (n = 20), 7.5 ± 0.5 ms; cys-free (n = 16), 9.0 ± 0.5 ms. *U* test, $U = 90$, $p = 0.0256$. (**F**) Holding current: wt (n = 20), −122 ± 15 pA; cys-free (n = 16), −95 ± 9 pA. *U* test, $U = 133$, $p = 0.4036$. (**G**) Frequency: wt (n = 20), 4.5 ± 0.4 Hz; cys-free (n = 17), 4.2 ± 0.5 Hz. *t*-test, $t(35) = 0.552$, $p = 0.5847$.

wt^{DAAO} D-MET vs. $R285K^{DAAO}$ mock: (**H**) Rise time: wt^{DAAO} D-MET (n = 10), 1.8 ± 0.1 ms; $R285K^{DAAO}$ mock (n = 12), 2.5 ± 0.1 ms. *U* test, $U = 3$, $p = 2.17 \times 10^{-5}$. (**I**) Amplitude: wt^{DAAO} D-MET (n = 10), −54 ± 4 pA; $R285K^{DAAO}$ mock (n = 12), −30 ± 2 pA. *t*-test, $t(20) = 5.417$, $p = 2.66 \times 10^{-5}$. (**J**) Charge transfer: wt^{DAAO} D-MET (n = 10), −402 ± 56 fC; $R285K^{DAAO}$ mock (n = 12), −264 ± 24 fC. Welch *t*-test, $t(12.31) = 2.271$, $p = 0.0419$. (**K**) Decay time constant: wt^{DAAO} D-MET (n = 10), 7.0 ± 1.0 ms; $R285K^{DAAO}$ mock (n = 12), 8.0 ± 0.5 ms. *t*-test, $t(20) = 0.888$, $p = 0.3853$. (**L**) Half-width: wt^{DAAO} D-MET (n = 10), 6.0 ± 0.5 ms; $R285K^{DAAO}$ mock (n = 12), 7.0 ± 0.5 ms. *t*-test, $t(20) = 1.649$, $p = 0.1148$. (**M**) Holding current: wt^{DAAO} D-MET (n = 10), −183 ± 16 pA; $R285K^{DAAO}$ mock (n = 12), −186 ± 24 pA. *t*-test, $t(20) = 0.090$, $p = 0.9293$. (**N**) Frequency:

wt^{DAAO} D-MET (n = 10), 8.0 ± 1.0 Hz; R285K^{DAAO} mock (n = 12), 5.0 ± 0.6 Hz. t-test, $t(20)$ = 2.689, p = 0.0141.
