# Supplementary material for: Redox-dependent synaptic clustering of gephyrin
Source: Nat Commun. 2026 Sep 3;17:9457. doi: 10.1038/s41467-026-77147-2 (PMC13542264; doi:10.1038/s41467-026-77147-2)
Supplement: Supplementary file 2 [file 41467_2026_77147_MOESM2_ESM.pdf]

nature portfolio

Corresponding author(s): Günter Schwarz, Filip Liebsch

Last updated by author(s): Aug 11, 2026

Reporting Summary

Nature Portfolio wishes to improve the reproducibility of the work that we publish. This form provides structure for consistency and transparency in reporting. For further information on Nature Portfolio policies, see our Editorial Policies and the Editorial Policy Checklist.

Statistics

For all statistical analyses, confirm that the following items are present in the figure legend, table legend, main text, or Methods section.

n/a	Confirmed	
☐	☒	The exact sample size (*n*) for each experimental group/condition, given as a discrete number and unit of measurement
☐	☒	A statement on whether measurements were taken from distinct samples or whether the same sample was measured repeatedly
☐	☒	The statistical test(s) used AND whether they are one- or two-sided<br>*Only common tests should be described solely by name; describe more complex techniques in the Methods section.*
☒	☐	A description of all covariates tested
☐	☒	A description of any assumptions or corrections, such as tests of normality and adjustment for multiple comparisons
☐	☒	A full description of the statistical parameters including central tendency (e.g. means) or other basic estimates (e.g. regression coefficient) AND variation (e.g. standard deviation) or associated estimates of uncertainty (e.g. confidence intervals)
☐	☒	For null hypothesis testing, the test statistic (e.g. *F*, *t*, *r*) with confidence intervals, effect sizes, degrees of freedom and *P* value noted<br>*Give P values as exact values whenever suitable.*
☒	☐	For Bayesian analysis, information on the choice of priors and Markov chain Monte Carlo settings
☒	☐	For hierarchical and complex designs, identification of the appropriate level for tests and full reporting of outcomes
☒	☐	Estimates of effect sizes (e.g. Cohen's *d*, Pearson's *r*), indicating how they were calculated

Our web collection on statistics for biologists contains articles on many of the points above.

Software and code

Policy information about availability of computer code

Data collection	LAS X (Leica), Spectra Analysis version 1.53.07 of Spectra Manage version 1.54.0. (CD-Spectroscopy), Spark Control V2.3, ChemiDocTM Imaging System (BioRad)
Data analysis	MicroCal Analysis, Origin7, ImageJ/FIJI (version 1.53c), Image Lab, LJPcalc software (https://swharden.com/LJPcalc; arXiv:1403.3640v2; https://doi.org/10.48550/arXiv.1403.3640, Easy Electrophysiology analysis software (v2.6.4rcz, Joseph J Ziminski, www.easyelectrophysiology.com), GraphPad Prism6 and R vers. 4.2.1, Spike2 and Prism 8 (GraphPad Software Inc.)

For manuscripts utilizing custom algorithms or software that are central to the research but not yet described in published literature, software must be made available to editors and reviewers. We strongly encourage code deposition in a community repository (e.g. GitHub). See the Nature Portfolio guidelines for submitting code & software for further information.

Data

Policy information about availability of data

All manuscripts must include a data availability statement. This statement should provide the following information, where applicable:

- Accession codes, unique identifiers, or web links for publicly available datasets
- A description of any restrictions on data availability
- For clinical datasets or third party data, please ensure that the statement adheres to our policy

Source data are provided with this paper.

Research involving human participants, their data, or biological material

Policy information about studies with human participants or human data. See also policy information about sex, gender (identity/presentation), and sexual orientation and race, ethnicity and racism.

Reporting on sex and gender	Our research did not involve human participants, their data or biological material. Sex and gender were not considered in the study design
Reporting on race, ethnicity, or other socially relevant groupings	Our research did not involve human participants, their data or biological material. Race, ethnicity or other social groupings were not considered in the study design
Population characteristics	see above
Recruitment	Our research did not involve human participants, their data or biological material
Ethics oversight	see above

Note that full information on the approval of the study protocol must also be provided in the manuscript.

Field-specific reporting

Please select the one below that is the best fit for your research. If you are not sure, read the appropriate sections before making your selection.

☒ Life sciences ☐ Behavioural & social sciences ☐ Ecological, evolutionary & environmental sciences

For a reference copy of the document with all sections, see nature.com/documents/nr-reporting-summary-flat.pdf

Life sciences study design

All studies must disclose on these points even when the disclosure is negative.

Sample size	No sample size calculation was performed, sample sizes are consistent with standard practices in the field for these types of assays:<br><br>For redox-dependent multimers: 6 mice were analyzed.<br>For biochemistry studies: 3 technical replicates from one purification.<br>For hippocampal neuron studies: exact numbers are indicated in the figure legends.
Data exclusions	In general, no data was excluded from analysis.
Replication	For sedimentation/ITC/Moco/Calpain/LLPS experiments three technical replicas were considered. All attempts at replication were successful.
Randomization	Randomization is not relevant for experiments shown in this study as they are biological studies with well-defined parameters and statistics and their results show low variations that are numerically defined.
Blinding	In general, validation of all experiments presented in this study relied completely on statistics and well defined parameters thus blinded evaluation of data was not necessary. A bias due to omission of blinding is not expected to affect the experiments performed in this study.

Reporting for specific materials, systems and methods

We require information from authors about some types of materials, experimental systems and methods used in many studies. Here, indicate whether each material, system or method listed is relevant to your study. If you are not sure if a list item applies to your research, read the appropriate section before selecting a response.

Materials & experimental systems

n/a	Involved in the study	
☐	☒	Antibodies
☐	☒	Eukaryotic cell lines
☒	☐	Palaeontology and archaeology
☐	☒	Animals and other organisms
☒	☐	Clinical data
☒	☐	Dual use research of concern
☒	☐	Plants

Methods

n/a	Involved in the study	
☒	☐	ChIP-seq
☒	☐	Flow cytometry
☒	☐	MRI-based neuroimaging

April 2023

Antibodies

Antibodies used	anti-vesicular GABA transporter (vGAT) (1:1000, #131003) for inhibitory presynaptic terminals<br>anti-GABAAR γ2 (1:500, #224004) for postsynaptic GABAARs<br>anti-GephE (3B11) (1:20, Self-made, RRID: AB_887719), for WB<br>anti-GAPDH (1:1000, Sigma G9545), for WB<br>anti-Tubulin (1:1000, Cell signaling #2128), for WB<br>anti-mouse HRP-coupled (1:10000, Sigma AP181P), for WB<br>anti-rabbit HRP-coupled (1:10000, Sigma AP187P), for WB
Validation	anti-vesicular GABA transporter (vGAT) (#131003): KO validation, see company website https://sysy.com/product/131003<br>anti-GABAAR γ2 (1:500, #224004): validation for various applications, see company website https://sysy.com/product/224004<br>anti-GephE (3B11) (1:20, Self-made, RRID: AB_887719) validation: https://scicrunch.org/resolver/RRID:AB_887719<br>all other antibodies are widely used housekeeping protein antibodies

Eukaryotic cell lines

Policy information about cell lines and Sex and Gender in Research

Cell line source(s)	HEK293T cells (DSMZ no. ACC 635)<br>COS7 (DSMZ no. ACC 60)<br>SH-SY5Y (DSMZ no. ACC 209)<br>HepG2 (DSMZ no. ACC 180)
Authentication	cell line was not authenticated
Mycoplasma contamination	cell line tested negative for mycoplasma contamination
Commonly misidentified lines<br>(See ICLAC register)	n/a

Animals and other research organisms

Policy information about studies involving animals; ARRIVE guidelines recommended for reporting animal research, and Sex and Gender in Research

Laboratory animals	C57BL/6NRj
Wild animals	n/a
Reporting on sex	Dissociated primary hippocampal cultures were prepared from embryos of either sex.
Field-collected samples	n/a
Ethics oversight	We complied with all relevant ethical regulations for animal testing and research. Experiments were approved by the local research ethics committees (Germany, Landesamt für Natur, Umwelt und Verbraucherschutz Nordrhein-Westfalen, reference 2019.A077, reference 2021.A450, reference 2024-561).

Note that full information on the approval of the study protocol must also be provided in the manuscript.

Plants

Seed stocks	n/a
Novel plant genotypes	n/a
Authentication	n/a
