# Supplementary material for: Redox-dependent synaptic clustering of gephyrin
Source: Nat Commun. 2026 Sep 3;17:9457. doi: 10.1038/s41467-026-77147-2 (PMC13542264; doi:10.1038/s41467-026-77147-2)
Supplement: Supplementary file 3 [file 41467_2026_77147_MOESM3_ESM.pdf]

nature portfolio

Peer Review File

Redox-dependent synaptic clustering of gephyrin

Corresponding Author: Professor Guenter Schwarz

This file contains all reviewer reports in order by version, followed by all author rebuttals in order by version.

Version 0:

Reviewer comments:

Reviewer #1

(Remarks to the Author)
In this manuscript, Gehling et al., build on the earlier work from the same laboratory and determine the role of mitochondrial ROS on gephyrin clustering. Using a range of in vitro biochemical assays they describe the contribution of several surface-exposed cysteine residues for ROS-induced phase separation, increased receptor binding, and reduced proteolytic cleavage by calpain1. Using primary neurons treated with antimycin the authors link mROS to gephyrin clustering at GABAergic synaptic sites and using DTT they show the reversible nature of gephyrin clustering changes in response to ROS. Interestingly, the authors demonstrate that MoCo function of gephyrin is independent of oxidative status.

The experiments outlined are well performed with relevant statistical tests. The results described are original and relevant to the field as ROS-induced neuronal cell death is a leading hypothesis for healthy aging and neurodegeneration. A major caveat of this study is the in vitro nature of all the data presented in vitro, the timeline of responses in their study and in vivo remains unclear.

Major clarifications are required to be consistent with published literature on this topic.

1. Accardi et al., which has been cited by the authors reported mROS effects within 5 min of mROS induction in acute slices. Importantly, the abundant a1 containing GABAARs did not respond to mROS, while the less abundant a3 containing GABAARs were recruited within the 5-minute time frame to increase GABAergic amplitude and frequency after ROS. The mechanistic basis for this GABAAR subunit selectivity is not clear from the current data.

2. Phase separation drives the compartmentalization of intracellular contents into various biomolecular condensates. Authors cite work on gephyrin phase separation from Bai et al, 2021, however, in this study Bai et al., reported that phase separation of GlyR and gephyrin is sensitive to molar ratios and salt concentration. Also, gephyrin/a3 GABAAR subunit LD complex phase separation occurred at 5microM conc and was concentration dependent. Importantly, a1LD/ gephyrin phase separation did not follow this rule.
This aspect of the subunit differences in mROS-mediated gephyrin phase separation would have clarified why less abundant a3 GABAARs are recruited to inhibitory synaptic sites after mROS in neurons.

Proteins diffuse in the plane of the membrane before their accumulation at synapses, it was reported by Lee et al., 2024 that gephyrin condensates with distinct protein composition coexist with multicomponent systems. The authors reported that C domain substitutions on gephyrin from DDX4 or G3BP1 can still induce gephyrin clustering and phase separation. IQSEC3 and NLGN2 can induce gephyrin condensation. These aspects of the results are relevant to this manuscript and should be discussed sufficiently.

3. Timeline of hydrogen peroxide, antimycin, D-MET, DTT treatments is not clear. Also, it is not clear at what time point after the treatment the primary neurons were fixed and analyzed.

4. Acute hippocampal slices expressing gephyrin cysteine mutants with intracellular antimycin A application for mROS effects would be very convincing in vivo results to support all the in vitro data.

Reviewer #2

(Remarks to the Author)

Gehling and colleagues perform a comprehensive biochemical investigation of the role of reactive oxygen species (ROS) in triggering the multimerization of the scaffolding protein, gephyrin, and its role in clustering at inhibitory synapse. The authors' work builds upon previous publications in the hippocampus (Penna et al, 2014) and cerebellum (Accardi et al, 2014 and 2015; Larsen et al 2020) that have shown that ROS derived from the mitochondria and/or neuronal nitric oxide synthase strengthen inhibitory synapses. Although the clustering of gephyrin has been previously linked to the strengthening of inhibitory synapses, the possibility that ROS may trigger the clustering of gephyrin has not been tested.

Critique:
The authors present a comprehensive biochemical study of the events that lead to the multimerization and eventual clustering of gephyrin. In doing so, they pinpoint mitochondrial ROS as the key trigger in these events which react with exposed cysteine residues on gephyrin to promote the formation of disulfide bounds. The study adds to previous studies identifying palmitoylation (2014 Dejanovic et al), nitrosylation (2014 Dejanovic et al) and phosphorylation (2011 Tygarajan et al) as mechanisms of posttranslational modification that control the multimerization of gephyrin. The authors' findings are novel in not only identifying a new mechanism of gephyrin clustering but also because they fill an important gap from previous studies showing that ROS strengthens inhibitory synapses.

Despite my enthusiasm for their work, the authors, nevertheless, have several major issues to resolve to make this study appeal to the broad readership of the Journal.

First, the authors need to better articulate in the Abstract and Introduction how most researchers view the mechanisms that lead to inhibitory synapse strengthening. Most researchers currently conclude that phosphorylation of gephyrin (not disulphide bond formation) is the key step in promoting clustering and synapse strengthening. Prior work from the authors' lab has also shown a role for palmitoylation and nitrosylation too so these mechanism need to be mentioned. The manuscript text should reflect these different mechanisms and explain why prior work prompted a new study on ROS and the crosslinking of cysteine residues on gephyrin. There are also studies showing that not all GABAR inhibitory synapses contain gephyrin so there is certainly a case to be made to why there are deficits in the gephyrin phosphorylation hypothesis. As it currently stands, the Abstract and Introduction focus only on redox-dependent regulation but would be better if a more nuanced perspective is offered.

Second, most of the biochemical and imaging data in the manuscripts seems to be showing that not all gephyrin inhibitory synapses are affected by the redox state of gephyrin. For example, the clustering WT data shows population of synapses which have similar puncta size as the cys-free dataset as well as punctae which are clearly much larger. How do the authors interpret this point? Is it consistent with Accardi et al (2014) that have argued that strengthening occurs only at alpha3 and NOT alpha1 synapses? Or is it because another mechanism (e.g. palmitoylation/nitrosylation/phosphorylation) is also at play which targets a different population of gephyrin/GABAR complexes? The study would be much stronger if the authors acknowledged this variability in their dataset and offered some explanations to why it may occur. They may even consider arguing for two populations in their WT dataset.

Third, the authors' biochemical and imaging work would be greatly complemented with a patch clamp electrophysiological recordings from their hippocampal neurons to show that antimycin-A (in figure 6) triggers an increase in synapse strengthening. The authors could record mini-events and plot the effect of antimycin-A over time (mIPSC amplitude and frequency) . The absence of functional data in the study greatly diminishes the potential impact and appeal of the authors' work to the broader neuroscience community.

Fourth and finally, there are numerous formatting errors in the manuscript that include the mislabelling of figure axis to the duplication of citations in the reference list. These oversights need to be fully corrected.

Summary of the findings:
In figure 1, the authors examine whether the redox state of cysteines are important for the oligomerization of gephyrin. Their data establishes that in tissue taken from brain, liver and spinal cord, gephyrin exists in both monomeric and multimeric gephyrin forms. In the brain, about 20% of gephyrin is multimeric in basal conditions leaving a surplus of 80% monomeric forms.

In figure 2, the authors consider the possibility that 8 exposed cysteines are the target of ROS. They observe that single point mutations in each Cys of gephyrin with a Ser residue has no effect on clustering in cultured hippocampal neurons. However, compared to the WT phenotype, mutation of all variants, that is C212/284S (palmitoylation dependent mutants), o2212/284 (two cysteines remaining) and cys-free gephyrin eliminated the ability of gephyrin to cluster.

In figure 3, the authors test the possibility that exposed cysteines form disulphide bridges that mediate synaptic clustering. Using size-exclusion chromatography, they observe high order multimers that are not observed in cys-free gephyrin. The formation of disulphide bond was confirmed by using DTT (Fig. 3a,b). The authors conclude that the mechanism of redox-dependent multimerization is due to the formation of disulphide bonds. Authors then examine the functionality of gephyrin multimers by looking at their secondary structure (Fig. 3c) and ability to bind to the glycine receptor beta subunits peptides (3d-f). The authors observed no difference in the secondary structure or binding ability of gephyrin wildtype or cys-free variants. The authors conclude that the presence or absence of disulphide bonds do not alter the secondary structure or

binding ability (at least for the Glycine receptor) when existing in a monomeric or multimeric form.

In figure 4, the authors examined the potential role of the redox state of whether gephyrin in forming liquid-liquid phase separation. Using the glycine beta subunit pentamers, the authors find that oxidised WT gephyrin has a strong sedimentation properties that are absent in cys-free gephyrin (Fig. 4a-b). The authors conclude that this finding favours again that oxidised gephyrin forms high-order oligomeric clusters. The authors then look at whether the redox state is affected by calpain cleavage of gephyrin, which may be important in regulating synaptic clustering. The authors note that the ability of calpain to cleave gephyrin was reduced about two-fold for the oxidised form compared to the reduced form (Fig 4d, left). This distinction was not observed for cys-free gephyrin (Fig 4d, right). The authors also show that the remaining undigested gephyrin shows resistance to calpain digestion that was not observed in the cys-free gephyrin (Fig. 4e). Together, the authors conclude that the structural properties of gephyrin are affected by its redox state (i.e. phase separation and proteolytic sensitivity).

In figure 5, the authors examined whether the release of cytosolic hydrogen peroxide affects the clustering of endogenous (Fig. 5a) and recombinant gephyrin (wt and cys-free, Fig. 5b). To do this, the authors expressed D-amino oxidase in hippocampal neurons, which generates hydrogen peroxide when exposed to D-Met. Synaptic clustering of gephyrin was assessed by co-localization with the presynaptic marker, V-GAT. The authors report that D-amino acid oxidase and gephyrin clustering is increased by about 20 and 14 % respectively (Fig 5g-h). I believe that the y-axis labelling of Fig 5 d and e is incorrect so there was some confusion between the information in the text and figures. It was also unclear why the intensity of mEGFP-tagged gephyrin decreased following D-Met treatment. Some explanation is required. The authors conclude that oxidation of gephyrin with cytosolic hydrogen peroxide promotes clustering of gephyrin.

Finally, in figure 6, the authors repeat the experiments of figure 5 but elevate reactive oxygen species (ROS) using antimycin A which uncouples the ability of mitochondria to generate ATP through proton transport allowing molecular oxygen to react with protons to form ROS. Antimycin treatment caused a 13% increase in cluster size and 25% increase in intensity of gephyrin staining (Fig. 6d and e) that was reversed by treatment with DTT.

Specific Points:

Abstract. The narrative of the abstract needs to be toned down or at least more carefully articulated. The predominant view is that GABAR inhibitory strengthening is mediated by the actions of kinases that are activated by elevations in cytosolic Ca2+ (e.g. 2020, Barberis). The observations establishing a link between ROS and inhibitory strengthening is distinct in this regard having been shown in molecular layer interneurons (Accardi et al, Nat Comm; Larsen et al JNS), granule cells (Accardi et al, JNS) and hippocampal neurons (Penna et al). I appreciate that the Schwarz lab has added to this perspective significantly through biochemical studies but the rest of the field is still focused on the phosphorylation of gephyrin as being a dominant mechanism. The authors may not agree with this perspective in the field (which is fine) but they should nevertheless significantly re-work their language so that the link between ROS and inhibitory synapse strengthening is more balanced. As far as I understand, the link between ROS and the glycine receptor has not been studied so this should be noted too.

Introduction

1. Lines 33-34 "….which was attributed to the recruitment of GABA type A receptors (GABAAR) to the synapse, but a molecular mechanism explaining this phenomenon remained elusive. " This point needs clarification since I think Larson et al have provided quite some detail about the molecular mechanism.

2. Line 35. "Moreover, it is unclear, how synaptic ROS signaling would be facilitated or sustained in the reductive cytosol." The role of mitochondria and NMDARs/nNOS has been reported in the literature.

3. Line 63-65 Given that in the absence of specific functions cysteines get lost during evolution, we investigated a potential link between redox-signaling and surface cysteines going beyond the previously reported nitrosylation and palmitoylation. First line needs clarification. What do the authors mean?

4. Line 68-69 Recently, liquid-liquid phase separation (LLPS) has emerged as a novel mechanism for creating subcellular microdomains. What do the authors mean by LLPS in the context of protein-protein interactions at the synapse?

Results

1. Fig 1A, not sure of the quantification of WB reflects what is observed. The example blot from whole brain seems reasonable but the spinal cord and particularly the liver blot seems quite weak. Since the focus of the study is on neuronal tissue, it would make more sense to focus on neuronal tissue.

2. Figure 2: Based on the biochemistry data in figure 1 and distribution data in figure 2, shouldn't the authors expect to observe two populations of gephyrin (monomeric and multimeric). Given this, can the authors conclude that the small gephyrin clusters seen in the C212/284S, o212/284 and cys-free data correspond to mainly monomeric gephyrin? Given this, couldn't they show the observation sin Fig 1a by looking at the biochemical properties of gephyrin (monomeric vs multimeric) in hippocampal neurons?

3. Labelling of the y-axis of figure 5d and e, I think is incorrect. Shouldn't it read D-amino oxidase punctae and not gephyrin punctae?

4. The term liquid-liquid phase separation needs a better explanation for what this means to the general reader.

Language

1. Lines 106-108: The sentence needs a verb and "Crystall" has only one "l"

2. "Crystall structures of G- and E-domain25,28, as well as the identification of palmitoylation19, assigned cysteines at position 26, 154, 212, 284, 419, 469 and 633 as surface-exposed (Figure 1 B and C). "

3. Line 112: "unordered" might be better to use disordered

4. Lines 116-119: It is not clear to me what the authors mean here. What measurement or property are they referring to?

5. "These variants were expressed in cultured primary hippocampal neurons, and the synaptic localization of gephyrin was analyzed. Except for the 6 palmitoylated Cys212 and 284, none of the other substitutions resulted in any noticeable effect."

6. Line 244, Define D-Met.

Reference List

1. References 3 and 26 in the Reference List, Accardi et al (2014) are the same study. This oversight should be corrected.

2. References 1 and 40 in the Reference List, Halliwell (1992) JNeurochem

3. References 4 and 52 in the Reference List, Esposito et al (2006) JNS

4. References 5 and 51 in the Reference List, Gutteridge et al (1993) Free Radical Research Communication

Reviewer #3

(Remarks to the Author)
Overall this is an interesting and important paper. It strengthens the emerging concept that the clustering of neurotransmitter receptors is controlled by reversible oxidation-reduction processes, thus translating changes in metabolism into dynamic modulation of neuronal signal transmission.

I believe that the paper can be further improved, by providing additional explanations/clarifications and also by including some additional experiments.

Fig. 1:

Did the authors do the perfusion experiment also in the absence of NEM (PBS only)? If yes, this should be shown as well. NEM is not only used to prevent artificial thiol oxidation, but also to prevent artificial disulfide reduction (both is possible when cells are lysed, depending on the protein and its location). If the multimer fraction increases with NEM, it implies that gephyrin disulfides are prone to reduction. If the multimer fraction decreases with NEM, it implies that gephyrin thiols are prone to artificial oxidation, raising the question if enough NEM has been used.

This should be clarified by doing the same analysis (with and without NEM) on cells in culture. It seems that a corresponding analysis (i.e., demonstrating covalent multimers on a gel) has not been performed on cultured cells.

Can the multimers be seen in lysates from cell lines or primary cultures? To what extent is gephyrin oxidation cell-type specific? Is it also seen in HeLa or HEK cells?

Was it expected to see gephyrin multimers in the liver?

If you transfect tagged gephyrin into cells, does it form covalent conjugates? (In Fig. S4 tagged gephyrin is ectopically expressed in neurons. Why not demonstrate the presence of the high molecular weight conjugates on a non-reducing gel?)

Fig. 1B

I recommend to also include the four buried cysteines in the scheme. Otherwise a swift reader may think that Fig 1B shows all the cysteines that exist. The buried cysteines are important for the interpretation of the experiment shown in 1E.

Fig. 1C

The structures shown in 1C raise the question of how the exposed cysteines relate to each other in a hexagonal lattice model. Which cysteines come into proximity at the triple G domain contact points or the paired E domain contact points? Based on known structures and predictions (AlphaFold?) are there obvious candidates for disulfide bonds that would fit the lattice? This should be clarified and discussed.

Fig. 1D

It is said that single cysteine replacements did not influence synaptic localization, but no data is shown. I would recommend to show these results in the supplement.

Most obviously (in my opinion), the influence of the single Cys replacements on the formation of multimer bands should be evaluated on non-reducing gels with high resolution.

Does removal of the palmitoylation sites (C212/284S) prevent oxidative oligomerization? If yes, this would imply that gephyrin needs to be recruited to the inner face of the plasma membrane in order to be oxidized into covalent multimers.

Assuming that single Cys mutations have no influence on the formation of the multimer(s), what about double or triple cysteine replacements? Those may well show informative phenotypes. For example, what happens to the covalent conjugates if you remove the two G domain cysteines, or the three E-domain cysteines?

I find it unfortunate that the mutant lacking all exposed cysteines is called “Cys-free”, although it still contains the buried cysteines. Perhaps a better nomenclature can be found?

Fig. 1E

The labelling of the gels (minimum/maximum/steady state) is somewhat confusing; it would be much clearer to refer to the actual treatments (-PEG/-SDS; +PEG/+SDS; +PEG/-SDS).

Fig. 3A

As expected, the strong oxidant diamide crosslinks accessible thiols into disulfides, thus generating higher order covalent aggregates. This experiment confirms the notion that gephyrin has surface exposed thiols. I am not sure what else can be concluded here about gephyrin. Wouldn’t one expect that any protein with multiple exposed thiols would show the same result? Please clarify.
Do the authors believe/assume that gephyrin is directly oxidized by H_2O_2 in cellulo? In that case it would be logical to use H_2O_2 rather than the non-natural (and much stronger) thiol oxidant diamide. How much H_2O_2 do you need to get the same degree of aggregation as with diamide?

Fig. 4A/B

Naturally, the oxidized (disulfide-crosslinked) gephyrin likes to sediment (pulling the interacting pentameric receptor with it). Does this qualify as a demonstration of phase separation? Please justify.

Figs. 5/6

It is nice to see that endogenous generation of H_2O_2, directly (DAO) or indirectly (complex III inhibition) impacts gephyrin and its interaction at the synapse. I am just wondering if the amount of H_2O_2 produced under these circumstances can be considered close to physiological. Please clarify.
Would it be possible to also demonstrate in these experiments the formation of covalent oligomers on a gel (ideally by titrating the L-aa or the ETC inhibitor) ?

When cells are treated with H_2O_2 from outside, does this induce/increase gephyrin crosslinking? How much H_2O_2 do you need in this case? Do you need mM concentrations, or just low micromolar concentrations?

It would be great if one could demonstrate the relationship between gephyrin thiol oxidation state and its clustering in cells undergoing physiological changes (e.g. a change in metabolism or activation/stimulation).

Fig. S3C

In the text (lines 127-129) it has been said that for all variants “we observed similar… functional enzymatic activity”, but in the

figure, the cys-free variant has significantly reduced Moco activity.

Later in the text (line 150) it is finally stated that the cys-free variant is significantly impaired, but now there is no reference to the figure.

The context of these experiments may need some explanation. Why is Moco activity tested? Is it relevant for the scaffolding function?

Fig. S5A/B

I have trouble understanding these gels. S5A: What is the prominent HMW band in lane 2 (peak 2 of reduced wt)? S5B: Why are there multimer band in wt-rd-2? And why are there multimer bands in cys-free-ox-2? Why is it stated that there is no difference between the oxidized/reduced sample of cys-free gephyrin (line 175)? Please clarify.

General comments:

The conclusions of the paper should be phrased more carefully.

The abstract states that mitochondria-derived ROS were identified as a physiological source of gephyrin oxidation. The antimycin experiment suggests that mitochondria can (in principle) trigger gephyrin oxidation, but how do we know that mitochondrial oxidants are driving gephyrin oxidation under physiological conditions?

There is a lot of emphasis on mentioning/implicating LLPS, but little data relating to this aspect. I would recommend to tone this down.

The actual mechanism of gephyrin oxidation seems to be beyond the scope of the current manuscript, but should at least be briefly discussed. For example, it is not obvious that gephyrin is directly oxidized by H2O2 (although that is a possibility). Alternatively, oxidation may be caused by another oxidant species, potentially derived from H2O2, or through mediation by a redox enzyme (oxidoreductase, peroxidase).

Version 1:

Reviewer comments:

Reviewer #1

(Remarks to the Author)
The authors have added new results and substantially broadened the scope of the study. All my concerns have been addressed. I have no further reservations.

Reviewer #3

(Remarks to the Author)
The manuscript has been improved, and many of the previously raised questions have been further clarified. I am generally pleased to see this study moving toward publication.

However, from my perspective, the redox biochemistry of gephyrin within cells remains rather ill-defined. The formation of disulfide-linked oligomers is demonstrated exclusively in NEM-perfused brain tissue, but not in any of the cell-based experiments.

In their response, the authors note that they were unfortunately unable to detect covalent gephyrin multimers in their cultured cells. I find this both puzzling and concerning. I do not believe this point should be treated as a minor or easily explained detail.

All cell-based experiments (cluster analysis, patch clamp; with and without additional H_2O_2 generation by DAO) are built on the premise that gephyrin forms intermolecular disulfide bonds. Based on this, one would reasonably expect it to be possible (using appropriate alkylation and extraction protocols) to demonstrate a correlation between gephyrin disulfide crosslinking and the observed phenotypes, such as changes in cluster size. In my view, this remains a gap in the argument of the study.

In their rebuttal, the authors propose that in brain tissue obtained from adult animals, disulfide bond formation may be favored, whereas in cultured hippocampal neurons, sulfenylation could predominate. However, it remains unclear how this would explain the observed cellular effects. Are the authors suggesting that sulfenylation promotes non-covalent gephyrin oligomerization such that covalent crosslinks are not necessary? Or that sulfenylation bypasses the need for oligomerization altogether? Both interpretations seem unlikely and are, in any case, at odds with the central narrative of the paper, which begins with the observation of covalent gephyrin oligomers in brain extracts and then demonstrates disulfide-linked multimers in vitro. The manuscript clearly leads the reader to the conclusion that thiol oxidation results in disulfide formation,

which stabilizes gephyrin oligomers at the membrane.

As far as I can tell, it is not clearly stated in the manuscript that covalent gephyrin conjugates could not be detected in the cellular models used. Instead, the authors employ the more general term “thiol oxidation” in place of “disulfide bond formation,” to reflect the absence of direct evidence for disulfides in cells. I believe this issue should be addressed more explicitly and transparently in the discussion.

Version 2:

Reviewer comments:

Reviewer #3

(Remarks to the Author)
The authors have improved the manuscript by explicitly acknowledging the gaps in their understanding of gephyrin redox biochemistry and by adopting a more cautious interpretation of their findings. I consider the revised manuscript suitable for publication and would be pleased to see it published.

Open Access This Peer Review File is licensed under a Creative Commons Attribution 4.0 International License, which permits use, sharing, adaptation, distribution and reproduction in any medium or format, as long as you give appropriate credit to the original author(s) and the source, provide a link to the Creative Commons license, and indicate if changes were made.
In cases where reviewers are anonymous, credit should be given to 'Anonymous Referee' and the source.
The images or other third party material in this Peer Review File are included in the article’s Creative Commons license, unless indicated otherwise in a credit line to the material. If material is not included in the article’s Creative Commons license and your intended use is not permitted by statutory regulation or exceeds the permitted use, you will need to obtain permission directly from the copyright holder.
To view a copy of this license, visit https://creativecommons.org/licenses/by/4.0/

Reviewer #1

In this manuscript, Gehling et al., build on the earlier work from the same laboratory and determine the role of mitochondrial ROS on gephyrin clustering. Using a range of in vitro biochemical assays they describe the contribution of several surface-exposed cysteine residues for ROS-induced phase separation, increased receptor binding, and reduced proteolytic cleavage by calpain1. Using primary neurons treated with antimycin the authors link mROS to gephyrin clustering at GABAergic synaptic sites and using DTT they show the reversible nature of gephyrin clustering changes in response to ROS. Interestingly, the authors demonstrate that MoCo function of gephyrin is independent of oxidative status.

The experiments outlined are well performed with relevant statistical tests. The results described are original and relevant to the field as ROS-induced neuronal cell death is a leading hypothesis for healthy aging and neurodegeneration. A major caveat of this study is the in vitro nature of all the data presented in vitro, the timeline of responses in their study and in vivo remains unclear.

Major clarifications are required to be consistent with published literature on this topic.

1. Accardi et al., which has been cited by the authors reported mROS effects within 5 min of mROS induction in acute slices. Importantly, the abundant a1 containing GABAARs did not respond to mROS, while the less abundant a3 containing GABAARs were recruited within the 5-minute time frame to increase GABAergic amplitude and frequency after ROS. The mechanistic basis for this GABAAR subunit selectivity is not clear from the current data.

ANSWER:

Accardi et al. observed the recruitment of α3-containing $GABA_A$Rs upon mROS induction in cerebellar stellate cells. In a follow up study from the same laboratory, Larson et al. demonstrate that PKC promotes the selective strengthening of α3-containing $GABA_A$Rs in a GABARAP-dependent and gephyrin-independent manner. The authors speculate that superoxide leads to PKC activation. The pathway uncovered by the later study does not require ROS generation by mitochondria. However, Larson et al. demonstrate that Antimycin A treatment can also trigger the PKC-dependent pathway, while under physiological conditions the recruitment of α3 containing $GABA_A$Rs is otherwise downstream of Nox2 signaling.

We conclude that Antimycin A treatment is not a good method to distinguish between mitochondrial and Nox2-derived ROS. In order to clarify our current findings on gephyrin-dependent $GABA_A$R clustering and to avoid that the reader may conclude that only mitochondria-derived ROS may play a role, we decided to remove the Antimycin A treatment from the current manuscript and discuss that multiple ROS sources, such as mitochondria and Nox2 could drive this mechanism.

Additionally, all of our experiments conducted in dissociated hippocampal neurons indicate that ROS-induced gephyrin-dependent $GABA_A$R clustering is different from GABARAP-dependent (and gephyrin-independent) recruitment of α3-containing $GABA_A$Rs described by Accardi et al. and Larson et al. For example, we demonstrate that the size of synaptically localized endogenous gephyrin clusters increased in the presence of H_2O_2, derived from D-methionine in the presence of active D-amino oxidase (Figure 5C-E). Furthermore, overexpressed gephyrin behaved similarly only when its surface-exposed cysteines were present. Our novel functional data, i.e. mIPSC recordings (see a more detailed description below), indicate that gephyrin without its surface exposed cysteines resulted in significantly increased rise times (new Figure 6B and Supplementary Figure 8). In contrast, mIPSC rise

times are significantly decrease in the presence of intracellular H_2O_2 (new Figure 6D and Supplementary Figure 8). Under these conditions we observed larger mIPSC amplitudes and increased frequencies (Supplementary Figure 8I,N). While the increased frequency could point towards recruitment of α3 containing $GABA_A$Rs (Accardi et al. and Larson et al.), the larger amplitudes are distinct and align with an increased amount of gephyrin scaffolding protein, resulting in an increased number of overall $GABA_A$R binding sites.

Additionally, as outlined below, we observe that phase separation of gephyrin with either α1- or α3-model proteins is increased under oxidizing conditions. Collectively, our data highlight a distinct new mechanism of inhibitory synapse strengthening in hippocampal neurons, which would not discriminate between α1- and α3-containing containing $GABA_A$Rs.

We added a section to the discussion to clarify:

"While prior studies have demonstrated that inhibitory synapses in molecular layer interneurons are strengthened upon ROS-induction through the recruitment of α3-containing $GABA_A$Rs in a gephyrin-independent manner[3,4], our findings in hippocampal neurons suggest the existence of an additional gephyrin-dependent mechanism. Interestingly, in this gephyrin-dependent mechanism, oxidation resulted in fast mIPSC rise times, which may reflect increased temporal precision of synaptic inhibition. Additionally, gephyrin oxidation in LLPS experiments suggests that both α1- and α3-containing $GABA_A$Rs may be recruited by this mechanism."

2. Phase separation drives the compartmentalization of intracellular contents into various biomolecular condensates. Authors cite work on gephyrin phase separation from Bai et al, 2021, however, in this study Bai et al., reported that phase separation of GlyR and gephyrin is sensitive to molar ratios and salt concentration. Also, gephyrin/a3 GABAAR subunit LD complex phase separation occurred at 5microM conc and was concentration dependent. Importantly, a1LD/ gephyrin phase separation did not follow this rule. This aspect of the subunit differences in mROS-mediated gephyrin phase separation would have clarified why less abundant a3 GABAARs are recruited to inhibitory synaptic sites after mROS in neurons.

ANSWER:

We thank the reviewer for pointing out these differences in phase separation behavior between α1- and α3-model systems. We conducted new experiments, using the published GCN4-model from Bai et al. for GlyR β, $GABA_A$R α1 and $GABA_A$R α3 (new Figure 4A,B and Supplementary Figure S7). Using these model systems, we found that oxidation increases gephyrin phase separation together with $GABA_A$R α1 and with $GABA_A$R α3. GCN4 GlyR β undergoes strong phase separation with gephyrin already under reducing conditions. This behavior was not further enhanced under oxidizing conditions. We suspect that the strong phase separation ability of GCN4 GlyR together with gephyrin may occlude the observation of additional effects due to oxidation. Since the GCN4 model was designed for its phase separation behavior, we now only present data using this published GCN4 system and removed data using our yet not published lumazine synthase GlyR β-ICD model. Importantly, as outlined above, phase separation of gephyrin with GCN4 $GABA_A$R α1 and $GABA_A$R α3 was enhanced under oxidizing conditions. These findings indicate that gephyrin would not discriminate between α1- or α3-containing $GABA_A$Rs. In line with these findings, ROS-induced gephyrin-dependent strengthening of inhibitory synapses seems distinct from gephyrin-independent ROS-induced recruitment of α3-containing $GABA_A$Rs.

Proteins diffuse in the plane of the membrane before their accumulation at synapses, it was reported by Lee et al., 2024 that gephyrin condensates with distinct protein composition coexist

with multicomponent systems. The authors reported that C domain substitutions on gephyrin from DDX4 or G3BP1 can still induce gephyrin clustering and phase separation. IQSEC3 and NLGN2 can induce gephyrin condensation. These aspects of the results are relevant to this manuscript and should be discussed sufficiently.

ANSWER:

Lee et al. 2024 describe that "IQSEC3 tended to destabilize gephyrin phase separation" and "mild suppressive effects on gephyrin phase separation". These statements are based on co-expression experiments in HEK293T cells, using EGFP-tagged gephyrin together with client proteins such as IQSEC3. We acknowledge that in Figure 6E, Lee et al. 2024 report a mild increase in the gephyrin saturation concentration in the presence of IQSEC3 compared with the condition of "no client". Overall, no statistical test was performed (or reported) and it is difficult to judge whether specifically IQSEC3 (co-transfection) has an effect on the gephyrin saturation concentration compared to the "no client" condition (single transfection). Therefore, it needs to be taken with caution if the reviewer states that "IQSEC3 and NLGN2 can induce gephyrin condensation". In light of the preliminary nature and lack of control experiments, we decided not to mention a potential effect of IQSEC3 on gephyrin phase separation in our manuscript.

In contrast, Lee et al.2024 convincingly demonstrate that NLGN2 can promote the nucleation of gephyrin condensates. As suggested by the reviewer, we added a section to the discussion to discuss the possible interplay between gephyrin oxidation and nucleation with regards to potential implications in synapse formation:

"Neuroligin 2 (NLGN2) is a post-synaptic cell-adhesion molecule that can interact with pre-synaptic Neurexins and is exclusively found at GABAergic synapses[47]. Local NLGN2 accumulation can promote the nucleation of gephyrin condensates[31]. Our data demonstrate that gephyrin oxidation increased condensation with $GABA_AR$ model proteins, and future work could test whether oxidation could influence gephyrin nucleation (for example locally at NLGN2 contact sites). While our current study suggests that existing GABAergic synapses are strengthened by this process, work on gephyrin nucleation could be instructive as to whether the formation of new synapses could be promoted.“

3. Timeline of hydrogen peroxide, antimycin, D-MET, DTT treatments is not clear. Also, it is not clear at what time point after the treatment the primary neurons were fixed and analyzed.

ANSWER:

We thank the reviewer for pointing out, that these timelines were not clear. We now clarified the timeline directly in the respective figure legends.

4. Acute hippocampal slices expressing gephyrin cysteine mutants with intracellular antimycin A application for mROS effects would be very convincing in vivo results to support all the in vitro data.

ANSWER:

We agree with this reviewer that convincing in vivo results could strengthen the in vitro data in our manuscript. However, to obtain "acute hippocampal slices expressing gephyrin cysteine mutants", would require a substantial amount of in vivo work (i.e. generation of novel transgenic mouse lines, knock-in mouse lines or stereotactic surgeries with virus injections) that would be

beyond the scope of this manuscript. We feel confident that our novel functional data support the overall findings of our manuscript and we added a paragraph on limitations (especially the use of in vitro model systems) to the discussion of our manuscript.

“A limitation of our study is the use of dissociated hippocampal cultures, which do not maintain their correct anatomic input-specificity.”

****

Reviewer #2

Gehling and colleagues perform a comprehensive biochemical investigation of the role of reactive oxygen species (ROS) in triggering the multimerization of the scaffolding protein, gephyrin, and its role in clustering at inhibitory synapse. The authors’ work builds upon previous publications in the hippocampus (Penna et al, 2014) and cerebellum (Accardi et al, 2014 and 2015; Larsen et al 2020) that have shown that ROS derived from the mitochondria and/or neuronal nitric oxide synthase strengthen inhibitory synapses. Although the clustering of gephyrin has been previously linked to the strengthening of inhibitory synapses, the possibility that ROS may trigger the clustering of gephyrin has not been tested.

Critique:

The authors present a comprehensive biochemical study of the events that lead to the multimerization and eventual clustering of gephyrin. In doing so, they pinpoint mitochondrial ROS as the key trigger in these events which react with exposed cysteine residues on gephyrin to promote the formation of disulfide bounds. The study adds to previous studies identifying palmitoylation (2014 Dejanovic et al), nitrosylation (2014 Dejanovic et al) and phosphorylation (2011 Tygarajan et al) as mechanisms of posttranslational modification that control the multimerization of gephyrin. The authors’ findings are novel in not only identifying a new mechanism of gephyrin clustering but also because they fill an important gap from previous studies showing that ROS strengthens inhibitory synapses.

Despite my enthusiasm for their work, the authors, nevertheless, have several major issues to resolve to make this study appeal to the broad readership of the Journal.

First, the authors need to better articulate in the Abstract and Introduction how most researchers view the mechanisms that lead to inhibitory synapse strengthening. Most researchers currently conclude that phosphorylation of gephyrin (not disulphide bond formation) is the key step in promoting clustering and synapse strengthening. Prior work from the authors’ lab has also shown a role for palmitoylation and nitrosylation too so these mechanism need to be mentioned. The manuscript text should reflect these different mechanisms and explain why prior work prompted a new study on ROS and the crosslinking of cysteine residues on gephyrin. There are also studies showing that not all GABAR inhibitory synapses contain gephyrin so there is certainly a case to be made to why there are deficits in the gephyrin phosphorylation hypothesis. As it currently stands, the Abstract and Introduction focus only on redox-dependent regulation but would be better if a more nuanced perspective is offered.

ANSWER:

We thank the reviewer for making us aware of these text passages that need improvement. We added sections to abstract and introduction to include more broadly previously identified gephyrin PTMs in the field. We highlight in the introduction that gephyrin harbors additional surface exposed cysteine residues besides those that undergo palmitoylation or nitrosylation, which prompted our new study on ROS.

Second, most of the biochemical and imaging data in the manuscripts seems to be showing that not all gephyrin inhibitory synapses are affected by the redox state of gephyrin. For example, the clustering WT data shows population of synapses which have similar puncta size as the cys-free dataset as well as punctae which are clearly much larger. How do the authors interpret this point? Is it consistent with Accardi et al (2014) that have argued that strengthening occurs only at alpha3 and NOT alpha1 synapses? Or is it because another mechanism (e.g. palmitoylation/nitrosylation/phosphorylation) is also at play which targets a different population of gephyrin/GABAR complexes? The study would be much stronger if the authors acknowledged this variability in their dataset and offered some explanations to why it may occur. They may even consider arguing for two populations in their WT dataset.

ANSWER:

We agree with the reviewer that WT data in Figure 2 shows a high degree of variability. Indeed, this high degree of variability could result from multiple different PTMs that act in concert to regulate gephyrin clustering. As outlined above, there are a few specific points about the gephyrin-dependent mechanism uncovered in this manuscript that argue that it is different from the gephyrin-independent mechanism described by Accardi et al (2014) and Larson et al (2020) (see specific comment to reviewer 1 about different $GABA_AR$ subunits). However, the gephyrin-independent mechanism could additionally contribute to the observed high degree of variability in this dataset. We added a sentence to the results section:

Overall, the expression of EGFP-tagged wt gephyrin resulted in synaptic gephyrin puncta sizes with a high degree of variability, possibly due to a number of different PTMs that can regulate its clustering[18].

Third, the authors' biochemical and imaging work would be greatly complemented with a patch clamp electrophysiological recordings from their hippocampal neurons to show that antimycin-A (in figure 6) triggers an increase in synapse strengthening. The authors could record mini-events and plot the effect of antimycin-A over time (mIPSC amplitude and frequency) . The absence of functional data in the study greatly diminishes the potential impact and appeal of the authors' work to the broader neuroscience community.

ANSWER:

We appreciate the reviewer's input on functional data in our manuscript. We now provide two sets of whole cell patch clamp electrophysiological recording from our hippocampal neurons (new Figure 6 and Supplementary Figure S10).

In a first set of experiments, we compared neurons expressing either gephyrin wt or cys-free and observed slower mIPSC rise times for the latter. As outline above, Antimycin A treatment can result in ROS levels that can trigger pathways that may otherwise be triggered by a completely different and mitochondria-independent signaling cascade (Larson et al 2020). For this reason, we refrained from using this treatment for mIPSC recordings. Instead, comparable

to our previous experiments, we used DAAO-derived H_2O_2, to study the effect of increased intracellular ROS on mIPSCs. In agreement with our imaging data, we found ROS-induced larger mIPSC amplitudes and frequencies. While the former is compatible with our gephyrin-dependent mechanism, the latter could indicate additionally the recruitment of α3-containing $GABA_A$Rs (Accardi et al. 2014 and Larson et al. 2020). Furthermore, in these experiments we observe ROS-induced faster mIPSC rise times, which are in line with the effect of the cys-free mutant variants and support a model in which both intact gephyrin cysteine residues and increased intracellular oxidation promote faster inhibitory synaptic responses.

Fourth and finally, there are numerous formatting errors in the manuscript that include the mislabelling of figure axis to the duplication of citations in the reference list. These oversights need to be fully corrected.

ANSWER:

We thank the reviewer for making us aware of these errors and corrected those in the new version of this manuscript.

Summary of the findings:

In figure 1, the authors examine whether the redox state of cysteines are important for the oligomerization of gephyrin. Their data establishes that in tissue taken from brain, liver and spinal cord, gephyrin exists in both monomeric and multimeric gephyrin forms. In the brain, about 20% of gephyrin is multimeric in basal conditions leaving a surplus of 80% monomeric forms.

In figure 2, the authors consider the possibility that 8 exposed cysteines are the target of ROS. They observe that single point mutations in each Cys of gephyrin with a Ser residue has no effect on clustering in cultured hippocampal neurons. However, compared to the WT phenotype, mutation of all variants, that is C212/284S (palmitoylation dependent mutants), o2212/284 (two cysteines remaining) and cys-free gephyrin eliminated the ability of gephyrin to cluster.

In figure 3, the authors test the possibility that exposed cysteines form disulphide bridges that mediate synaptic clustering. Using size-exclusion chromatography, they observe high order multimers that are not observed in cys-free gephyrin. The formation of disulphide bond was confirmed by using DTT (Fig. 3a,b). The authors conclude that the mechanism of redox-dependent multimerization is due to the formation of disulphide bonds. Authors then examine the functionality of gephyrin multimers by looking at their secondary structure (Fig. 3c) and ability to bind to the glycine receptor beta subunits peptides (3d-f). The authors observed no difference in the secondary structure or binding ability of gephyrin wildtype or cys-free variants. The authors conclude that the presence or absence of disulphide bonds do not alter the secondary structure or binding ability (at least for the Glycine receptor) when existing in a monomeric or multimeric form.

In figure 4, the authors examined the potential role of the redox state of whether gephyrin in forming liquid-liquid phase separation. Using the glycine beta subunit pentamers, the authors find that oxidised WT gephyrin has a strong sedimentation properties that are absent in cys-free gephyrin (Fig. 4a-b). The authors conclude that this finding favours again that oxidised gephyrin forms high-order oligomeric clusters. The authors then look at whether the redox state is affected by calpain cleavage of gephyrin, which may be important in regulating synaptic clustering. The authors note that the ability of calpain to cleave gephyrin was reduced about

two-fold for the oxidised form compared to the reduced form (Fig 4d, left). This distinction was not observed for cys-free gephyrin (Fig 4d, right). The authors also show that the remaining undigested gephyrin shows resistance to calpain digestion that was not observed in the cys-free gephyrin (Fig. 4e). Together, the authors conclude that the structural properties of gephyrin are affected by its redox state (i.e. phase separation and proteolytic sensitivity).

In figure 5, the authors examined whether the release of cytosolic hydrogen peroxide affects the clustering of endogenous (Fig. 5a) and recombinant gephyrin (wt and cys-free, Fig. 5b). To do this, the authors expressed D-amino oxidase in hippocampal neurons, which generates hydrogen peroxide when exposed to D-Met. Synaptic clustering of gephyrin was assessed by co-localization with the presynaptic marker, V-GAT. The authors report that D-amino acid oxidase and gephyrin clustering is increased by about 20 and 14 % respectively (Fig 5g-h). I believe that the y-axis labelling of Fig 5 d and e is incorrect so there was some confusion between the information in the text and figures. It was also unclear why the intensity of mEGFP-tagged gephyrin decreased following D-Met treatment. Some explanation is required. The authors conclude that oxidation of gephyrin with cytosolic hydrogen peroxide promotes clustering of gephyrin.

ANSWER:

We thank the reviewer for pointing out these discrepancies. D-amino oxidase was cytosolically expressed and did not cluster in neurons. We analyzed gephyrin clustering, D-amino oxidase localization was not analyzed.

In Figure 5 F-H, we analyzed the effects of D-Met treatment on cells that co-expressed either D-amino oxidase with mEGFP-gephyrin wt or D-amino oxidase with mEGFP-gephyrin cys-free. Our finding that mEGFP-tagged gephyrin cluster intensities decreased in the presence of D-Met could stem from fluorophore bleaching. Increased ROS levels resulted in larger mEGFP-gephyrin wt clusters, which could be shielded from the reductive cytosol. Inside those clusters, mEGFP may display an increased propensity to form non-native disulfide bonds and fail to fluoresce (Constantini et al. NatCommun 2015). In contrast, increased ROS levels did not affect the size of mEGFP-gephyrin cys-free clusters and clusters may be similarly exposed to the reductive cytosol.

We added a sentence to the results section to offer a possible explanation:

"This effect may originate from disulfide bond formation and/or impacts on mEGFP folding in oxidized mEGFP-gephyrin wt clusters that may be shielded from the reductive cytosol[36]."

Finally, in figure 6, the authors repeat the experiments of figure 5 but elevate reactive oxygen species (ROS) using antimycin A which uncouples the ability of mitochondria to generate ATP through proton transport allowing molecular oxygen to react with protons to form ROS. Antimycin treatment caused a 13% increase in cluster size and 25% increase in intensity of gephyrin staining (Fig. 6d and e) that was reversed by treatment with DTT.

Specific Points:

Abstract. The narrative of the abstract needs to be toned down or at least more carefully articulated. The predominant view is that GABAR inhibitory strengthening is mediated by the actions of kinases that are activated by elevations in cytosolic Ca^{2+} (e.g. 2020, Barberis). The observations establishing a link between ROS and inhibitory strengthening is distinct in this

regard having been shown in molecular layer interneurons (Accardi et al, Nat Comm; Larsen et al JNS), granule cells (Accardi et al, JNS) and hippocampal neurons (Penna et al). I appreciate that the Schwarz lab has added to this perspective significantly through biochemical studies but the rest of the field is still focused on the phosphorylation of gephyrin as being a dominant mechanism. The authors may not agree with this perspective in the field (which is fine) but they should nevertheless significantly re-work their language so that the link between ROS and inhibitory synapse strengthening is more balanced. As far as I understand, the link between ROS and the glycine receptor has not been studied so this should be noted too.

ANSWER:

We have re-worked the abstract and mention various gephyrin PTMs that may act in concert to regulate inhibitory synapse structure and function. With our new experiments using glycine and $GABA_AR$ receptor models (see specific comment from reviewer 1), we provide an experiment that suggest that LLPS with glycine receptor models may not be sensitive to oxidation. Additionally, we note in the discussion that the influence of ROS on glycinergic synapses has not been studied.

Introduction

1. Lines 33-34 "….which was attributed to the recruitment of GABA type A receptors (GABAAR) to the synapse, but a molecular mechanism explaining this phenomenon remained elusive. " This point needs clarification since I think Larson et al have provided quite some detail about the molecular mechanism.

ANSWER:

We agree with the reviewer that Larson et al have provided extensive detail about the mechanism. Therefore, we have removed the second part of our sentence. With our new electrophysiological data, it became clear that we are looking here at two distinct mechanisms and we acknowledge these now as such in the manuscript.

2. Line 35. "Moreover, it is unclear, how synaptic ROS signaling would be facilitated or sustained in the reductive cytosol." The role of mitochondria and NMDARs/nNOS has been reported in the literature.

ANSWER:

We agree with the reviewer and replaced the sentence with a sentence that describes the NMDAR/nNOS axis. As outlined above, mitochondria derived ROS can trigger this pathway, however this was solely based on Antimycin A treatment and may not occur under physiological conditions (Larson et al. 2020).

3. Line 63-65 Given that in the absence of specific functions cysteines get lost during evolution, we investigated a potential link between redox-signaling and surface cysteines going beyond the previously reported nitrosylation and palmitoylation. First line needs clarification. What do the authors mean?

ANSWER:

Our intention was to state that the conservation pattern of surface cysteines is influenced by their function within proteins (Marino & Gladyshev VN. J Mol Biol. 2010), however we decided to remove this statement to avoid the impression of overinterpretation.

4. Line 68-69 Recently, liquid-liquid phase separation (LLPS) has emerged as a novel mechanism for creating subcellular microdomains. What do the authors mean by LLPS in the context of protein-protein interactions at the synapse?

ANSWER:

As proposed by several studies (reviewed in Chen et al. 2020), we state here that dense molecular clusters at pre- and postsynaptic sites could be formed via biomolecular condensate formation or LLPS. To clarify, we changed the sentence to:

"Recently, liquid-liquid phase separation (LLPS) has emerged as a novel mechanism for creating subcellular microdomains and has been proposed as a possible mechanism for the formation of protein assemblies at presynaptic terminals and postsynaptic densities[26]."

Results

1. Fig 1A, not sure of the quantification of WB reflects what is observed. The example blot from whole brain seems reasonable but the spinal cord and particularly the liver blot seems quite weak. Since the focus of the study is on neuronal tissue, it would make more sense to focus on neuronal tissue.

ANSWER:

We agree with the reviewer that the focus is neuronal tissue and we have, as suggested, removed the analyses of spinal cord and liver.

2. Figure 2: Based on the biochemistry data in figure 1 and distribution data in figure 2, shouldn't the authors expect to observe two populations of gephyrin (monomeric and multimeric). Given this, can the authors conclude that the small gephyrin clusters seen in the C212/284S, o212/284 and cys-free data correspond to mainly monomeric gephyrin? Given this, couldn't they show the observations in Fig 1a by looking at the biochemical properties of gephyrin (monomeric vs multimeric) in hippocampal neurons?

ANSWER:

Western blot analysis in Figure 1 using SDS-PAGE (denaturing conditions) demonstrates the presence of covalently (DTT-labile) linked species. However, non-covalent linkages cannot be revealed in this experiment. Additionally, gephyrin is a cytosolic protein and only a fraction is localized at inhibitory synapses. Under native conditions a minimum of three gephyrin monomers are non-covalently linked and higher order oligomers have been described as well. In the synaptic cluster analysis (Figure 2) fluorescent punctae are formed with an estimated density of 5,000 – 10,000 gephyrin molecules per µm^2 (Specht et al. 2013).

3. Labelling of the y-axis of figure 5d and e, I think is incorrect. Shouldn't it read D-amino oxidase punctae and not gephyrin punctae?

ANSWER:

In Figure 5 we have analyzed gephyrin punctae in the presence of DAAO. The readout for quantitative analyses were either endogenous gephyrin punctae in Figures 5 D,E (revealed with antibody staining) or mEGFP puncate in Figure 5 G,H (from mEGFP-tagged wt or cys-free gephyrin expressed via recombinant AAVs). DAAO displayed a diffuse cytosolic pattern and did not show punctae.

4. The term liquid-liquid phase separation needs a better explanation for what this means to the general reader.

ANSWER:

We thank the reviewer and expanded on the respective section in the introduction. The paragraph is now:

"Several proteins at excitatory postsynaptic densities can undergo LLPS[27,28] and gephyrin together with model proteins of GlyR/$GABA_A$Rs can be organize into concentrated, droplet-like compartments—without the need for membranes[29]. Additionally, proteins at excitatory and inhibitory postsynaptic densities spontaneously segregate into distinct condensed molecular assemblies[30]. Importantly, gephyrin condensation is critically regulated by its central intrinsically disordered region (IDR) linker through conformational collapse and electrostatic interdomain interactions, rather than IDR–IDR interactions, with other iPSD components modulating its stability and localization via a thermodynamically driven network of intermolecular interactions[31]. In addition, the ability of gephyrin to undergo LLPS may be influenced by redox-changes or may impact gephyrin's accessibility to redox signals."

Language

1. Lines 106-108: The sentence needs a verb and "Crystall" has only one "l":

2. "Crystall structures of G- and E-domain25,28, as well as the identification of palmitoylation19, assigned cysteines at position 26, 154, 212, 284, 419, 469 and 633 as surface-exposed (Figure 1 B and C). "

3. Line 112: "unordered" might be better to use disordered

ANSWER:

We thank the reviewer and made these adjustments to the text.

4. Lines 116-119: It is not clear to me what the authors mean here. What measurement or property are they referring to? : 5. "These variants were expressed in cultured primary hippocampal neurons, and the synaptic localization of gephyrin was analyzed. Except for the 6 palmitoylated Cys212 and 284, none of the other substitutions resulted in any noticeable effect."

ANSWER:

Here, we are referring to previously published data (Dejanovic et al, 2014). To clarify, we changed the paragraph to:

“Previously, Cys212 and 284 were identified as targets of palmitoylation[25]. In this study, each of the other eight surface cysteines was individually substituted with serine[25]. Overall, except for the palmitoylated Cys212 and 284, none of the other substitutions resulted in any noticeable effect on gephyrin cluster size, which suggested that they were individually dispensable[25].”

6. Line 244, Define D-Met.

ANSWER:

We changed it to D-methionine (D-Met).

Reference List

1. References 3 and 26 in the Reference List, Accardi et al (2014) are the same study. This oversight should be corrected.

2. References 1 and 40 in the Reference List, Halliwell (1992) JNeurochem

3. References 4 and 52 in the Reference List, Esposito et al (2006) JNS

4. References 5 and 51 in the Reference List, Gutteridge et al (1993) Free Radical Research Communication

ANSWER:

We thank the reviewer and made these important adjustments.

****

Reviewer #3

Overall this is an interesting and important paper. It strengthens the emerging concept that the clustering of neurotransmitter receptors is controlled by reversible oxidation-reduction processes, thus translating changes in metabolism into dynamic modulation of neuronal signal transmission.

I believe that the paper can be further improved, by providing additional explanations/clarifications and also by including some additional experiments.

Fig. 1:

Did the authors do the perfusion experiment also in the absence of NEM (PBS only)? If yes, this should be shown as well. NEM is not only used to prevent artificial thiol oxidation, but also to prevent artificial disulfide reduction (both is possible when cells are lysed, depending on the protein and its location). If the multimer fraction increases with NEM, it implies that gephyrin disulfides are prone to reduction. If the multimer fraction decreases with NEM, it implies that gephyrin thiols are prone to artificial oxidation, raising the question if enough NEM has been used.

ANSWER:

We thank the reviewer, for this important comment. For initial experiments, we did indeed perform perfusion in the absence of NEM, split the brain homogenate in two and incubated one sample with and the other sample without NEM (see image below).

We find in the complete absence of NEM (second lane from the left) a smear above the monomeric gephyrin species. Under these conditions even GAPDH displayed a weaker signal with a double band. Adding NEM to the homogenate displayed two different gephyrin oligomeric species and again a smear in the lane. The smear and oligomeric species were sensitive to reducing agent (in this experiment β-mercaptoethanol, β-ME).

In our interpretation, these results demonstrate that NEM, indeed blocks free thiols and prevents artificial disulfide bond formation. When comparing these results to NEM perfusion (Figure 1A), the benefits of NEM treatment become clear (reduction of smear).

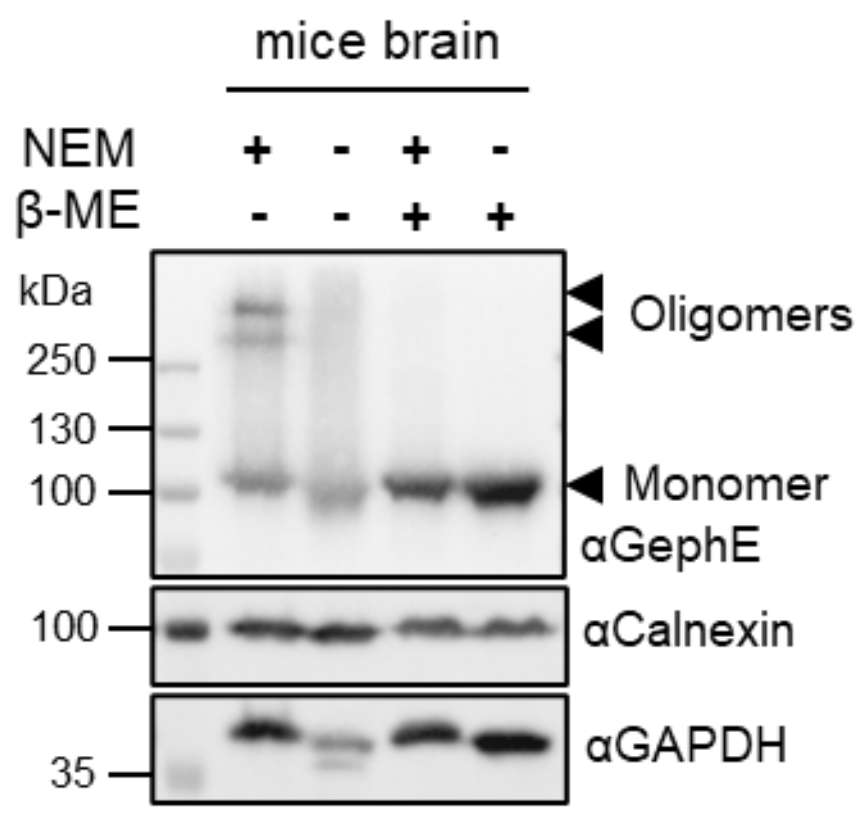

This should be clarified by doing the same analysis (with and without NEM) on cells in culture. It seems that a corresponding analysis (i.e., demonstrating covalent multimers on a gel) has not been performed on cultured cells.

Can the multimers be seen in lysates from cell lines or primary cultures? To what extent is gephyrin oxidation cell-type specific? Is it also seen in HeLa or HEK cells?

ANSWER:

We agree with the reviewer that additional analyses of multimers derived from cultured cells could add to our analyses. However, as outlined below (see reviewer's point on specific cysteines), we did not convincingly observe gephyrin multimers from cultured cells.

Was it expected to see gephyrin multimers in the liver?

ANSWER:

We performed these experiments with an open mind, since prior studies from our laboratory have revealed that neuronal gephyrin isoforms can perform metabolic functions (Smolinsky et al. JBC 2008). The absence of the protein machinery that is required for the formation of inhibitory postsynaptic densities is likely responsible for the absence of detectable gephyrin clusters in non-neuronal cells. Furthermore, gephyrin cytosolic extracts from adult rat liver or

brain migrates at ~600 kDa as analyzed by blue-native PAGE (Nawrotzki, et al. Histochemistry and Cell Biology 2012). Overall, these results indicate that neuronal and non-neuronal gephyrin display similar biochemical behavior and could suggest that a certain repertoire of similar regulatory mechanisms could exist for both forms of gephyrin and organs present.

The major difference between the predominant neuronal isoform and the gephyrin isoform in liver is the C3 cassette, which adds 36 residues to the gephyrin C-domain and does not contain cysteine residues. Surface exposed cysteines should therefore be comparable between neuronally expressed gephyrin P1 and gephyrin C3 expressed in the liver. ROS in hepatic cells could affect gephyrin C3 in a similar fashion as gephyrin P1 in neurons. Given that in the liver gephyrin is part of the molybdenum cofactor synthetic machinery, condensed states of gephyrin may also be beneficial to orchestrate the biosynthesis and minimize degradation of the oxidation-sensitive cofactor.

If you transfect tagged gephyrin into cells, does it form covalent conjugates? (In Fig. S4 tagged gephyrin is ectopically expressed in neurons. Why not demonstrate the presence of the high molecular weight conjugates on a non-reducing gel?)

ANSWER:

We could not observe high molecular weight conjugates on a non-reducing gel from primary neuron cultures. We postulate that in brain tissue that we obtained from adult animals disulfide bond formation is favored, whereas in cultured hippocampal cells S-sulfenylation as transient forms of cysteine oxidation could be predominant. We refer to this form of cysteine modification at the end of the discussion. To clarify, we made sure that we do not refer to disulfide bonds when referring to the results obtained from cultured cells.

Fig. 1B

I recommend to also include the four buried cysteines in the scheme. Otherwise a swift reader may think that Fig 1B shows all the cysteines that exist. The buried cysteines are important for the interpretation of the experiment shown in 1E.

ANSWER:

We now included the four internal cysteine residues in Fig 1B.

Fig. 1C

The structures shown in 1C raise the question of how the exposed cysteines relate to each other in a hexagonal lattice model. Which cysteines come into proximity at the triple G domain contact points or the paired E domain contact points? Based on known structures and predictions (AlphaFold?) are there obvious candidates for disulfide bonds that would fit the lattice? This should be clarified and discussed.

ANSWER

Interestingly, in an independent study from our laboratory using high-resolution Cryo-EM (currently under review, see also https://www.researchsquare.com/article/rs-5889959/v1), we find Cys419 in gephyrin's E-domain in proximity to the adjacent Cys419 in a novel Z-shaped

interface between E-domain dimers. Future experiments in our laboratory will explore the specific role of this residue but a detailed analysis is beyond the role of this manuscript.

Fig. 1D

It is said that single cysteine replacements did not influence synaptic localization, but no data is shown. I would recommend to show these results in the supplement.

ANSWER

Here, we are referring to previously published data (Dejanovic et al, 2014), we made this more clear in the manuscript, see also specific point (Language) of reviewer #2.

Most obviously (in my opinion), the influence of the single Cys replacements on the formation of multimer bands should be evaluated on non-reducing gels with high resolution.

ANSWER

As mentioned above, a detailed structural and functional analysis of individual cysteine replacement variants is currently underway in our laboratory and is beyond the scope of this manuscript focusing on the initial discovery of gephyrin redox regulation. However, further below we share new data using domain-specific replacements of cysteine residues and analyses of high oligomers in non-reducing gels.

Does removal of the palmitoylation sites (C212/284S) prevent oxidative oligomerization? If yes, this would imply that gephyrin needs to be recruited to the inner face of the plasma membrane in order to be oxidized into covalent multimers.

ANSWER

This hypothesis put forward by the reviewer is very intriguing and it is likely that membrane targeting is required for oxidative oligomerization. In contrast to PSD-95, which is palmitoylated to a high degree, gephyrin palmitoylation has been estimated to be roughly 4% (Dejanovic et al, 2014). In addition to palmitoylation, interaction with postsynaptic proteins can recruit gephyrin to the membrane. Therefore, in the cellular model systems available to us, gephyrin C212/284S will still be recruited to inhibitory synapses (to a lower degee) and oxidative oligomerization is therefore still likely.

Assuming that single Cys mutations have no influence on the formation of the multimer(s), what about double or triple cysteine replacements? Those may well show informative phenotypes. For example, what happens to the covalent conjugates if you remove the two G domain cysteines, or the three E-domain cysteines?

ANSWER

We thank Reviewer#3 for this important comment. To answer the question, which cysteines are involved in the disulfide bridge formation, we performed an additional experiment, expressing cysteine variants in HEK293T gephyrin knockout cells and attempted to measure redox-dependent multimers in WB after non-reducing SDS-PAGE. We included two new variants, one harboring cysteines only within the G-domain ("onlyG") and one harboring cysteines only within the E-domain ("onlyE"). As mentioned above, in cultured cell, we could

not convincingly resolve sharp multimeric gephyrin bands. However, we observed a smear in the higher molecular weight region for gephyrin wt and not for cys-free (see image below, analysis: Total upper lane intensities normalised to gephyrin band at 130 kDa). Interestingly, based on all variants analyzed, the smear was only observed when cysteines in gephyrin's E-domain were present.

Due to the lack of a defined gephyrin multimer, we feel that these results are not of high enough quality and did not include these in the current manuscript.

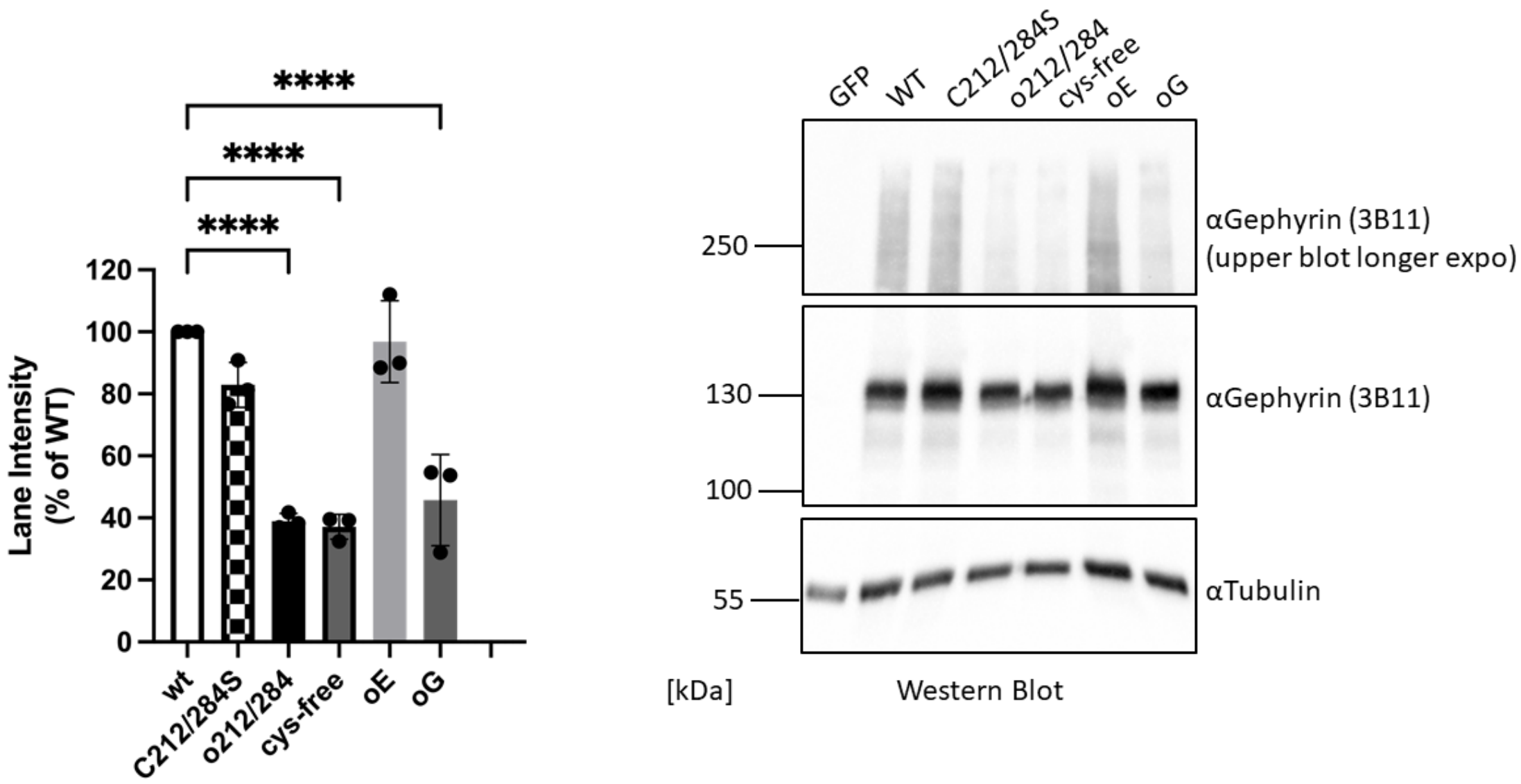

I find it unfortunate that the mutant lacking all exposed cysteines is called "Cys-free", although it still contains the buried cysteines. Perhaps a better nomenclature can be found?

ANSWER

We agree with the reviewer that "cys-free" could suggest that internal cysteines could have been changed as well. However, in keeping with a short and not overly complicated term, we feel that "cys-free" is the best solution. We clearly define in the text which residues have been exchanged

To test whether more than one cysteine outside the palmitoylation motif may have a functional role, we generated a gephyrin variant containing only Cys212 and 284 (o212/284) by replacing all other surface cysteines (Cys26, 154, 293, 419, 469, 663) with a serine. Additionally, we generated variants with remaining Cys212 (o212) and Cys284 (o284), respectively, and one variant without any surface-exposed cysteine, which we called "cys-free". Importantly, in all variants the four internal cysteines (Cys74,133, 635, 676) remained unchanged.

Fig. 1E

The labelling of the gels (minimum/maximum/steady state) is somewhat confusing; it would be much clearer to refer to the actual treatments (-PEG/-SDS; +PEG/+SDS; +PEG/-SDS).

ANSWER

We agree and added the requested labels directly to the Figure.

Fig. 3A

As expected, the strong oxidant diamide crosslinks accessible thiols into disulfides, thus generating higher order covalent aggregates. This experiment confirms the notion that gephyrin has surface exposed thiols. I am not sure what else can be concluded here about gephyrin. Wouldn't one expect that any protein with multiple exposed thiols would show the same result? Please clarify.

ANSWER

This experiment confirms the notion that gephyrin has surface exposed cysteines and that these cysteines can form intermolecular disulfide bonds. Disulfide formation efficiency is influenced by cysteine proximity and the overall protein structure. As outlined below, we now present additional results demonstrating that diamide can be replaced by H_2O_2.

Do the authors believe/assume that gephyrin is directly oxidized by H2O2 in cellulo? In that case it would be logical to use H2O2 rather than the non-natural (and much stronger) thiol oxidant diamide. How much H2O2 do you need to get the same degree of aggregation as with diamide?

ANSWER

We thank the reviewer for proposing this additional experiment. We performed experiments in the presence of 1 mM H_2O_2 (incubation 18 h @ 4 °C) and observed similar behavior as in the presence of diamide. These new experiments have been added to the manuscript (Figure S5 C,D).

Fig. 4A/B

Naturally, the oxidized (disulfide-crosslinked) gephyrin likes to sediment (pulling the interacting pentameric receptor with it). Does this qualify as a demonstration of phase separation? Please justify.

ANSWER

As indicated by our novel experiments using an established model system for phase separation (GCN4-GABA$_A$Rα1, GCN4-GABA$_A$Rα3, and GCN4-GlyR), we demonstrate that gephyrin oxidation does not result in increased sedimentation per se (a simple assay used to demonstrate phase separation behavior). For example, oxidized gephyrin did not show increased sedimentation with the GCN4-GlyR model system, while increased sedimentation was observed together with GCN4-GABA$_A$Rα1 and GCN4-GABA$_A$Rα3.

Additionally, oxidized gephyrin alone remained soluble and was recovered from the supernatant and not from the pellet fraction (control lanes in Figure 4A and Figure S7A,B). Below there are additional controls that indicate no difference between reduced and oxidized gephyrin alone in their sedimentation behavior.

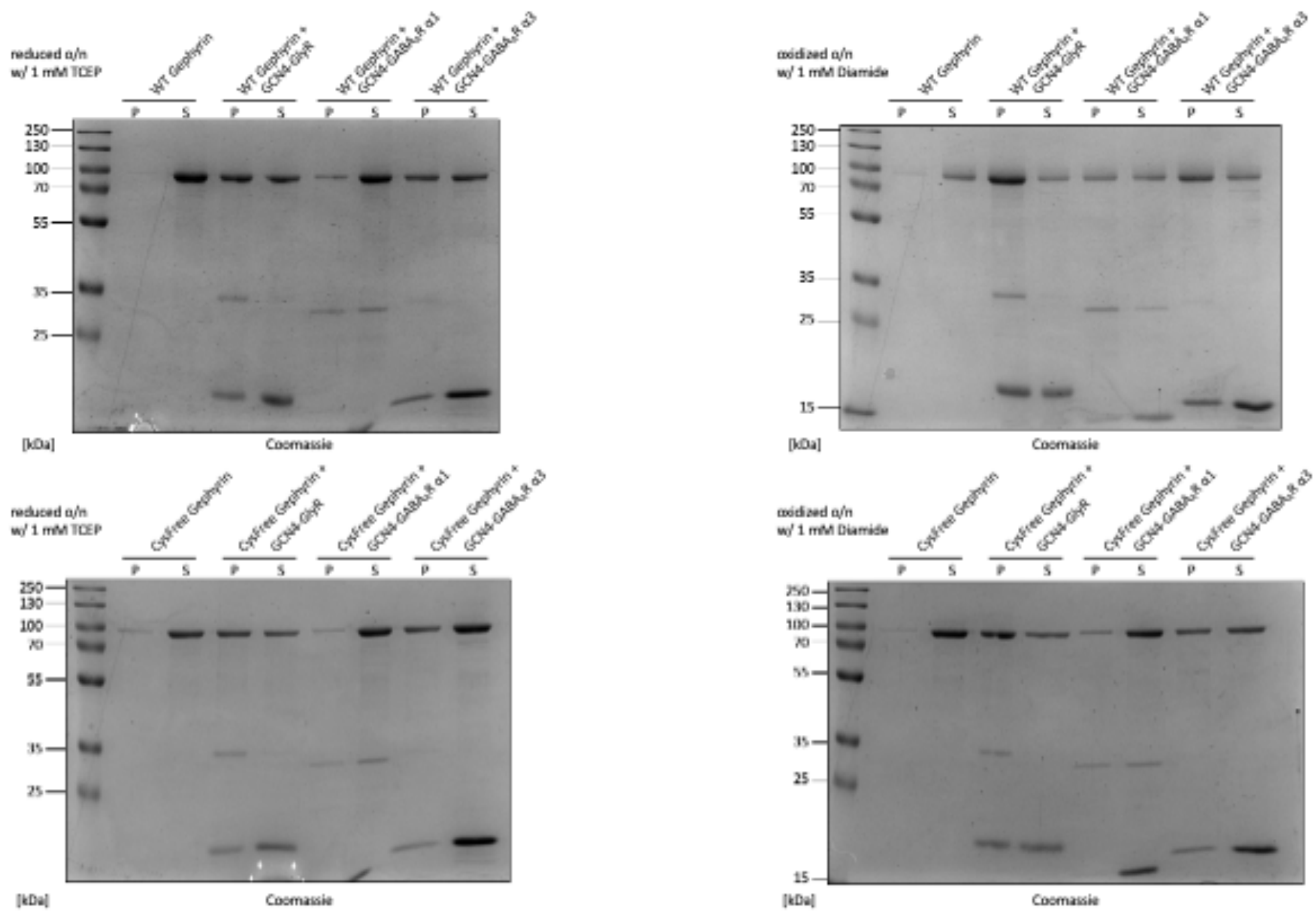

Figs. 5/6

It is nice to see that endogenous generation of H2O2, directly (DAO) or indirectly (complex III inhibition) impacts gephyrin and its interaction at the synapse. I am just wondering if the amount of H2O2 produced under these circumstances can be considered close to physiological. Please clarify.

ANSWER

A previous study has measured that DAAO expression in mammalian cells that were treated with mM concentrations of D-amino acids produce between 50 – 100 nM H_2O_2 (Matlashov et al, ARS 2014). These concentrations can be considered to be in the physiological range (Sies and Jones Nature Reviews Molecular Cell Biology 2020).

A concentration of 10 µM external H_2O_2 is considered to be in the range where HEK293T cells transition from signaling to stress response. For DAAO localized to mitochondria, approximately 4 mM D-Ala yields similar levels of intracellular H_2O_2. However, it is important to note that the response curves between extracellular and intracellular H_2O_2. are different.

Would it be possible to also demonstrate in these experiments the formation of covalent oligomers on a gel (ideally by titrating the L-aa or the ETC inhibitor) ?

ANSWER

As outlined above, we could not convincingly detect covalent oligomers from cultured cells.

When cells are treated with H2O2 from outside, does this induce/increase gephyrin crosslinking? How much H2O2 do you need in this case? Do you need mM concentrations, or just low micromolar concentrations?

ANSWER

Our dissociated neuronal cultures did not tolerate high µM H_2O_2 concentrations and as outlined above we cannot detect sharp bands for covalent gephyrin multimers from cultured cells.

It would be great if one could demonstrate the relationship between gephyrin thiol oxidation state and its clustering in cells undergoing physiological changes (e.g. a change in metabolism or activation/stimulation).

ANSWER

We agree with the reviewer that such experiments would strengthen the findings from our manuscript. At this stage, these experiments are beyond the scope of the manuscript.

Fig. S3C

In the text (lines 127-129) it has been said that for all variants "we observed similar… functional enzymatic activity", but in the figure, the cys-free variant has significantly reduced Moco activity.

Later in the text (line 150) it is finally stated that the cys-free variant is significantly impaired, but now there is no reference to the figure.

The context of these experiments may need some explanation. Why is Moco activity tested? Is it relevant for the scaffolding function?

ANSWER

We thank the reviewer for pointing our inaccuracy out to us. We corrected this statement in the text. Previous studies have established that Moco activity is independent from the scaffolding function (Smolinsky et al. JBC 2008, Dejanovic et al. EMBO Molecular Medicine 2015, see Figure EV3 on gephyrin D580A).

Fig. S5A/B

I have trouble understanding these gels. S5A: What is the prominent HMW band in lane 2 (peak 2 of reduced wt)? S5B: Why are there multimer band in wt-rd-2? And why are there multimer bands in cys-free-ox-2? Why is it stated that there is no difference between the oxidized/reduced sample of cys-free gephyrin (line 175)? Please clarify.

ANSWER

We agree with the reviewer that these gels are not convincing and sort of confusing. We intended to show the disappearance of the monomeric WT gephyrin band under oxidizing conditions, while this is not the case in the cys-free variant. We have removed those gels and added new panels of our size exclusion chromatography study under oxidizing (H_2O_2), reducing and combined conditions (New Fig. S5).

General comments:

The conclusions of the paper should be phrased more carefully.

The abstract states that mitochondria-derived ROS were identified as a physiological source of gephyrin oxidation. The antimycin experiment suggests that mitochondria can (in principle)

trigger gephyrin oxidation, but how do we know that mitochondrial oxidants are driving gephyrin oxidation under physiological conditions?

ANSWER

As outlined to the first point raised by reviewer #1, the Antimycin A treatment is not a good tool to demonstrate that indeed under physiological conditions mitochondria-derived ROS can trigger synaptic effects. As described by Larson et al. in JNeurosci 2020, for the strengthening of α3-containing GABAARs synapses, this effect can be elicited by mitochondria-derived ROS (using Antimycin A), while under physiological conditions mitochondria-derived ROS is not required for the Nox2-dependent pathway. To clarify our findings, we removed the Antimycin A experiment from the manuscript and put additional focus on functional data obtained from electrophysiology.

There is a lot of emphasis on mentioning/implicating LLPS, but little data relating to this aspect. I would recommend to tone this down.

ANSWER

While we expanded the explanation on LLPS in the introduction (see reviewer #2, Results section, specific point 4.) to define more precisely its meaning in the context of synapses and added half a paragraph on Neuroligin 2 in the discussion (see comment from reviewer #1), we now removed a paragraph from the discussion describing technical challenges to study LLPS at synapses. Given the additional experiments using different LLPS ligands, we feel that this is now given a reasonable weight.

The actual mechanism of gephyrin oxidation seems to be beyond the scope of the current manuscript, but should at least be briefly discussed. For example, it is not obvious that gephyrin is directly oxidized by H2O2 (although that is a possibility). Alternatively, oxidation may be caused by another oxidant species, potentially derived from H2O2, or through mediation by a redox enzyme (oxidoreductase, peroxidase).

ANSWER

We thank the reviewer and added a sentence to the discussion:

"Furthermore, we did not answer the question whether gephyrin was directly oxidized by H_2O_2 or other oxidant species, which could be derived from H_2O_2."

Answer in yellow (similar to all corrections in the main manuscript)

Reviewer #3 (Remarks to the Author):

The manuscript has been improved, and many of the previously raised questions have been further clarified. I am generally pleased to see this study moving toward publication.

However, from my perspective, the redox biochemistry of gephyrin within cells remains rather ill-defined. The formation of disulfide-linked oligomers is demonstrated exclusively in NEM-perfused brain tissue, but not in any of the cell-based experiments.

In their response, the authors note that they were unfortunately unable to detect covalent gephyrin multimers in their cultured cells. I find this both puzzling and concerning. I do not believe this point should be treated as a minor or easily explained detail.

All cell-based experiments (cluster analysis, patch clamp; with and without additional H_2O_2 generation by DAO) are built on the premise that gephyrin forms intermolecular disulfide bonds. Based on this, one would reasonably expect it to be possible (using appropriate alkylation and extraction protocols) to demonstrate a correlation between gephyrin disulfide crosslinking and the observed phenotypes, such as changes in cluster size. In my view, this remains a gap in the argument of the study.

In their rebuttal, the authors propose that in brain tissue obtained from adult animals, disulfide bond formation may be favored, whereas in cultured hippocampal neurons, sulfenylation could predominate. However, it remains unclear how this would explain the observed cellular effects. Are the authors suggesting that sulfenylation promotes non-covalent gephyrin oligomerization such that covalent crosslinks are not necessary? Or that sulfenylation bypasses the need for oligomerization altogether? Both interpretations seem unlikely and are, in any case, at odds with the central narrative of the paper, which begins with the observation of covalent gephyrin oligomers in brain extracts and then demonstrates disulfide-linked multimers in vitro. The manuscript clearly leads the reader to the conclusion that thiol oxidation results in disulfide formation, which stabilizes gephyrin oligomers at the membrane.

As far as I can tell, it is not clearly stated in the manuscript that covalent gephyrin conjugates could not be detected in the cellular models used. Instead, the authors employ the more general term “thiol oxidation” in place of “disulfide bond formation,” to reflect the absence of direct evidence for disulfides in cells. I believe this issue should be addressed more explicitly and transparently in the discussion.

ANSWER:
We thank the reviewer for this important and constructive comment, which has helped us to substantially improve both the clarity and conceptual consistency of the manuscript. We fully agree that the redox biochemistry of gephyrin in cellular systems must be presented in a more precise and transparent manner, particularly with respect to the role of cysteine oxidation versus disulfide bond formation.

In response, we have revised the manuscript at multiple points, as detailed below, to avoid overemphasizing disulfide bridge formation where direct evidence in cellular systems is lacking. We now included clear data as supplementary **Fig. S5A-C** showing that in different cellular systems we did not detect any significant levels of high molecular weight disulfide-bridge cross-linked gephyrin species.

Specifically, we have adjusted the overall narrative to consistently focus on cysteine-dependent thiol oxidation as the central mechanism, rather than implying disulfide formation as a general explanation and principle underlying the observed oxidation-dependent effects.

Importantly, and after completing our own structural studies in gephyrin LLPS (Macha *et al.*, 2025) we have included an additional LLPS dataset (**Fig. 4C,D and S7C,D**), as already suggested by the reviewer in her/his first evaluation, in which redox modulation was narrowed down to cysteines within the gephyrin E-domain. These data demonstrated that it is Cys419, located at the filament-forming SDII–SDII′ GephE interface, which plays a major role in regulating LLPS.

Based on our observations in brain extracts, we have revised the corresponding statement to adopt a more cautious and accurate tone, focusing on functional consequences rather than a specific chemical mechanism by now saying:
“Based on this finding, we hypothesized that gephyrin cysteines are redox-sensitive and that thiol oxidation modulates intermolecular interactions, thereby influencing gephyrin multimerization, which is a crucial parameter for its synaptic function”

Furthermore, we have completely rewritten the *in vitro* section to improve the logical flow and to clearly guide the reader from the initial observations in brain tissue to the rationale for the following set of biochemical experiments. In this revised version, we explicitly distinguish between evidence for disulfide-linked species in brain extracts and the broader concept of cysteine-dependent redox modulation of gephyrin multimerization observed *in vitro*, which remained clearly reversible in the presence of reductant.

Most importantly, we have added two new paragraphs to the Discussion and revised **Figure 7** to specifically address the reviewer’s concern. In these sections, we now explicitly state that we do not detect covalent gephyrin multimers in neuronal or non-neuronal cellular systems. We further expand on alternative mechanisms of thiol oxidation, including a model in which oxidation of Cys419 controls the SDII–SDII′ interface via electrostatic stabilization, thereby promoting multimerization and LLPS without requiring covalent disulfide-based crosslinking.

Together, these revisions clarify the mechanistic interpretation of our data and ensure that the manuscript no longer implies the presence of disulfide bonds in cellular systems where they cannot be demonstrated, while still providing strong and comprehensive arguments for the central role for cysteine-dependent redox regulation in gephyrin organization at inhibitory synpases.

Reference:
Macha, A. *et al.* (2025) “Gephyrin filaments represent the molecular basis of inhibitory postsynaptic densities,” *Nature Communications*, 16(1), p. 8293. Available at: https://doi.org/10.1038/s41467-025-63748-w.
