# Supplementary material for: Redox-dependent synaptic clustering of gephyrin
Source: Nat Commun. 2026 Sep 3;17:9457. doi: 10.1038/s41467-026-77147-2 (PMC13542264; doi:10.1038/s41467-026-77147-2)
Supplement: Supplementary file 4 [file 41467_2026_77147_MOESM4_ESM.zip › 4_Source Data/1/A/1A Lower GAPDH Blot.pdf]

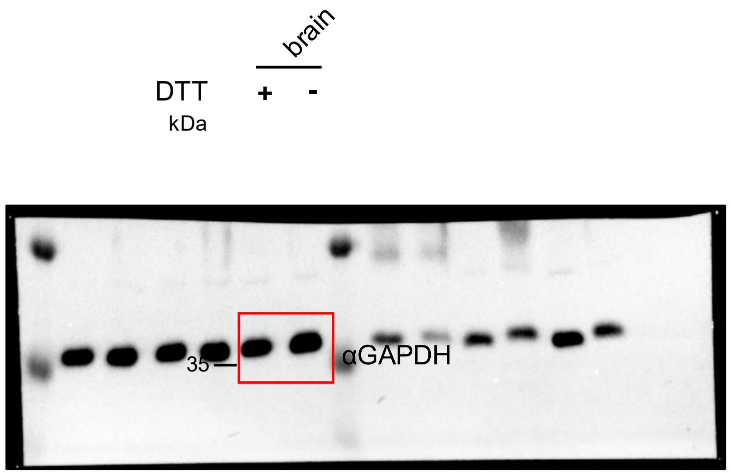
brain
DTT
+
-
kDa
35
αGAPDH
