# Supplementary material for: Redox-dependent synaptic clustering of gephyrin
Source: Nat Commun. 2026 Sep 3;17:9457. doi: 10.1038/s41467-026-77147-2 (PMC13542264; doi:10.1038/s41467-026-77147-2)
Supplement: Supplementary file 4 [file 41467_2026_77147_MOESM4_ESM.zip › 4_Source Data/1/E/1E Middle Gel.pdf]

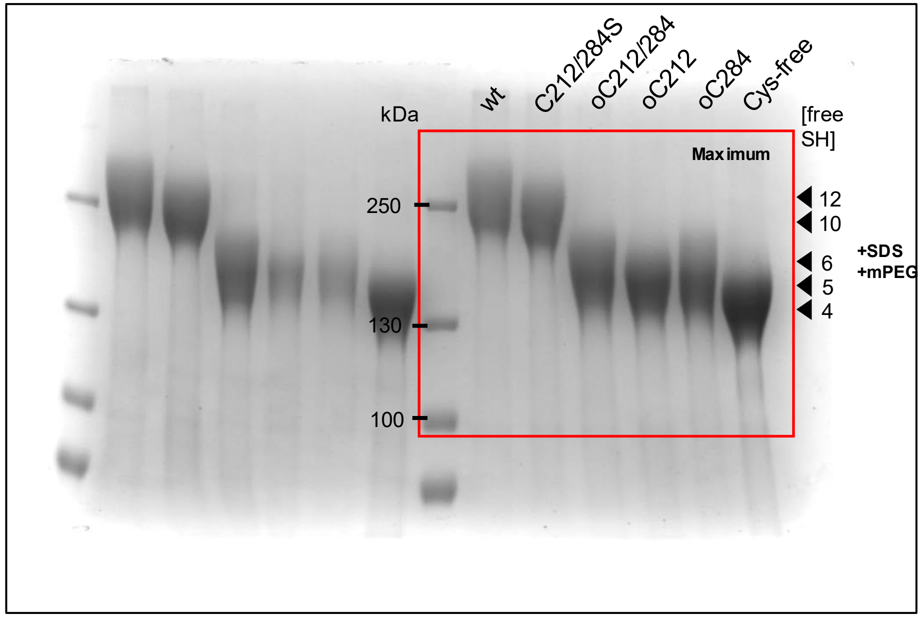

wt
C212/284S
oC212/284
oC212
oC284
Cys-free
kDa
250
130
100
[free SH]
Maximum
12
10
6
5
4
+SDS
+mPEG
